# Differential Allelic Expression in the Human Genome: A Robust Approach To Identify Genetic and Epigenetic *Cis*-Acting Mechanisms Regulating Gene Expression

**DOI:** 10.1371/journal.pgen.1000006

**Published:** 2008-02-29

**Authors:** David Serre, Scott Gurd, Bing Ge, Robert Sladek, Donna Sinnett, Eef Harmsen, Marina Bibikova, Eugene Chudin, David L. Barker, Todd Dickinson, Jian-Bing Fan, Thomas J. Hudson

**Affiliations:** 1 McGill University, Montreal, Quebec, Canada; 2 Genome Quebec Innovation Centre, Montreal, Quebec, Canada; 3 Illumina Inc., San Diego, California, United States of America; The Wellcome Trust Sanger Institute, United Kingdom

## Abstract

The recent development of whole genome association studies has lead to the robust identification of several loci involved in different common human diseases. Interestingly, some of the strongest signals of association observed in these studies arise from non-coding regions located in very large introns or far away from any annotated genes, raising the possibility that these regions are involved in the etiology of the disease through some unidentified regulatory mechanisms. These findings highlight the importance of better understanding the mechanisms leading to inter-individual differences in gene expression in humans. Most of the existing approaches developed to identify common regulatory polymorphisms are based on linkage/association mapping of gene expression to genotypes. However, these methods have some limitations, notably their cost and the requirement of extensive genotyping information from all the individuals studied which limits their applications to a specific cohort or tissue. Here we describe a robust and high-throughput method to directly measure differences in allelic expression for a large number of genes using the Illumina Allele-Specific Expression BeadArray platform and quantitative sequencing of RT-PCR products. We show that this approach allows reliable identification of differences in the relative expression of the two alleles larger than 1.5-fold (i.e., deviations of the allelic ratio larger than 60∶40) and offers several advantages over the mapping of total gene expression, particularly for studying humans or outbred populations. Our analysis of more than 80 individuals for 2,968 SNPs located in 1,380 genes confirms that differential allelic expression is a widespread phenomenon affecting the expression of 20% of human genes and shows that our method successfully captures expression differences resulting from both genetic and epigenetic *cis*-acting mechanisms.

## Author Summary

We describe a new methodology to identify individual differences in the expression of the two copies of one gene. This is achieved by comparing the mRNA level of the two alleles using a heterozygous polymorphism in the transcript as marker. We show that this approach allows an exhaustive survey of cis-acting regulation in the genome; we can identify allelic expression differences due to epigenetic mechanisms of gene regulation (e.g. imprinting or X-inactivation) as well as differences due to the presence of polymorphisms in regulatory elements. The direct comparison of the expression of both alleles nullifies possible trans-acting regulatory effects (that influence equally both alleles) and thus complements the findings from gene expression association studies. Our approach can be easily applied to any cohort of interest for a wide range of studies. It notably allows following up association signals and testing whether a gene sitting on a particular haplotype is over- or under-expressed, or can be used for screening cancer tissues for aberrant gene expression due to newly arisen mutations or alteration of the methylation patterns.

## Introduction

Understanding the genetic causes of phenotypic variation in humans still remains a major challenge for human genetics. In hundreds of cases, a single DNA sequence polymorphism affecting a protein coding sequence has been linked to a clear simple Mendelian phenotype (see e.g. [1]) and, for a much smaller but increasing number of cases, to more complex phenotypes [2]–[4]. Recent developments in high-density genotyping technologies have led to the completion of several whole genome association studies that test hundreds of thousands of markers for a specific disease. While earlier studies essentially focused on variants in coding sequences and regions immediately surrounding candidate genes, whole genome scans interrogate, in an unbiased way, most of the human genome including large regions of non-coding DNA that had not been studied previously. Interestingly, some of the strongest signals observed in these association studies are located in non-coding regions, either in large introns (e.g. [5]–[7]) or far away from any annotated loci (e.g. [8] and references therein). The mechanisms connecting these polymorphisms to the etiology of the diseases are still unclear but regulation of gene expression remains an obvious candidate. It is thus becoming particularly important to have a powerful and reliable method to easily test the influence of DNA polymorphisms on gene expression. One of the approaches commonly used to identify regulatory polymorphisms is to look for statistical associations between variation in gene expression and individual genotypes [9],[10]. This method offers the advantage of simultaneously analyzing thousands of genes using gene expression arrays and has yielded fascinating results in yeast [11],[12] and mouse [13]–[16]. Its application in humans [17]–[24] suffers from relatively low statistical power due to potential inter-individual differences in a large number of causal variants involved in the regulation of a specific gene [25], their modest effects and the burden of the multiple testing correction necessary to take into account the large number of independent tests performed. In addition, since this approach requires extensive genotype information for all individuals, it is costly to apply to new samples. An alternative approach is to compare the relative expression of the two alleles in one individual: the effect of a polymorphism affecting in *cis* the regulation of a particular transcript can be detected by measuring the relative expression of the two alleles in heterozygous individuals using a transcribed SNP as a marker [26]–[32]. Several studies have used this approach in humans but have been criticized for their low throughput or the apparent high variability.

Here we describe a novel array-based method that allows high-throughput assessment of differential allelic expression. We used a modified version of the Illumina GoldenGate genotyping platform, the Allele-Specific Expression (ASE) assay, to assess the extent of differential allelic expression for over 1300 genes in more than 80 human lymphoblastoid cell lines (LCLs). Our analyses include 352 genes located in ENCODE regions and chromosome 21 that have been previously screened for *cis*-regulatory polymorphisms using total gene expression [19]. This allows us to directly compare the advantages and drawbacks of the two approaches in terms of range, sensitivity and robustness. We specifically address the issue of experimental noise and reproducibility of the findings and show that biology, not experimental variability, is responsible for the patterns observed. We discuss the relevance of our results for the identification of the molecular mechanisms regulating gene expression, as well as their implications for future genetic studies.

## Results

### Assessment of Differential Allelic Expression Using Illumina ASE Assay

**Figure 1:**
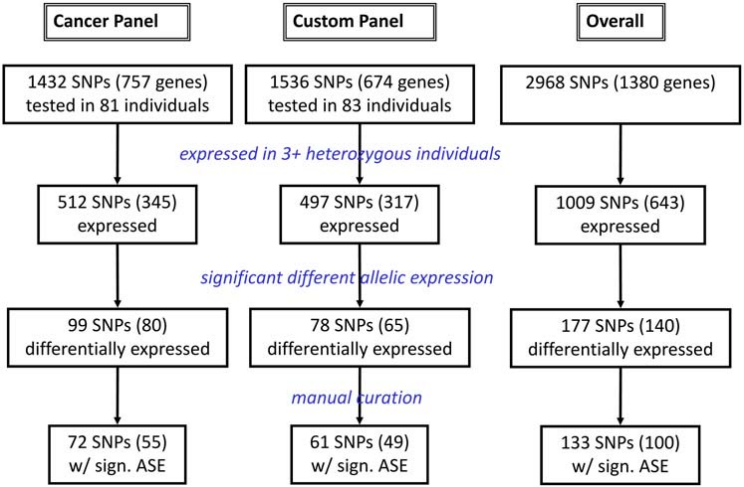
Experiment design and results obtained for the two panels used in the study. The Overall column corresponds to the combination of the two panels. The detailed composition of each panel is presented on Supplemental Table S1.

**Figure 2:**
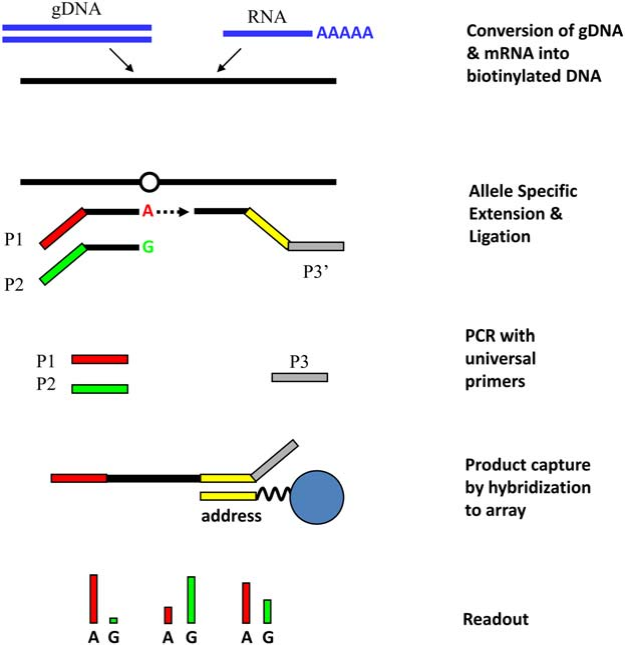
Overview of the Illumina Allele-Specific Expression assay. Genomic DNA and total RNA are separately converted into biotinylated DNA and amplified using fluorescence-labeled universal primers following extension and ligation of allele-specific assay oligo-nucleotides. PCR products are captured by locus-specific beads and the fluorescence of each dye (i.e. allele) at each locus is measured by quantitative fluorescence imaging (see Materials and Methods for details).

We first assessed the extent of differential allelic expression at 1,432 exonic SNPs using 81 individual LCLs with the Illumina ASE technology (Figure 1 and Table S1 for the composition of the Illumina ASE Cancer Panel). This technology uses primer extension assays with fluorescence-labeled allele-specific primers to measure the proportion of each allele separately at the genomic and transcriptomic levels (Figure 2). Five hundred and twelve SNPs (in 345 genes) displayed an expression level significantly higher than background in at least three heterozygous individuals and were further investigated (see Materials and Methods for details). The extent of differential allelic expression at each SNP was obtained by comparing the relative amount of each allele in RNA to the ratio observed in DNA.

**Figure 3:**
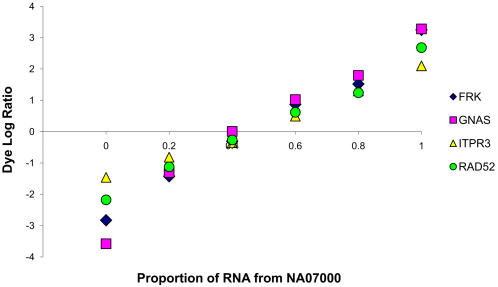
Result of the spike mixes experiment for four genes. The graph shows the logarithm of the dye ratio (y-axis) for four mixes of total RNA extract (x-axis) of NA12155 (at 0 on the x-axis) and NA07000 (at 1 on the x-axis). The original RNA extracts mixed and the four mixes for FRK (rs580396) are shown in blue, for GNAS (rs7121) in pink, for ITPR3 (rs2229634) in yellow and for RAD52 (rs1051672) in green.

As a first effort to determine if the assay could reliably be used to assess differential expression we generated spike mixes using varying proportions of total RNA extracts from two individuals. For 20 exonic SNPs located in expressed transcript, the two individuals are homozygous for the different alleles (i.e. respectively AA and BB), while for 192 SNPs one individual is heterozygous and the other homozygous (i.e. either AB and AA, or AB and BB). Since the expression of each gene may differ between the two individuals, one does not expect to observe an exact translation of the proportions of total RNA mixed to the “allelic” expression level. However, the allelic expression differences estimated for the different spike mixes should be the proportional to each other. For all homozygous/homozygous mixes (20 out of 20 SNPs) and 83% of the heterozygous/homozygous mixes (159 out of 192), we observed a significant linear correlation (p<0.05) between the proportion of mixed total RNAs and the “allele-specific expression” estimated by the assay (Figure 3 and Figure S1).

**Figure 4:**
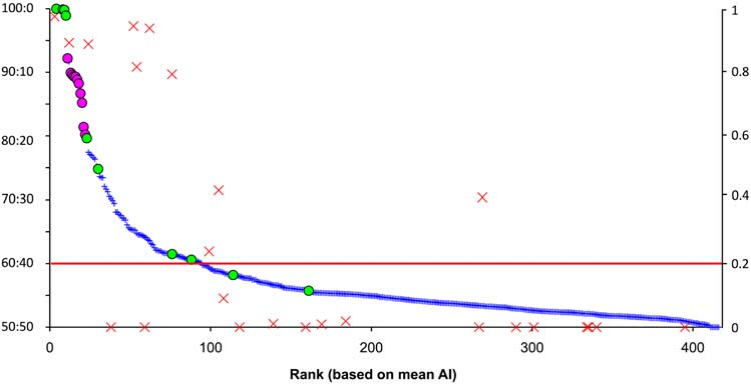
Population-average estimates of allelic expression at 416 SNPs from the first panel. Each blue cross stands for one SNP and is displayed on the left y-axis based on the average allelic ratio observed using all heterozygous individuals for this SNP. The SNPs are ranked on the x-axis from 1 (SNP with the highest average allelic imbalance) to 416 (lowest average allelic imbalance). Green dots stand for SNPs in imprinted genes, pink dots for SNPs in X-linked genes. The red line represents our significance threshold based on the estimation of experimental variability and corresponds to an average allelic ratio of 40∶60. The red crosses are the 25 SNPs also analyzed using quantitative sequencing of RT-PCR products and are displayed on the right y-axis according to the strength of the correlation (Pearson's r^2^) between the estimates of both methods (i.e., 1 indicates complete correlation between both estimations, 0 no correlation). Non significant correlations (p>0.05) are indicated with r^2^ = 0.

We then tried to assess the threshold above which differential allelic expression would be genuine: even if the two alleles are equally expressed in one individual, we expect the ratio of allelic expression measured at a given marker to deviate stochastically from 50∶50 due to experimental variability. In order to differentiate technical noise from biological signal (i.e. the differences in allelic expression due to differential *cis*-acting regulation), we evaluated the extent of experimental variability in the assay by comparing independent estimates of allelic imbalance for duplicates of individual RNA. We used duplicated measurements from 81 individuals at all SNPs expressed to determine a robust estimation of the experimental variability (N =  31,503 duplicates). After averaging duplicate differences for each SNP over all individuals, we observed than less than 3% of the SNPs show a population average variability greater than 10% (see Materials and Methods for more details and Figure S2). This level of experimental variability corresponds to a ratio of allelic expression of 60∶40 (i.e. 1.5-fold difference). Thus, population-average allelic expression ratio at any SNP lower than 60∶40 can be explained by experimental noise, while a SNP displaying a population-average differential allelic expression greater than this threshold most likely reflects a biological process affecting *cis*-regulation. We are interested in the present study in identifying loci with common allelic expression differences and we thus focused on population-average differential allelic expression: the average over all heterozygous individuals of the extent of allelic expression differences, regardless of which allele is over expressed (this is addressed later). The identification of a single individual with dramatic allelic expression difference is also possible using the same approach (but a different detection cut-off) but is beyond the scope of this paper. Among the 345 genes expressed in this first panel (512 SNPs), 72 (87 SNPs) displayed an average level of allelic imbalance larger than this 40∶60 cut-off and were thus considered to display significant differences in allelic expression (Figure 4).

These analyses rely on the observation of the three genotypes in the population (i.e. AA, AB and BB). To also include SNPs with a lower minor allele frequency for each it was not possible to observe homozygotes for the minor allele in our small sample, we designed a second analysis method using solely the heterozygous individuals. If the alleles are differentially regulated we expect to observe in some cases a very large variance in the ratio of the two alleles in the population. We used this approach to determine SNPs for which the heterozygous individuals harbor a variance of the allelic ration higher than expected using a Maximum expectation algorithm (see Materials and Methods for details). This approach does not allow us to quantity the overall extent of differential allelic expression but identifies 8 genes with differential allelic expression that were not identified by the previous method.

**Figure 5:**
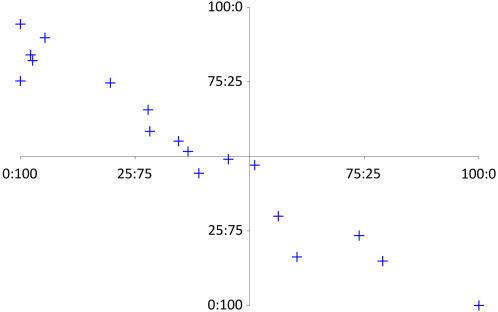
Estimates of allelic expression using two SNPs located in the IL1A gene. Each blue cross displays one individual heterozygous at two SNPs in IL1A. The x-axis represents the estimate of allelic imbalance using rs1304037, the y-axis the allelic imbalance measured using rs17561. The two axes cross at 50∶50 corresponding to an equal expression of both alleles, the allelic ratio 100∶0 corresponds to complete transcriptional silencing of one allele, 0∶100 to the silencing of the other allele.

When one considers the estimates of allelic expression obtained using different SNPs in a same transcript showing significant differences in allele expression (i.e. with a population-average ratio greater than 60∶40), we note that 36 out of 44 correlations between individuals estimates are significant (for an average r^2^ of 0.83). Individuals showing a large allelic expression difference at one SNP display similar patterns at all heterozygous positions of the transcript (an example is shown on Figure 5). This observation supports our findings that the experimental variability is low in the Illumina ASE assay and that this assay allows quantitative assessment of differential allelic expression. Consequently, the population-average estimates of allelic imbalance obtained with different markers in the same transcript tend to be similar (Table S2) but can vary since different individuals will be included in the average (depending on whether they are heterozygotes at this marker).

### Validation of Allelic Imbalance Estimates Using Quantitative Sequencing

**Figure 6:**
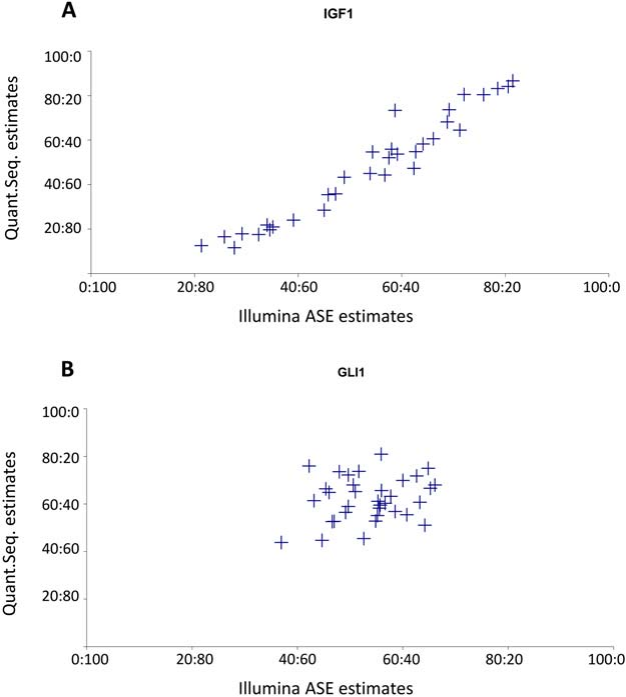
Cross-validation by quantitative sequencing. The correlations between the estimates of allelic imbalance using Illumina ASE assay (x-axis) and quantitative sequencing (y-axis) are shown for all individuals heterozygous for IGF1 (A) and GLI1 (B). IGF1 displays significant population-average allelic imbalance (with a mean allelic ratio of 60∶40) and the estimates from quantitative sequencing are very similar with those from Illumina. In contrast, GLI1 did not show significant allelic imbalance (the population-average ratio is close to 55∶45 below the significance threshold of 60∶40) and the estimates from quantitative sequencing of the same individuals are not correlated with those of Illumina (as expected if they only result from stochastic noise).

To further assess the validity of our results, we randomly selected 25 genes tested on the Illumina ASE platform and used quantitative sequencing of RT-PCR products [33] to measure allelic imbalance for the same SNP in the same individuals (Figure S3). The selected genes consisted of eight autosomal genes with significant allelic imbalance and 17 genes for which the level of differential allelic expression did not reach our significance threshold. We analyzed the same 81 individual LCLs using RNA from the same extract as for the Illumina assay. Overall, we observed a strong correlation between the estimates of allelic imbalance obtained for each individual using the two methods for the genes with a ratio of allelic expression larger than our 40∶60 cut-off (r^2^>0.8 for 6 out of 8 genes, see Figure 6A as a example). The correlations were not statistically significant for the genes for which the average difference in allelic imbalance did not reach our significance threshold (16 out of 17 genes, Figure 6B): minor deviations observed in the allelic ratio for these genes likely correspond to random variations and are therefore not expected to be reproducible. The strength of the correlation (measured by Pearson's r^2^) for all 25 genes is shown on Figure 4. One SNP in CD44 (rs8193) displayed a low but significant correlation between our estimates of allelic imbalance obtained from the Illumina assay and those using quantitative sequencing (p<10^−4^, r^2^ =  0.4075), even though the average level of allelic imbalance was below our significance cut-off on the Illumina platform. Allelic imbalance at CD44 has been previously reported [30] and it is likely that the signal observed at that gene is real but corresponds to a low level of differential expression. Two genes (ABL2, XRCC1) showed significant allelic imbalance in the Illumina ASE assay (with a mean allelic ratio of, respectively, 70∶30 and 65∶35) but were not validated by quantitative sequencing. Manual inspection of the Illumina results for these genes revealed that the allelic expression ratios were estimated using a small number of homozygotes for the minor allele (respectively, 1 and 2 individuals) which led to an incorrect estimation of the expected dye ratio for heterozygotes and to a general over-estimation of allelic imbalance. For further analyses, we manually curated the list of all genes with significant differential allelic expression to remove potential false positives due to low number of homozygous individuals.

### Reproducibility of Allelic Imbalance Measured in Cell Lines

Our study uses lymphoblastoid cell lines (LCLs) and it remains controversial whether culture conditions could artefactually generate differential allelic expression. We therefore tested whether allelic imbalance is influenced by harvesting the cells after different numbers of passages. This allowed us to control the effect of changes in the culture environment including pH, nutrient concentration and cell density at the time of harvest. We compared our estimation of allelic imbalance for three genes with significant population-average allelic imbalance in 47 individuals using recently thawed LCLs harvested after the 2^nd^, 4^th^ and 6^th^ successive passages (respectively, “growths” 1, 2 and 3). The correlations between the allelic imbalance estimations are displayed in Figure S4 for the comparison of growths 2 and 3. The estimations of allelic imbalance after different passages were very similar to each other (r^2^>0.9), supporting the idea that differential allelic expression is little influenced by variations in culture environment.

### Comparison of Differential Allelic Expression with Total Gene Expression Association

An alternative approach to identifying *cis*-regulatory polymorphisms is to test for statistical association in a population between total gene expression measurements and the genotypes at markers in or surrounding the transcript. Interestingly, one of the genes showing the most marked difference in allele expression in our analysis is one of the 14 genes identified by Cheung and colleagues in a previous genome-wide study [17]. One comprehensive analysis was recently conducted for 512 RefSeq genes in ENCODE regions and chromosome 21 using LCLs from 60 unrelated individuals genotyped by the HapMap project [19]. In order to compare the respective strengths and weaknesses of total gene expression mapping and differential allelic expression, we designed a second panel that includes SNPs in the same genomic regions to analyze the same individual LCLs (Figure 1 and Table S1 for details). Using the information from the HapMap phase I (release 16) to select common exonic SNPs, we were able to include 228 and 124 genes from, respectively, ENCODE regions and chromosome 21, while Stranger and colleagues selected 321 and 191 genes (after screening for genes with high variance in their expression among individuals, see Materials and Methods for details). From the regions analyzed by Stranger and al., two-hundreds and ninety SNPs (in 170 genes) showed an expression level significantly higher than the background in three or more heterozygous individuals and were further investigated. Forty-nine out of 170 genes show significant level of differential allelic expression including 6 out of the 21 genes identified by Stranger and colleagues and present in our panel. Additionally, TTC3 which shows significant association between total gene expression and genotypes in the study by Stranger et al. shows patterns of allelic expression consistent with differential allelic expression (including a very high correlation between the extent of differential allelic expression estimated using different SNPs) on the Illumina ASE assay, even though it did not pass the significance cut-off. Overall in this second panel, 497 SNPs in 317 genes were expressed in three or more heterozygous individuals (out of 1536 SNPs in 674 genes) and 78 SNPs in 65 genes showed a significant level of differential allelic expression (Figure 1).

### Intronic SNPs Can Be Used To Assess Differential Allelic

To test whether intronic SNPs could be used instead of exonic SNP, we included for each gene on the second panel one intronic SNP. In general, intronic SNPs were less successfully analyzed and passed our expression threshold only for genes highly expressed in LCLs (Figure S5). This finding is consistent with previous observations [30] and the low proportion of unspliced mRNA (heteronuclear RNA) in cells relative to spliced transcripts. If the intronic SNP of a gene was detected in the RNA extract, it typically yields estimates of differential allelic expression very similar with those obtained using exonic SNPs.

### Differential Allelic Expression in the Human Genome

Overall, 177 out of 1,009 expressed SNPs (in 140 out of 643 genes expressed, 22%) display population-average ratios of allelic expression larger than 40∶60 or an higher than expected variance in allelic expression among heterozygous individuals and are thus unlikely to result solely from stochastic variation in the experiment (Figure S6). Table 1 shows the 133 SNPs (100 genes) with significant allelic imbalance after manual curation to remove possible false positives due to a low number of individual homozygous for the minor allele (this list is likely over-conservative and the complete data is presented in Table S2).

**Table 1:** Genes with significant allelic expression difference.

rs	Gene	Panel	Chr	# Hets	Variance & Mean	Average AI
rs2292305	THBS1	Cancer	15	13	Mean shifted	NA
rs2288539	NR2F6	Cancer	19	18	Mean shifted	NA
rs2459216	OAT	Cancer	10	6	High variance	NA
rs3750105	PEG10	Cancer	7	8	High variance	NA
rs3745410	LILRB3	Encode	19	5	High variance	NA
rs2839500	TMPRSS3	Encode	21	13	High variance	NA
rs546782	FGF9	Cancer	13	10	High variance	NA
rs17756426	DDX43	Encode	6	6	High variance	NA
rs1893963	DSC2	Cancer	18	14	High variance	NA
rs9978281	C21orf7	Encode	21	12	High variance	NA
rs2834601	CLIC6	Encode	21	8	High variance	NA
rs2070807	DNASE1L1	Encode	X	5	High variance	NA
rs705	SNRPN	Cancer	15	42	High variance	95∶05
rs7810469	PEG10	Cancer	7	12	High variance	95∶05
rs13073	PEG10	Cancer	7	35	High variance	95∶05
rs2240176	FLJ35801	Encode	22	18		95∶05
rs311683	DDX43	Encode	6	15	High variance	90∶10
rs5956583	BIRC4	Cancer	X	21	High variance	90∶10
rs1056831	CHI3L2	Cancer	1	39		90∶10
rs1050757	G6PD	Encode	X	6	Mean shifted	90∶10
rs1474593	BIRC4	Encode	X	19	High variance	90∶10
rs8371	BIRC4	Cancer	X	20	High variance	85∶15
rs1800291	F8	Encode	X	6		85∶15
rs2734647	MECP2	Cancer	X	12	High variance	85∶15
rs1057403	BTK	Cancer	X	4		85∶15
rs1059701	IRAK1	Cancer	X	14	High variance	85∶15
rs700	BTK	Cancer	X	22	High variance	85∶15
rs9018	FHL1	Cancer	X	4		85∶15
rs2734647	MECP2	Encode	X	10		85∶15
rs3813455	GAB3	Encode	X	10	High variance	85∶15
rs5945431	PLXNA3	Encode	X	9	High variance	85∶15
rs5958343	BIRC4	Cancer	X	26	High variance	85∶15
rs5987266	PLXNA3	Encode	X	10	High variance	85∶15
rs311686	DDX43	Encode	6	17	High variance	85∶15
rs9856	BIRC4	Cancer	X	24	High variance	85∶15
rs6151429	ARSA	Encode	22	24		85∶15
rs17330644	BIRC4	Encode	X	15	High variance	80∶20
rs1050705	F8	Encode	X	12	High variance	80∶20
rs11887	VBP1	Cancer	X	12	High variance	80∶20
rs12877	DNASE1L1	Cancer	X	4		80∶20
rs10798	KCNQ1	Cancer	11	31	High variance	75∶25
rs6571303	CXorf12	Encode	X	8		75∶25
rs9394782	NCR2	Encode	6	15		75∶25
rs183436	ABCG1	Encode	21	36		75∶25
rs6691569	FCRL3	Encode	1	12		75∶25
rs17561	IL1A	Cancer	2	21	High variance	75∶25
rs2278699	ZAP70	Cancer	2	3		75∶25
rs8535	CHI3L2	Cancer	1	38	High variance	75∶25
rs1056825	CHI3L2	Cancer	1	38	High variance	75∶25
rs1304037	IL1A	Cancer	2	26	High variance	75∶25
rs1571858	GSTM3	Encode	1	17	High variance	75∶25
rs3817405	PLXDC2	Cancer	10	12	High variance	75∶25
rs10863	MEST	Cancer	7	29	High variance	70∶30
rs10336	CXCL9	Cancer	4	11	High variance	70∶30
rs5351	EDNRB	Cancer	13	22	High variance	70∶30
rs1022477	RIBC2	Encode	22	26		70∶30
rs6007897	CELSR1	Encode	22	10	High variance	70∶30
rs11264793	FCRL3	Encode	1	24		70∶30
rs4445669	IGSF4	Cancer	11	40	High variance	70∶30
rs4767884	PXN	Cancer	12	26		70∶30
rs1041985	CDH2	Encode	18	38		70∶30
rs140519	KLHDC7B	Encode	22	30		70∶30
rs17197	PTGER2	Encode	14	10		70∶30
rs1803965	MGMT	Cancer	10	5		70∶30
rs2837029	C21orf13	Encode	21	15	Mean shifted	70∶30
rs724558	SERPINB10	Encode	18	23		70∶30
rs6007594	C22orf8	Encode	22	28		70∶30
rs7561	LAMB1	Cancer	7	21	High variance	70∶30
rs165602	NEFH	Encode	22	6		70∶30
rs10593	ITGB1BP1	Cancer	2	23	High variance	65∶35
rs1042531	PCK1	Encode	20	9		65∶35
rs1025689	IL17RB	Cancer	3	29		65∶35
rs225334	TFF2	Encode	21	17		65∶35
rs17207369	LILRP2	Encode	19	18		65∶35
rs3856806	PPARG	Encode	3	5	High variance	65∶35
rs300239	ENC1	Cancer	5	27		65∶35
rs677688	IMPACT	Cancer	18	7		65∶35
rs6104	SERPINB2	Cancer	18	19	High variance	65∶35
rs8097425	SERPINB10	Encode	18	24		65∶35
rs1071676	IL1B	Cancer	2	29	High variance	65∶35
rs9612234	GNAZ	Encode	22	16		65∶35
rs2024233	WNT2	Encode	7	24	High variance	65∶35
rs7927012	TRIM6	Encode	11	30		65∶35
rs2024233	WNT2	Cancer	7	15	High variance	65∶35
rs162549	CYP1B1	Cancer	2	22	High variance	65∶35
rs2075760	PLSCR3	Cancer	17	19		65∶35
rs2832236	C21orf7	Encode	21	40	High variance	65∶35
rs15017	MOXD1	Encode	6	6	High variance	60∶40
rs1053474	IMPACT	Cancer	18	31		60∶40
rs7120209	TRIM6	Encode	11	13		60∶40
rs958	MAPK10	Cancer	4	28		60∶40
rs7914	MCAM	Cancer	11	31	High variance	60∶40
rs12593359	RAD51	Cancer	15	44		60∶40
rs1368439	IL12B	Encode	5	23		60∶40
rs1103229	PPIL2	Encode	22	26		60∶40
rs2839600	NDUFV3	Encode	21	17		60∶40
rs3734744	MOXD1	Encode	6	11		60∶40
rs8807	HLA	Cancer	6	15		60∶40
rs743616	ARSA	Encode	22	37		60∶40
rs6214	IGF1	Cancer	12	31	High variance	60∶40
rs2822445	RBM11	Encode	21	38		60∶40
rs4947963	EGFR	Encode	7	19		60∶40
rs5275	PTGS2	Cancer	1	41		60∶40
rs2257505	MGC33648	Encode	5	29		60∶40
rs1029365	FLJ21062	Encode	7	30		60∶40
rs2839536	TSGA2	Encode	21	19		60∶40
rs6518322	LOC284837	Encode	21	29		60∶40
rs2258119	C21orf91	Encode	21	25		60∶40
rs2229730	CSK	Cancer	15	4		60∶40
rs2206593	PTGS2	Cancer	1	10	High variance	60∶40
rs2829877	JAM2	Encode	21	17		60∶40
rs1053395	TUBB4	Cancer	19	32		60∶40
rs1044104	BMP6	Cancer	6	22		60∶40
rs1801719	F2R	Cancer	5	31		60∶40
rs235768	BMP2	Cancer	20	7	High variance	60∶40
rs2239730	ZNF215	Cancer	11	41		60∶40
rs2270121	GAS7	Cancer	17	38		60∶40
rs963075	SERPINB10	Encode	18	32	High variance	60∶40
rs4820268	TMPRSS6	Encode	22	38		60∶40
rs4798	ITGB1BP1	Cancer	2	34		60∶40
rs9782	ASCL1	Cancer	12	28		60∶40
rs180817	BCR	Cancer	22	15		60∶40
rs406271	TFRC	Cancer	3	33		60∶40
rs1476217	FGF2	Cancer	4	43	High variance	60∶40
rs2239731	ZNF215	Cancer	11	44		60∶40
rs10916	CYP1B1	Cancer	2	13	High variance	60∶40
rs2230033	KCNJ15	Encode	21	33		60∶40
rs3088440	CDKN2A	Cancer	9	31		60∶40
rs2855658	CYP1B1	Cancer	2	37	High variance	55∶45
rs14983	MMP7	Cancer	11	22	High variance	55∶45
rs3747676	FGF2	Cancer	4	42	High variance	55∶45
rs10502001	MMP7	Cancer	11	19	High variance	55∶45
rs2066575	DLEU1	Cancer	13	27	High variance	55∶45

#Hets: number of heterozygous individuals which express the transcript.

Variance & Mean: indicates whether the analyses of variance/mean allelic expression detected significant deviation of the expression of both alleles (see Materials and Methods for details).

Average AI: population-average difference in allelic expression using all individuals heterozygous at this position (rounded down). These values correspond to values reported on the y-axis on Figure 4 and Figure S6.

Many of the genes with the highest extent of allelic imbalance in LCLs are located on the X-chromosome. While it is known that one allele at most X-linked genes is silenced in females by inactivation of one entire chromosome [34],[35], we would expect that a polyclonal cell population (in which half of the cells inactivate one X chromosome and the other 50% inactivate the alternate X chromosome) would give a similar level of expression for both alleles. However, all X-linked genes on our two SNP panels (22 SNPs in 12 genes) were among the top 5% of genes with most dramatic allelic imbalance patterns. The extent of allelic imbalance at a given gene varies among individual LCLs but interestingly, the patterns of allelic imbalance are very consistent across genes for a given individual (Figure S7). Additionally, the inheritance of the expressed allele (determined, when possible, using the pedigree information for the two families included in this study) appeared random. It has been previously proposed that the extent of clonality of a cell line could explain the patterns of allelic imbalance at genes with random mono-allelic expression [30]: clonal cells will all have the same X chromosome inactivated and thus display very high ratios of allelic imbalance. In contrast, cell-lines composed of a polyclonal population of lymphoblasts will have one or the other of their X chromosomes inactivated in different cells and thus an apparent expression of both alleles (i.e., a low extent of differential allelic expression). Our observations at X-linked genes are consistent with this hypothesis and the biased clonality of these LCLs, which were created over 20 years ago and passaged numerous times (see also [30]).

The two autosomal genes displaying the most dramatic allelic imbalance patterns have previously been shown to be imprinted in humans: PEG10 [36] and SNRPN [37]. In addition, KCNQ1, MEST and ZNF215 which are imprinted in humans [38]–[40] also show significant differences in allelic expression (Table 1). The mode of inheritance of the expressed allele also corresponds, in each case, to what has been described for the expression of these genes: for PEG10 and SNRPN, heterozygous individuals express the paternally-inherited allele (i.e. maternally imprinted) while for KCNQ1 the maternally-inherited allele is expressed. Our limited pedigree information is not conclusive for MEST and ZNF215. The only other known imprinted gene analyzable in our panel, PLAGL1 [41],[42] did not pass the significance threshold (i.e. an allelic ratio greater than 60∶40) but shows a population average allelic imbalance larger than 55∶45 and a high correlation between the two SNPs analyzable in the panel (rs2076684 and rs9373409); therefore it likely represents a significant difference in allelic expression.

**Figure 7:**
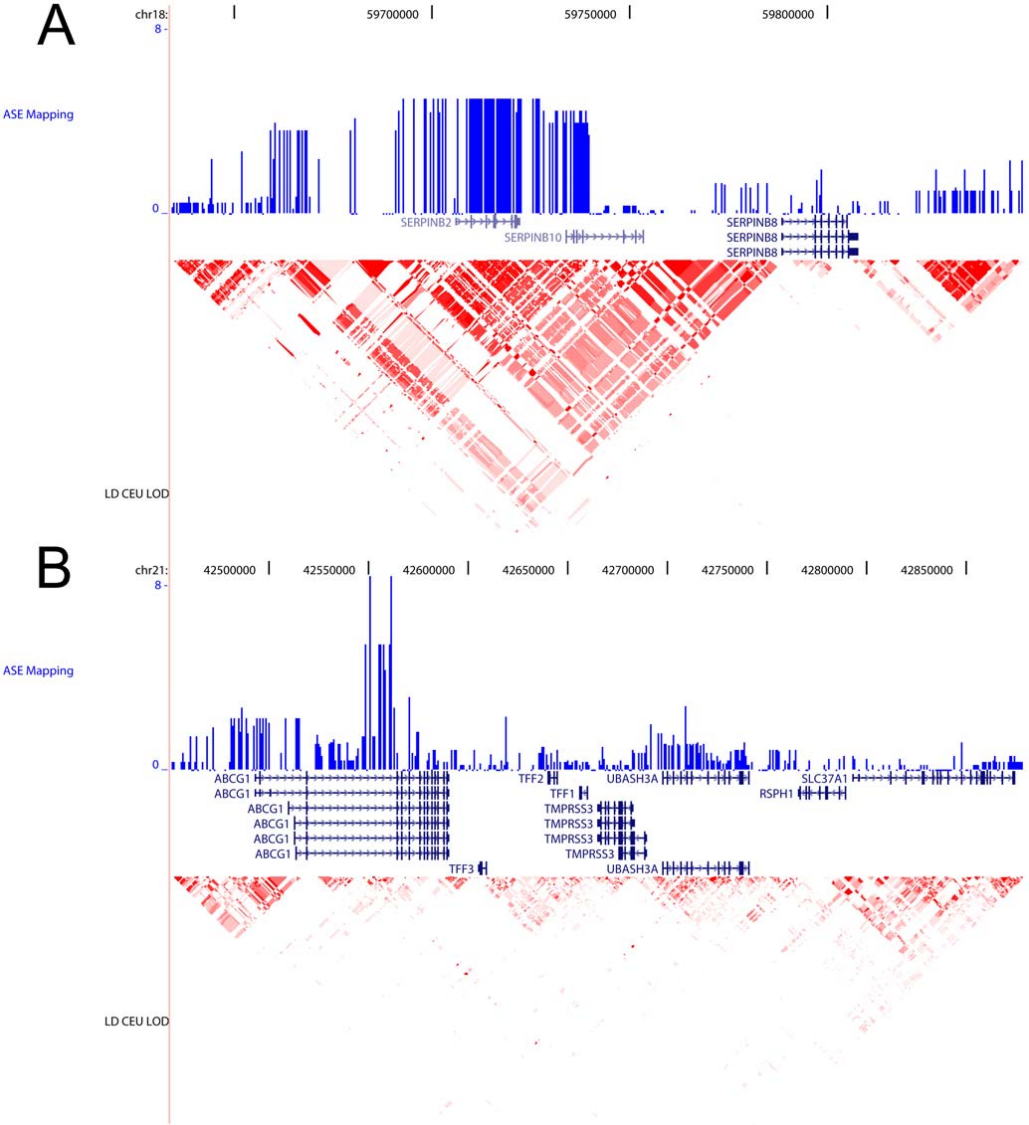
Association mapping of allelic imbalance to regulatory haplotypes for SERPINB10 (A) and ABCG1 (B). The green track shows the –log(p.value) for the association of the alleles for each SNP with the over-/under-expressing chromosome (i.e. the higher the bar, the strongest the association). The linkage disequilibrium pattern for the CEPH individuals genotyped by the HapMap project is displayed below (using r^2^).

The 83 remaining genes (103 SNPs) with significant population-average allelic imbalance included several genes for which allelic imbalance had been shown in previous studies (e.g. IL1A or IGF1 described in [30]). For some genes (e.g., CHI3L2), one allele/haplotype is clearly expressed more than the other in heterozygotes and the inheritance pattern in families supports a genetic cause for allelic imbalance. For other genes, neither the direction of allelic imbalance nor the pedigree analysis allowed us to easily differentiate the genetic/epigenetic cause of the differential allelic expression (Table 1). For 56 genes with significant differences in allelic expression we tested whether differential allelic expression could be statistically associated to one of the SNP in the vicinity of the gene genotyped by the HapMap project (see Materials & Methods for details). The results of these tests for SERPINB10 and ABCG1 are shown on Figure 7 and the strongest nominal association for each gene is displayed on Table 2. Twenty-three genes still display statistical significant associations after Bonferroni correction for multiple testing (highlighted in green on Table 2) showing a clear enrichment relative to the 2–3 associations expected by chance. Our power to detect a significant association between a HapMap SNP and the under-/over-expressing chromosome in this setting is low due to our reduced sample size (only the heterozygous individuals are taken into account in this analysis) and the number of regulatory haplotypes identified is thus likely underestimated. Additionally, many SNPs are tested for each gene and it is thus possible that some of the regulatory haplotypes result from spurious associations (i.e. they are false positives). One argument against a very high rate of false positive in our analysis is that imprinted genes such as MEST or PEG10 do not show any signal of association (Figure S8) consistent with the fact that the *cis*-regulatory mechanism at these genes is not encoded in the DNA sequence. To further investigate the validity of our association, we attempted to independently confirm these regulatory haplotypes by testing for the statistical association between one SNP in the regulatory haplotype and gene expression level. We used gene expression measurements performed at the Wellcome Trust Sanger Institute (kindly provided by M. Dermitzakis) on the same individual cell lines assayed by Illumina gene expression arrays. For each gene, we tested whether the homozygotes for the regulatory haplotype associated with low allelic expression in heterozygotes show a significantly lower gene expression level than the homozygous individuals for the regulatory haplotype associated with high allelic expression. We also performed locus-specific RT-PCR and quantified the level of gene expression using SYBR-Green for eleven genes for which differential allelic expression was significantly associated with allelic expression but for which expression data were not available (2 genes) or genes with strong association with a regulatory haplotype but that were not validated using the Sanger dataset (9 genes). Overall, out of the 47 genes with a significant association between a SNP (or several, defining the regulatory haplotype) and differential allelic expression at the nominal cut-off, 10 were confirmed using gene expression measurements while 5 other genes showed a trend but did not reach statistical significance (Table 2).

**Table 2:** Results of the association mapping and validation assays.

Gene	Chr	# Hets	Association	Sanger	RT-PCR	Mapped to
ABCG1	21	18	**2.20E-10**	6.81E-01	**5.78E-01**	Intron
ARSA	22	17	**2.26E-05**	NA		Gene
ASCL1	12	13	1.69E-02	**1.37E-02**		
BCR	22	6	6.06E-02	9.83E-01		
BMP6	6	5	4.76E-02	8.62E-01		
C21orf13	21	9	**4.11E-05**	No data	**3.78E-02**	Gene-5′
C21orf7	21	12	3.71E-01	7.06E-01		
C21orf91	21	17	**1.54E-08**	**8.47E-04**		Gene
C22orf8	22	8	**1.55E-04**	No data		Gene
CDH2	18	22	**5.32E-05**	7.00E-01		Gene
CXCL9	4	7	4.66E-03	3.43E-01		Whole Region
CYP1B1	2	17	**2.11E-06**	**1.43E-02**		Gene-5′
DDX43	6	9	3.47E-01	5.56E-01		
EDNRB	13	14	7.03E-03	6.17E-01		Whole Region
EGFR	7	6	4.55E-01	6.21E-01		
F2R	5	10	**1.08E-05**	7.32E-01	**9.65E-02**	3′UTR
FCRL3	1	14	**6.88E-05**	No data	**8.04E-03**	Gene-3′
FGF2	4	22	6.00E-03	7.63E-01		Gene-3′
FLJ35801	22	13	4.72E-02	1.23E-01		
GAS7	17	12	**7.40E-07**	2.96E-01	**1.45E-01**	3′UTR
GNAZ	22	5	7.94E-03	6.42E-01	**1.16E-01**	Gene-3′
IGF1	12	13	2.38E-01	9.17E-01		
IGSF4	11	15	1.70E-03	2.91E-01	2.59E-01	Gene-3′
IL12B	5	13	**1.92E-07**	**4.51E-02**		Whole Region
IL17RB	3	14	**5.98E-06**	6.81E-01	7.33E-01	5′
IL1A	2	9	1.49E-01	8.74E-01		
IMPACT	18	11	7.52E-03	6.63E-01		5′distal ?
ITGB1BP1	2	18	1.58E-03	No data		Gene
JAM2	21	9	2.26E-03	NA		5′ ?
KCNJ15	21	13	1.69E-02	3.25E-01		Gene
KCNQ1	11	21	2.07E-02	1.59E-01		
KLHDC7B	22	21	**3.72E-12**	No data		Gene-3′
LILRP2	19	10	2.30E-02	No data		
LOC284837	21	16	3.73E-02	No data		
MAPK10	4	11	**2.84E-06**	5.25E-01		Gene
MCAM	11	12	6.44E-04	5.21E-01		Gene/3′
MEST	7	9	1.53E-01	5.50E-01		
MGC33648	5	21	**4.40E-06**	**3.59E-05**		5′
MMP7	11	8	4.66E-03	7.46E-01		
MOXD1	6	8	6.99E-03	1.12E-01		5′
NDUFV3	21	11	**2.84E-06**	**3.73E-05**		Gene-3′
PEG10	7	22	4.59E-02	NA		
PLSCR3	17	10	1.98E-02	2.22E-01		
PLXDC2	10	8	7.69E-02	**3.40E-02**		
PTGS2	1	5	2.06E-01	NA		
PXN	12	14	**4.23E-04**	9.24E-01		Gene-3′
RBM11	21	10	**1.08E-05**	4.59E-01	**1.71E-01**	3′
RIBC2	22	12	**7.40E-07**	No data		Gene
SERPINB10	18	22	**1.84E-05**	**5.98E-11**		5′
SERPINB2	18	10	**1.19E-04**	2.98E-01		Gene-5′
SNRPN	15	27	3.94E-02	3.78E-01		
TMPRSS6	22	12	**7.40E-07**	8.42E-01		3′UTR
TRIM6	11	8	2.56E-02	9.84E-01		
TSGA2	21	8	1.55E-04	9.81E-01		Gene
WNT2	7	9	9.05E-03	3.60E-01	6.45E-01	5′ Gene
ZNF215	11	20	3.73E-02	**1.45E-03**	**1.82E-02**	Gene-5′

#Hets: number of heterozygous individuals for the gene considered among the HapMap individuals analyzed.

Association: lowest nominal p-value obtained by Fischer exact test of the association of the alleles of all SNPs with over-/under-expressing chromosomes. Associations remaining significant after Bonferroni correction for multiple testing are highlighted in green.

Sanger: p-value obtained in the linear regression of the Sanger gene expression measurements with the alleles of the SNP most strongly associated with allelic expression.

RT-PCR: p-value obtained in the linear regression of the locus-specific RT-PCR gene expression measurements with the alleles of the SNP most strongly associated with allelic expression.

Mapped to: broad localization of the regulatory haplotype with regards to the gene with significant difference in allele expression.

## Discussion

### Biology, Not Experimental Noise, Is Responsible for Differential Allelic Expression

We analyzed differences in relative allelic expression (or allelic imbalance) at 1,380 human genes using 2,968 SNPs and more than 80 lymphoblastoid cell lines from individuals with European ancestry. Using quantitative sequencing we validated our results for a subset of genes and showed that the experimental variability in both settings is low and that the Illumina ASE assay and quantitative sequencing of RT-PCR products yield reproducible estimates of allelic imbalance consistent with each other. Overall, the experimental noise is much lower than the difference in relative allelic expression observed at many loci and therefore cannot be responsible for it. Additionally, the high concordance of the results obtained using different SNPs in the same transcript supports our findings that allelic imbalance, as we estimated it, is not an experimental artefact but reflects inherent biological differences in the relative expression of both alleles in heterozygous individuals. We also showed that lymphoblastoid cell lines, despite being simplified biological materials, are suitable resources to investigate mechanisms of gene regulation. Here, we demonstrated that our estimation of allelic imbalance is little affected by growth conditions and that LCLs harvested from different passages yield very similar results. Finally, the results efficiently recapitulate the consequences of the epigenetic mechanisms established in the individuals from which the cells have been derived (see also [43]). We are therefore confident that, overall, the patterns of allelic imbalance we observed are neither experimental artifacts, nor specific to the material studied, but represent a common biological phenomenon affecting human gene expression.

### Differential Allelic Expression Identifies Consequences of Epigenetic Mechanisms of Gene Regulation

We showed that LCLs derived from female individuals still harbor the consequences of X-inactivation at all X-linked genes investigated, with one allele being transcriptionally silenced [34]. The extent of allelic imbalance detected at X-linked genes can vary among LCLs due to the various degrees of clonality of these cells but clonal LCLs consistently show complete silencing of one allele at all X-linked genes investigated (Figure S7). In addition, imprinting, established in the germ lines of the parents of the individuals from which the cells are derived [44], is also maintained in LCLs. In our experiments, PEG10, SNPRN, MEST and KCNQ1 show reduced or absent expression of one allele and, when the mode of inheritance can be determined, it corresponds to the imprinting mechanism described in the literature (i.e. PEG10 and SNPRN are maternally imprinted, KCNQ1 is paternally imprinted). We thus observe extensive differential allelic expression (i.e. allelic ratio larger than 70∶30) for all genes whose expression is known to be epigenetically regulated. This clearly shows that analysis of differential allelic expression is a suitable method for identifying the consequences of epigenetic mechanisms of gene regulation. The Illumina ASE assay would thus provide an efficient method to screen tumor tissues and identify patterns of differential allelic expression resulting from aberrant methylation or loss of imprinting that are known to be involved in the etiology of cancers [45]–[47].

Interestingly, IMPACT which shows significant extent of allelic imbalance at two SNPs (rs677688 and rs1053474) in our study, is known to be imprinted in mice [48] but not in humans [49]. The mode of inheritance of the over-expressed alleles could not be determined using the two families available in our study (i.e. the parents were always homozygous for the same allele). The attempt to map differential expression to a regulatory haplotype was not successful and is consistent with an epigenetic mechanism of gene regulation. More investigations are required to determine whether the pattern of allelic imbalance observed for IMPACT results from incomplete silencing of one allele following imprinting in the parental germ-lines or whether it results from random mono-allelic expression or another mechanism of gene expression regulation.

### Regulatory Polymorphisms Determine Allelic Expression for Some Human Genes

Our analysis of 643 genes expressed in LCLs shows that, for a large proportion of them (∼20%), the two alleles are differentially expressed in most heterozygous individuals. For 18 genes, differential expression resulted from a known epigenetic silencing of one of the two alleles, either through X-inactivation in females or imprinting. The mechanisms leading to allele-specific expression at all other genes could be driven by a polymorphism affecting the cis-acting regulation (e.g. a SNP in a transcription factor or a miRNA biding site) or simply result from random silencing of one of the two alleles. We tested 56 genes for association of differential allelic expression patterns observed with a *cis*-acting regulatory polymorphism using genotypes generated by the HapMap project (see Materials and Methods for details). For 23 of these genes we identified a region statistically associated with differences in allele expression that could indicate the existence of a regulatory haplotype (i.e., a region of one chromosome likely containing the polymorphism(s) causing the differential *cis*-regulation). These regions are often tens of kb long, consistent with previous descriptions of the linkage disequilibrium patterns in humans [50]. Although this approach does not identify the actual polymorphism(s) responsible for the differential *cis*-regulation, examination of these regulatory haplotypes provides some valuable insights on the mechanisms leading to differential expression and can guide future investigations. For example, the regulatory haplotype for GAS7 is almost exclusively restricted to the 3′UTR of the gene and may indicate that the patterns of allelic imbalance observed are due to differential mRNA processing, stability or the presence of a 3′ enhancer. In contrast, the regulatory haplotype identified for MGC33648 is located in the 5′ region and does not seem to overlap with the gene itself. This might be indicative of alternative promoter usage or differential transcription efficiency (e.g. due to differential transcription factor binding site affinity).

### Allelic Imbalance Is Complementary of Total Gene Expression Association

Several recent studies have used large-scale associations between gene expression and extensive genotype information to investigate gene regulation in humans, some of them using cell lines included in our study. In particular, Stranger and colleagues analyzed 630 genes located in ENCODE regions, on chromosome 21 and in one portion of chromosome 20. They found evidence of *cis*-acting regulation for 63 genes [19]. 2005). We were able to analyze 21 of these genes in our experiment. Six of them also showed evidence of *cis*-acting regulation (e.g. SERPINB10 or TSGA2) in our study while a seventh gene (TTC3) showed patterns consistent with differential allelic expression but did not reach our significance threshold. The remaining 14 genes did not show evidence of differential allelic expression in our analysis. Alternatively, we identified 10 new genes located in ENCODE region or chromosome 21 that showed significant level of differential allelic expression but were not detected in the Stranger study. Several non-exclusive reasons could explain the discrepancies between the results of the two approaches. First, it is worth noting that, even if the same individuals are analyzed by allelic-specific expression and gene expression association, the power to detect *cis*-acting effect differs depending on the allele frequency of the marker used: in gene expression association analysis all individuals are analyzed but the power in the regression analysis depends on their genotypes (e.g. the genotypes AA, AB and BB are encoded in the linear regression as 0, 1 and 2) while in allelic expression analysis only the individuals heterozygotes at the marker considered are analyzed. This can become particularly problematic to study differential allelic expression at some genes since it requires a relatively common exonic SNP to detect allelic imbalance. In this context, it is worth noting that intronic SNPs can successfully be used for genes that are highly expressed (see also [30]). Second, associations of gene expression to genotypes depends greatly on the linkage disequilibrium (LD) patterns and requires extensive genotype information from all the individuals in order to include one marker in LD with the regulatory polymorphism. Allelic expression, on the other hand, directly investigate *cis* effect directly at the gene level and thus only requires physical link between the gene and the regulatory polymorphism affecting it (i.e. they need to be on the same chromosome). Finally, the differences between allelic expression and gene expression mapping might indicate that some genes are also regulated by *trans*-acting mechanisms that differ among individuals: differential allelic expression is influenced only by *cis*-acting mechanisms of gene regulation while gene expression is influenced by *cis*- and *trans*-acting gene regulation. It is thus not unlikely that individual differences in *trans*-acting regulation swamp the signal from *cis*-acting polymorphisms. In this context, it is noteworthy that total gene expression mapping has been much more successful in mice and yeast for which the genetic heterogeneity is much lower and can be controlled (reviewed in [9],[10],[51]). In humans, or in any other outbred population, genetic heterogeneity greatly limits the identification of *cis*-acting mechanisms using gene expression data while measurements of differential allelic expression are unaffected.

We showed here that allelic expression assays are complementary from gene expression mapping and that the Illumina ASE assay overcomes two of the major limitations and criticisms of the former methodologies used to assess differential allelic expression: it allows a robust and high-throughput estimation of allelic imbalance: it is now possible to reliably screen hundreds of RNAs for several hundreds of genes in a couple of days. Additionally, when several SNPs can be used to assess differential allelic expression, the assay becomes very robust since each marker provides an independent estimation and one can test the correlation among estimates obtained at different positions. It is worth noting here that since this assay relies on the comparison of allelic ratio in DNA and RNA of each individual, it internally controls for the existence of polymorphisms in the primer sites or copy number variation encompassing the gene studied (that will affect equally DNA and RNA). Likely, the greatest advantage of the analysis of differential allelic expression over total gene expression is its flexibility. To identify differential regulation of gene expression using total gene expression, one needs extensive genotype information to test whether, at any polymorphic position, the gene expression differences among individuals segregate according to their genotype. This precludes a quick assessment of the expression of one locus in one cohort of particular interest or using a specific tissue. In contrary, differential allelic expression offers the advantage that any one gene can be quickly assessed in any cohort or tissue by simply comparing the expression of the two alleles in each individual (the amount of genetic information recently made available by the HapMap project allows a quick and easy selection of markers likely to be polymorphic for a given gene). The determination of regulatory haplotypes would still require extensive information concerning surrounding polymorphisms but the initial screening to determine whether one transcript is differentially *cis*-regulated can be done very efficiently with a handful of markers.

### Conclusion

We showed that differential allelic expression is a robust approach to identify *cis*-acting mechanism of gene regulation. It complements gene expression association studies and offers additional perspectives, notably on epigenetic mechanisms of gene regulation. It could thus be particularly interesting to apply this assay to tumors to detect mis-regulated genes due to aberrant methylation patterns or loss of imprinting. In addition, our approach is applicable to any new cohort or tissue since it is self-sufficient to identify differential *cis*-regulation and does not require additional genotyping. It can be easily used to follow-up interesting non-coding regions associated to a particular disease and test if they are involved in the etiology of the disease through some regulatory effects on neighboring genes.

## Materials and Methods

### Sample Preparation

83 lymphoblastoid cell lines (LCL) derived from blood samples from the CEPH collection were selected for this project. They included 60 unrelated individuals obtained from Utah residents with ancestry from western and northern Europe for which DNA was genotyped for millions of SNPs covering the entire genome by the International HapMap Project. Additionally, 21 LCLs from CEPH pedigrees 1420 and 1444 were included to provide complete information on two three-generation CEPH families. Cells were grown at 37°C and 5% CO_2_ in RPMI 1640 medium (Invitrogen, Burlington, Canada) supplemented with 15% heat-inactivated fetal bovine serum (Sigma-Aldricht, Oakville, Canda), 2 mM L-glutamine (Invitrogen, Burlington, Canada) and penicillin/streptomycin (Invitrogen, Burlington, Canada). The cell growth was monitored with a hemocytometer and the cells were harvested when the density reached 0.8–1.1 × 10^6^ cells/mL. Cells were then resuspended and lysed in TRIzol reagent (Invitrogen, Burlington, Canada). For all LCLs, three successive growths were performed (corresponding to the 2^nd^, 4^th^ and 6^th^ passages) after thawing frozen cell aliquots.

### Illumina Allele-Specific Expression (ASE) Assay

We estimated allelic imbalance at 1,380 genes (two panels of ∼1,500 SNPs, Figure 1) using the Illumina ASE assay (Figure 2). The experiment is similar to the one used for large-scale SNP genotyping [52] and gene expression profiling [53] except that DNA and RNA are independently assessed and compared to each other. RNA was first converted into biotinylated cDNA [53] while DNA was treated according to the usual GoldenGate assay protocol [52]. Biotinylated DNA (derived from genomic DNA or mRNA) was immobilized on paramagnetic beads and pooled SNP-specific oligonucleotides were annealed on the DNA. Hybridized oligonucleotides were then extended and ligated to generate DNA templates, which were amplified using universal fluorescently-labeled primers. Finally, single-stranded PCR products were hybridized to a Sentrix Array Matrix [52], and the arrays were imaged using the BeadArray Reader Scanner [54]. 96 samples (DNA or RNA) were analyzed per Sentrix Array for ∼1,500 SNPs. All RNA measurements were performed in duplicates.

### Analyses of ASE Results

To estimate the extent of allelic imbalance in heterozygote individuals at each SNP of the Illumina ASE panel, we developed algorithms using two different approaches: i) we used information from individuals of all three genotypes (AA, AB and BB), and/or ii) we used only the heterozygote individuals.

We first determined whether a given gene was expressed above a determined background in a given individual. To do so, we made use of the fact that the genotypes were known (from the DNA analysis) and developed a locus-specific expression background cut-off: homozygote individuals (i.e. AA or BB) can only express the corresponding allele, respectively A or B, at the RNA level (if at all). We thus determined a background fluorescence level (i.e. corresponding to random noise) for each allele (i.e. A and B) by measuring the emission in the corresponding dye (respectively, Cy3 and Cy5) in individuals homozygous for the other allele (respectively BB and AA). This is represented schematically on Figure S9. To avoid false positive results due to the inclusion of transcripts not expressed in the cell lines considered, we used a conservative approach and arbitrarily fixed the background emission cutoff to the maximum emission of the absent allele of all homozygotes, plus the mean emission of the absent allele divided by the number of homozygotes (to weight the uncertainty in the determination of the “maximum noise” by the numbers of individuals used to determine it). This procedure allowed us to independently estimate the background emission of each allele/dye specifically for each SNP, which is particularly important because the fluorescence emission can differ drastically between the dyes and among loci (data not shown). We then proceeded to the detection call using the background cut-offs: individuals with genotypes AA were considered to express a given transcript if the emission was larger than twice the cutoff background emission of A, individuals with genotypes AB if the fluorescence was larger than the sum of the background emission of A and the background emission of B, and individuals with genotypes BB if the emission is larger than twice the background emission of B. Since the inclusion in the analyses of transcripts expressed at low level (or not expressed at all) is very problematic, we excluded from our analyses all loci for which less than 75% of the individuals had discordant replicate expression (i.e., one replicate above expression background, the other under the cut-off value).

The first method used to determine whether some heterozygote individuals expressed significantly differently the two alleles is locus-specific but requires having at least one individual expressed from each homozygote genotype (AA and BB). In this case, we determined the median log ratio of the two dyes for each homozygote clusters at the DNA and RNA level (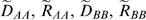) as well as the median absolute deviations (MAD). We used medians and MADs, instead of means and standard deviations, to down weight the influence of possible outliers. We then determined a range of “expected” (i.e. non significant) variation of allelic expression for the heterozygote individuals. We calculated the equation of the lines joining the median values plus/minus two MAD of AA and BB and estimated the range, for the log ratio of the dyes at the RNA level, between the lines at the value corresponding to the median of DNA in heterozygote individuals (Figure S10). If the observed log ratio of dyes for a given heterozygote individual fell outside the expected range of variation in absence of AI (Figure S8), we scored each heterozygote individual separately to obtain a quantitative estimation of allelic imbalance using the ratio:
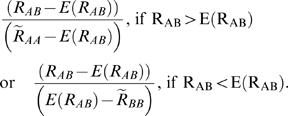
This simple estimate indicates both the magnitude of the allelic imbalance (i.e. the fold difference) and its direction (i.e. which allele is more expressed than its counterpart).

In order to assess allelic imbalance for SNPs with low minor allele frequencies (for which homozygote individuals with the minor allele may not be present in a small sample size panel), we developed a second method based solely on the heterozygote individuals. If a given transcript is affected by allelic imbalance we expect that either the variance of the log ratio of dyes for heterozygote RNAs to be greatly increased relative to the variance of homozygote RNAs, or, if one allele is systematically more expressed than the other, the mean value of these log ratios to be drastically shifted from its expected intermediate position (between the mean for AA and the mean for BB homozygote RNAs). For all SNPs with at least five individuals with the same genotype expressed, we estimated the standard deviation of the log ratio of dyes for DNA and RNA. The distribution of the log ratio of the standard deviations (i.e. log σ_DNA_/σ_RNA_) over all loci for heterozygous individuals differed from those observed using homozygous individuals and did not seem to fit a normal distribution (Figure S11). Based on the assumption that this distribution may include some loci in allelic imbalance (and thus with a higher than expected RNA variance), we fitted a mixture of two Gaussians on our dataset (i.e., one corresponding to the loci with allelic imbalance, the second including all other loci) using a Maximum Expectation algorithm implemented in R (mixdist package). For our data, the best fit was obtained with a minor distribution (including ∼3% of the loci) corresponding to the most extremely negative log ratios of variances (i.e., that the RNA standard deviation was larger than expected). For each locus, we then used the probability of belonging to the “higher-than-expected RNA variance” distribution as an indication of allelic imbalance.

### Quantitative Sequencing of RT-PCR Products

We assessed the extent of allelic imbalance by quantitative sequencing following the method described in Ge et al. [33]. Briefly, we isolated RNA using TRIzol reagent following the manufacturer's instructions. We assessed RNA quality with an Agilent 2100 Bioanalyzer (Agilent, Palo Alto, USA) before synthesizing first strand cDNA using random hexamers (Invitrogen, Burlington, Canada) and Superscript II reverse transcriptase (Invitrogen, Burlington, Canada). For each locus, we designed locus-specific primers, in the exon/UTR containing the SNP analyzed, at least 50 bp away from the SNP studied. 5 ng of genomic DNA and 10 ng of total cDNA were then amplified by PCR using Hot Start Taq Polymerase (Qiagen, Mississauga, Canada) with an activation step (95°C for 15 minutes) followed by 40 cycles (95°C for 30 s, 55°C for 30 s and 72°C for 45 s) and a final extension step (72°C for 6 minutes). PCR products were purified using Exonuclease I and Shrimp Alkaline Phosphatase (USB, Cleveland, USA) and sequenced using either one of the former primers or a nested primer, on an Applied Biosystems 3730xl DNA analyzer. We used PeakPeaker v.2.0 [33] with the default settings to quantify the relative amount of the two alleles measured from the chromatogram after peak intensity normalization. To estimate the experimental variability of the entire experimental setup we used a hierarchical strategy for two genes (cf. Figure S12): for two/three individual cell lines, we extracted independently RNAs three times and performed, on each extract, three independent RT-PCRs. All cDNA obtained were then split into three aliquots, each amplified independently by locus-specific PCR. These PCR products were finally sequenced each three times (i.e. three independent sequencing reactions). To estimate the variability at each experimental stage we calculated the mean standard variation normalized to the mean using the independent triplicates. To calculate the variance in the higher hierarchical levels (PCR, RT-PCR), we averaged the values from the lower level (e.g., to estimate the variability at the PCR level, we compared the means of the three sequencing values performed on each of the three PCRs: [s1,s2,s3] vs [s4,s5,s6] vs [s7,s8,s9]). The results are presented in Text S1.

### Association Mapping of Differential Allelic Expression

We attempted to map allelic imbalance to regulatory haplotypes for all genes with significant differences in allelic expression that fulfilled these criteria: i) they are mapped on the build 34 of the human genome, ii) the SNP used in the Illumina ASE assay has also been genotyped by the HapMap [55] and iii) there are more than four HapMap individuals heterozygous at the marker SNP. For each gene, we retrieved the haplotype information from the phased chromosomes of each of the 57 HapMap CEPH individuals for 100,000 bp upstream and downstream of the SNP used to assess allelic imbalance. When a transcript contains more than one SNP or if two SNPs used to assess allelic imbalance at two transcripts are separated by less than 200,000 bp, the region retrieved spans from the most upstream marker plus 100,000 bp to the most downstream marker minus 100,000 bp. For each individual LCL, the over expressed and under expressed haplotype/chromosome were identified and each SNP was tested for segregation of the alleles in under- and over-expressed chromosomes using a Fischer's exact test. Between 47 and 592 SNPs were tested for each gene (mean  =  229) and the associations remaining significant after Bonferroni correction for multiple testing are shown in green in Table 2.

### Validation of Regulatory Haplotypes

Illumina total gene expression data were obtained from the Wellcome Trust Sanger Institute for the 60 unrelated CEPH individuals genotyped by the HapMap project and included in our assay. We also determined the total expression for 10 genes using Real-Time PCR and SYBR Green labeling on an ABI 7900HT (Applied Biosystems, Foster City, CA) instrument. 8–10 ng of first strand cDNA were amplified using 0.32 µM of gene specific primers and Power SYBR Green PCR master mix (Applied Biosystems) according to the manufacturer's instructions. The amplifications started by 95°C for 10 min followed by 40 cycles at 95°C for 20 s, 58°C for 30 s and 72°C for 45 s. We performed the Real-Time PCR assays for the 60 individuals LCLs genotyped by the HapMap projects and analyzed 6 replicates per each sample. A standard curve was established using a dilution series of total cDNA of known concentration. The C_t_ for each replicate was transformed to a relative concentration using the estimated standard curve function (SDS 2.1, Applied Biosystems) and normalized based on 18S rRNA *Taq*man (Applied Biosystems) expression data obtained for each sample to account for well to well variability.

### Software

All analysis scripts are available upon request. PeakPicker v.2.0 is available at http://www.genomequebec.mcgill.ca/EST-HapMap/.

## Supporting Information

Text S1 Experimental variability using quantitative sequencing of RT-PCR products.
(0.03 MB DOC)

Figure S1 Correlation between the estimates of allelic expression and the proportions of total RNA extract mixed. The graph displays the p-values of the linear regressions between the allelic ratios and the proportions of mixed RNA. Mixes homozygous-homozygous are shown in red, mixes heterozygous-homozygous are in blue.
(1.94 MB TIF)

Figure S2 Estimation of experimental variability in the Illumina ASE assay. Average difference between duplicates for 411 SNPs analyzed using the Illumina ASE Cancer Panel. The variability is shown for each SNP as the fraction of the difference between the median dye ratio for homozygotes for one allele and the median dye ratio for homozygotes for other allele (e.g., a variability of 0.1 could artificially generate an allelic ratio of 60∶40 in heterozygotes).
(1.86 MB TIF)

Figure S3 Assessment of differential allelic expression using quantitative sequencing of RT-PCR products. First strand cDNA is synthesized from total RNA extract using random hexamers and amplified by locus-specific primers surrounding a particular coding SNP. The allelic ratio is estimated directly from the sequencing trace file with the software PeakPicker v2.0.
(3.26 MB TIF)

Figure S4 Influence of the culture conditions. The figure shows the correlation between the estimates of allelic imbalance using quantitative sequencing for cells harvested after 4 (“Harvest 2”, x-axis) and 6 (“Harvest 3”, y-axis) passages. Each blue cross stands for one heterozygous individual for the gene IGF1 (A), IL1A (B) and CHI3L2 (C).
(2.50 MB TIF)

Figure S5 Exonic vs. intronic SNP. The graph shows the average number of individuals expressing a detectable transcript using an exonic SNP or an intronic SNP.
(1.95 MB TIF)

Figure S6 Population-average estimates of allelic imbalance at 777 SNPs (both panels combined). See legend of Figure 4.
(1.93 MB TIF)

Figure S7 Clonality and X-linked genes. The allelic imbalance estimates for 11 X-linked SNPs (in 7 genes) are displayed on the y-axis for every female individual (x-axis) (if the individual is heterozygous at the position considered).
(2.50 MB TIF)

Figure S8 Association mapping of allelic imbalance to regulatory haplotypes for MEST (A) and PEG10 (B).
(4.54 MB TIF)

Figure S9 Method used for the detection of transcript expression. See Materials and Methods for details.
(1.86 MB TIF)

Figure S10 Individual assessment of differential allelic expression on the Illumina ASE assay. See Materials and Methods for details.
(1.93 MB TIF)

Figure S11 Variance-based assessment of differential allelic expression on the Illumina ASE assay. See Materials and Methods for details.
(1.91 MB TIF)

Figure S12 Estimation of experimental variability in quantitative sequencing assay. We performed, for two genes (and five individuals), triplicates of each experimental step: from one cell harvest we extract RNA three times independently. Each extract was then subject to three independent RT-PCRs and each aliquot was amplified three times by locus-specific PCR. Finally, PCR products were sequenced three times and allelic imbalance estimated using PeakPicker v2.0.
(1.54 MB TIF)

Table S1 List of the 2,968 SNPs analyzed using the Illumina ASE assay.
Origin. Displays if the gene is located in a ENCODE region, on chromosome 21 or 22 and whether the genes was included for its potential involvement in disease etiology.
Intron/exon. SNPs in 3′UTR are shown as “exon”.
(0.09 MB PDF)

Table S2 All SNPs expressed in at least three heterozygous individuals
(0.05 MB PDF)
