# Supplementary material for: Differential Allelic Expression in the Human Genome: A Robust Approach To Identify Genetic and Epigenetic Cis-Acting Mechanisms Regulating Gene Expression
Source: PLoS Genet. 2008 Feb 29;4(2):e1000006. doi: 10.1371/journal.pgen.1000006 (PMC2265535; doi:10.1371/journal.pgen.1000006)
Supplement: Text S1 — Experimental variability using quantitative sequencing of RT-PCR products. (0.03 MB DOC) [file pgen.1000006.s001.doc]

**Experimental variability using quantitative sequencing of RT-PCR products.**

We used a hierarchical strategy to investigate in details the experimental noise introduced at each step: we performed triplicates of each experiment in order to determine its variability using two genes and two or three heterozygous individuals see Supplemental Figure S9). Most of the experimental variability comes from the locus-specific PCR and could be due to low amount of cDNA templates introduced in the reaction (since we typically amplify 10 ng of total cDNA regardless of the abundance of the gene transcript being studied). All other experimental steps are strikingly robust and show very little variability among replicates. Overall, the cumulated experimental variability is low (~10%, corresponding to a maximum 1.2-fold difference in allelic expression for heterozygote individuals or an allelic ratio of 55:45) with regards to the intensities of allelic imbalance, as reported in previous studies using this methodology (Ge et al. 2005; Pastinen et al. 2005).
