# Supplementary figures and images for: Differential Allelic Expression in the Human Genome: A Robust Approach To Identify Genetic and Epigenetic Cis-Acting Mechanisms Regulating Gene Expression
Source: PLoS Genet. 2008 Feb 29;4(2):e1000006. doi: 10.1371/journal.pgen.1000006 (PMC2265535; doi:10.1371/journal.pgen.1000006)

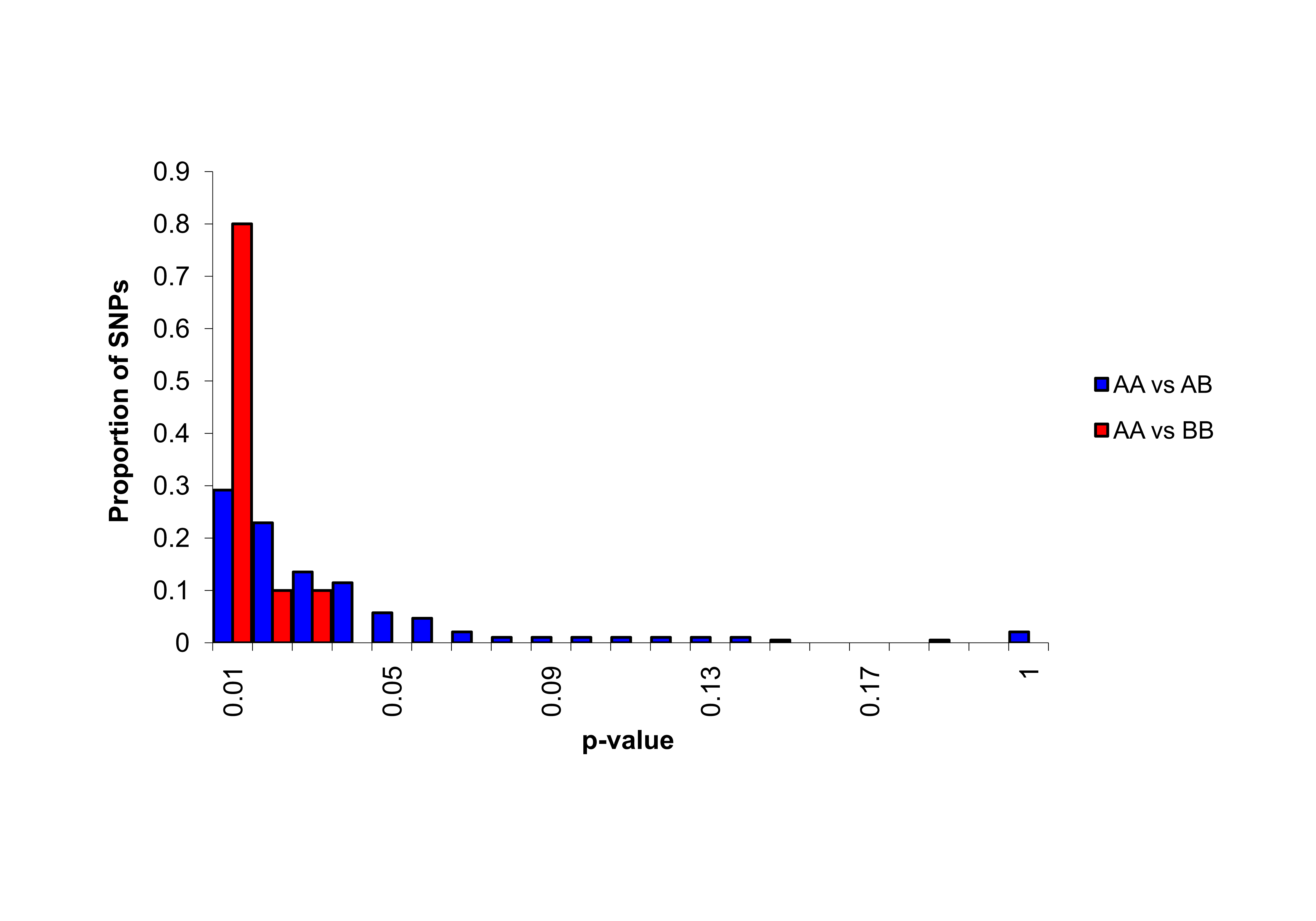

Supplement: Figure S1 — Correlation between the estimates of allelic expression and the proportions of total RNA extract mixed. The graph displays the p-values of the linear regressions between the allelic ratios and the proportions of mixed RNA. Mixes homozygous-homozygous are shown in red, mixes heterozygous-homozygous are in blue. (1.94 MB TIF) [file pgen.1000006.s002.tif]

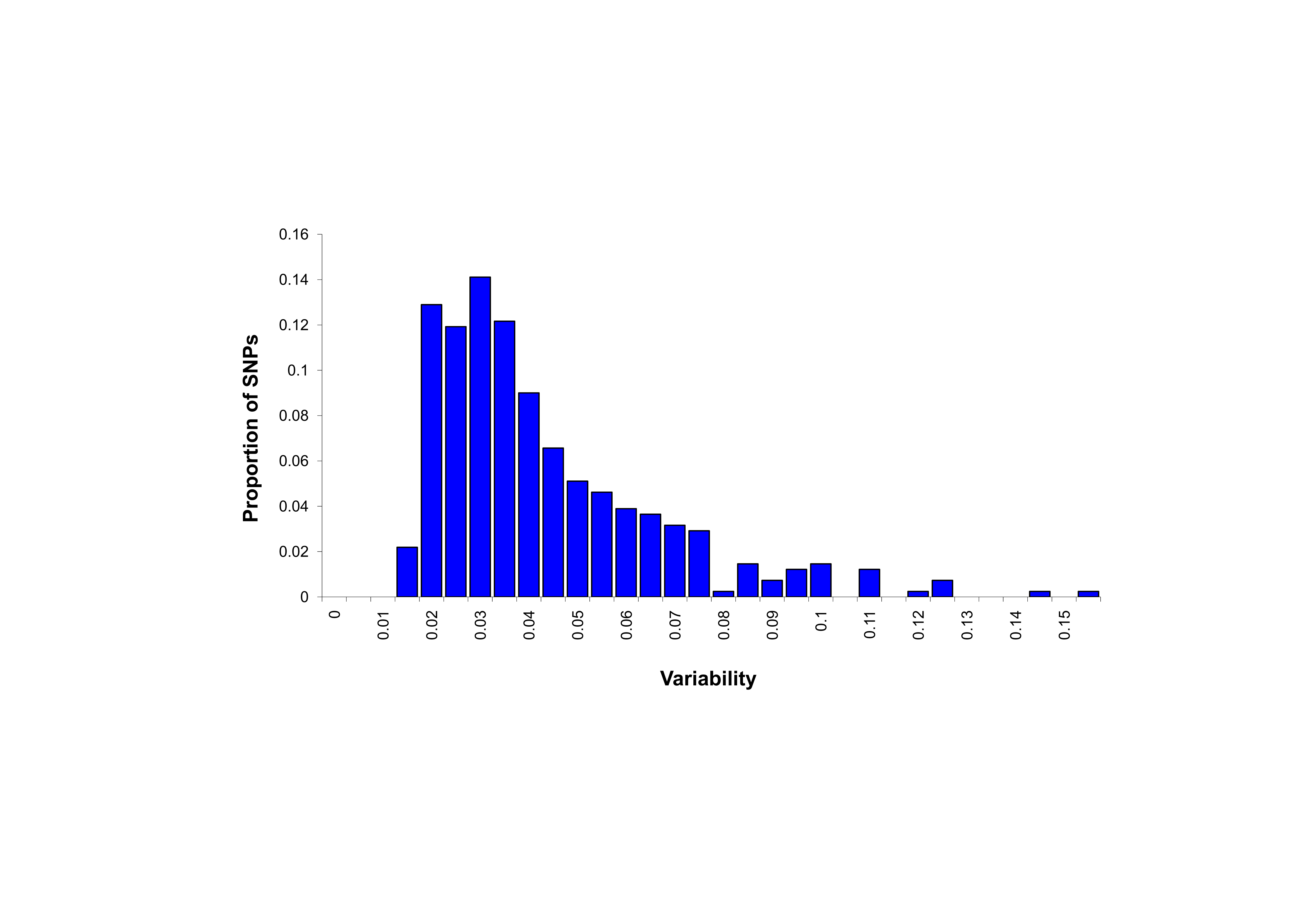

Supplement: Figure S2 — Estimation of experimental variability in the Illumina ASE assay. Average difference between duplicates for 411 SNPs analyzed using the Illumina ASE Cancer Panel. The variability is shown for each SNP as the fraction of the difference between the median dye ratio for homozygotes for one allele and the median dye ratio for homozygotes for other allele (e.g., a variability of 0.1 could artificially generate an allelic ratio of 60∶40 in heterozygotes). (1.86 MB TIF) [file pgen.1000006.s003.tif]

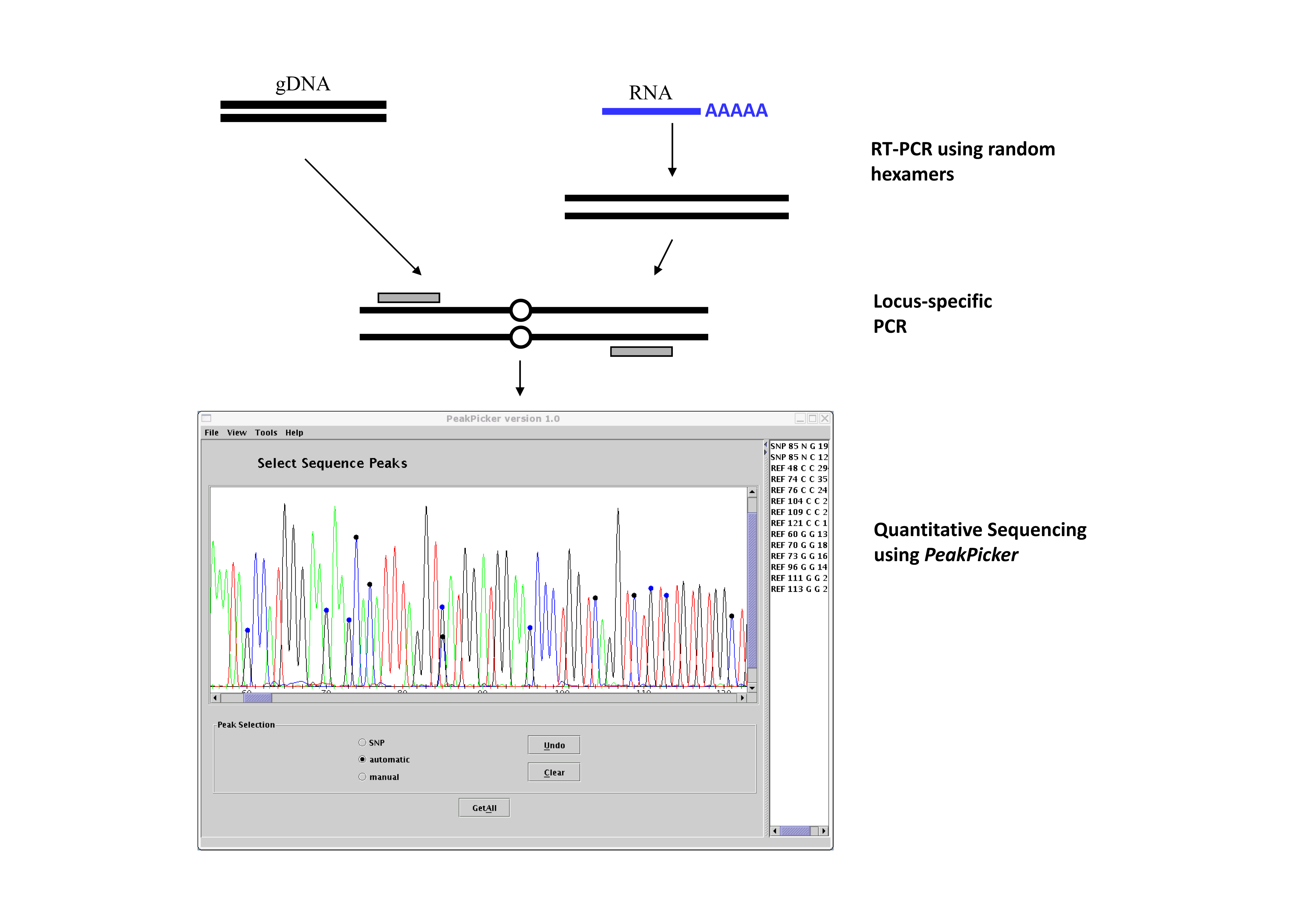

Supplement: Figure S3 — Assessment of differential allelic expression using quantitative sequencing of RT-PCR products. First strand cDNA is synthesized from total RNA extract using random hexamers and amplified by locus-specific primers surrounding a particular coding SNP. The allelic ratio is estimated directly from the sequencing trace file with the software PeakPicker v2.0. (3.26 MB TIF) [file pgen.1000006.s004.tif]

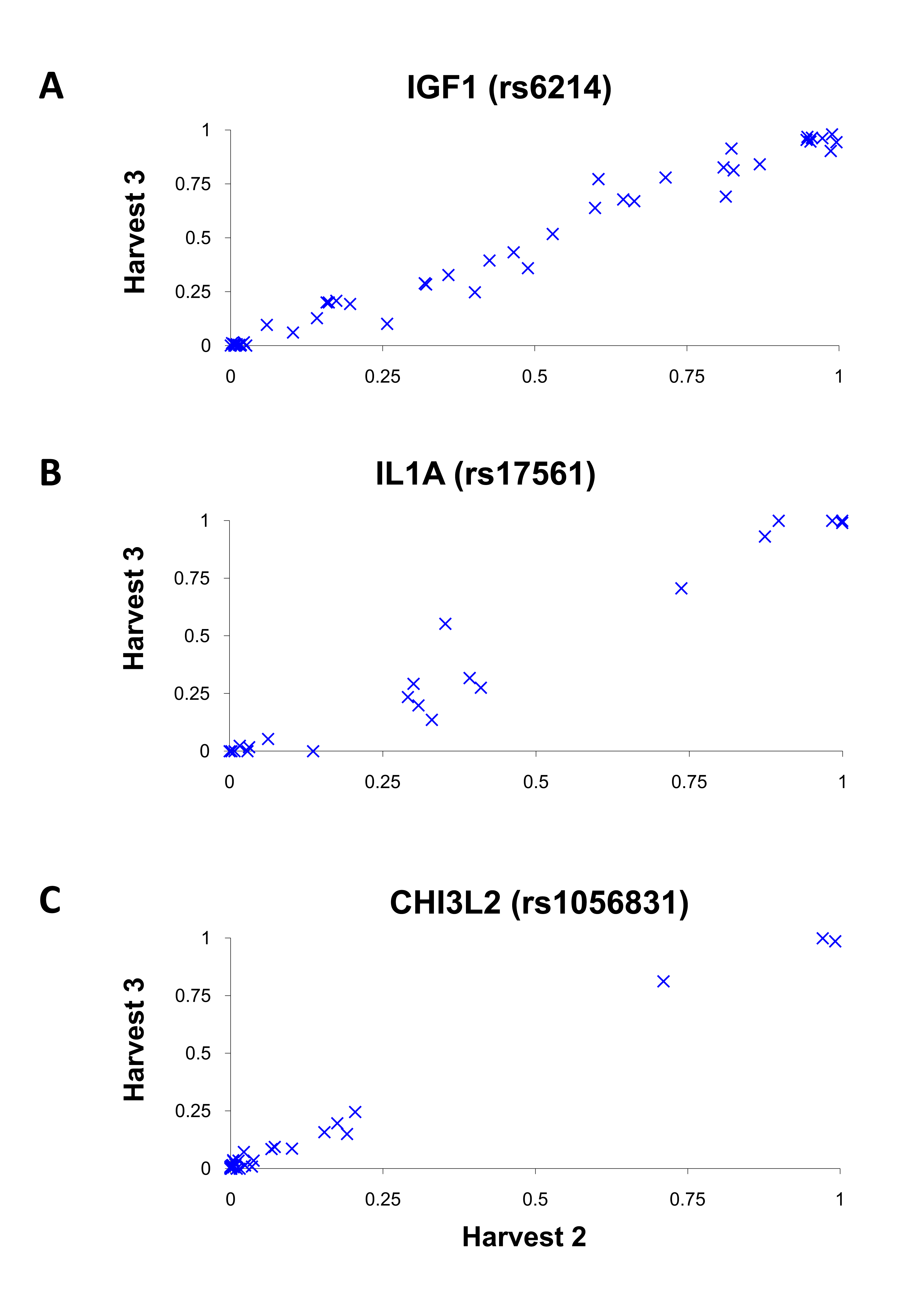

Supplement: Figure S4 — Influence of the culture conditions. The figure shows the correlation between the estimates of allelic imbalance using quantitative sequencing for cells harvested after 4 (“Harvest 2”, x-axis) and 6 (“Harvest 3”, y-axis) passages. Each blue cross stands for one heterozygous individual for the gene IGF1 (A), IL1A (B) and CHI3L2 (C). (2.50 MB TIF) [file pgen.1000006.s005.tif]

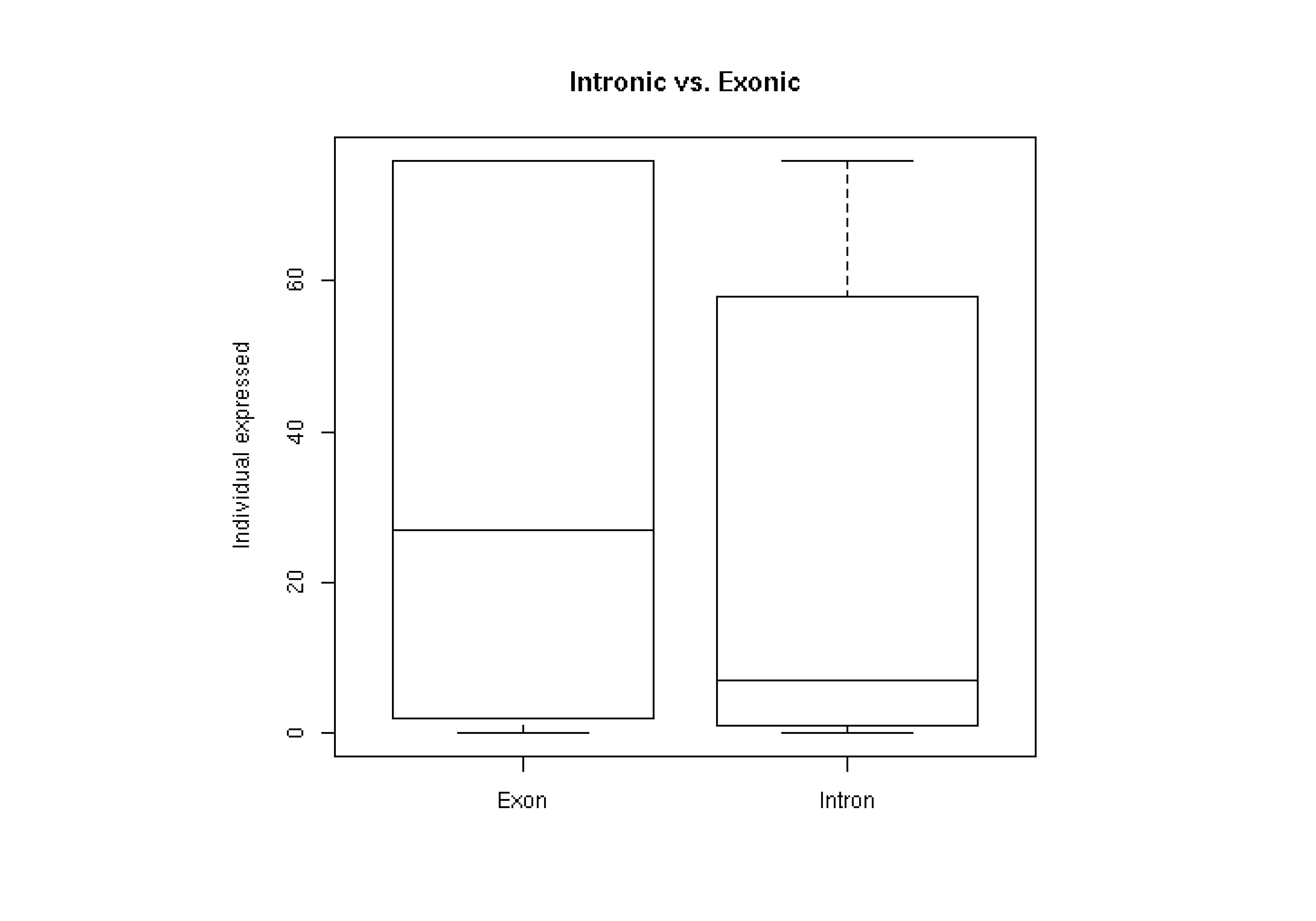

Supplement: Figure S5 — Exonic vs. intronic SNP. The graph shows the average number of individuals expressing a detectable transcript using an exonic SNP or an intronic SNP. (1.95 MB TIF) [file pgen.1000006.s006.tif]

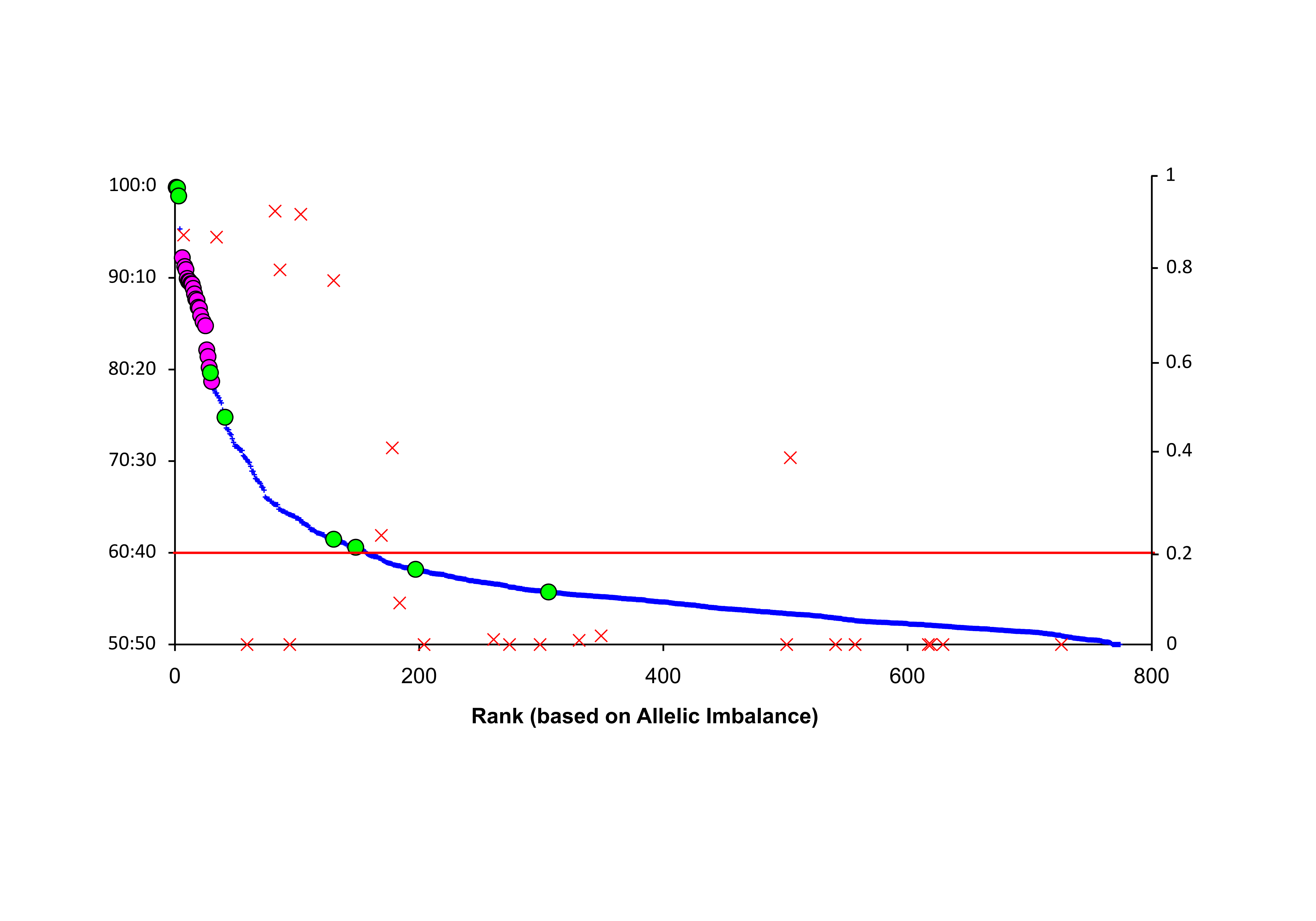

Supplement: Figure S6 — Population-average estimates of allelic imbalance at 777 SNPs (both panels combined). See legend of Figure 4. (1.93 MB TIF) [file pgen.1000006.s007.tif]

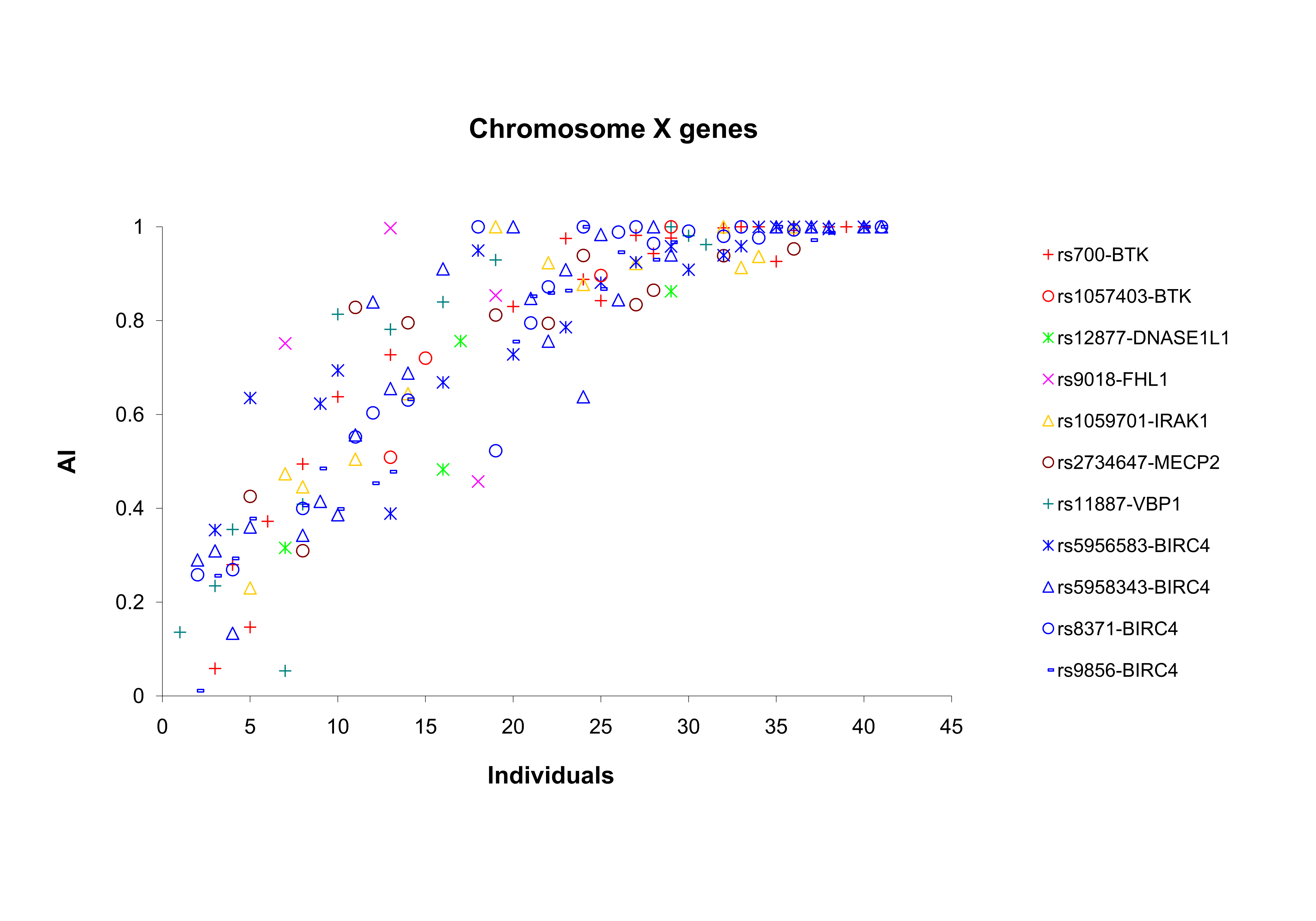

Supplement: Figure S7 — Clonality and X-linked genes. The allelic imbalance estimates for 11 X-linked SNPs (in 7 genes) are displayed on the y-axis for every female individual (x-axis) (if the individual is heterozygous at the position considered). (2.50 MB TIF) [file pgen.1000006.s008.tif]

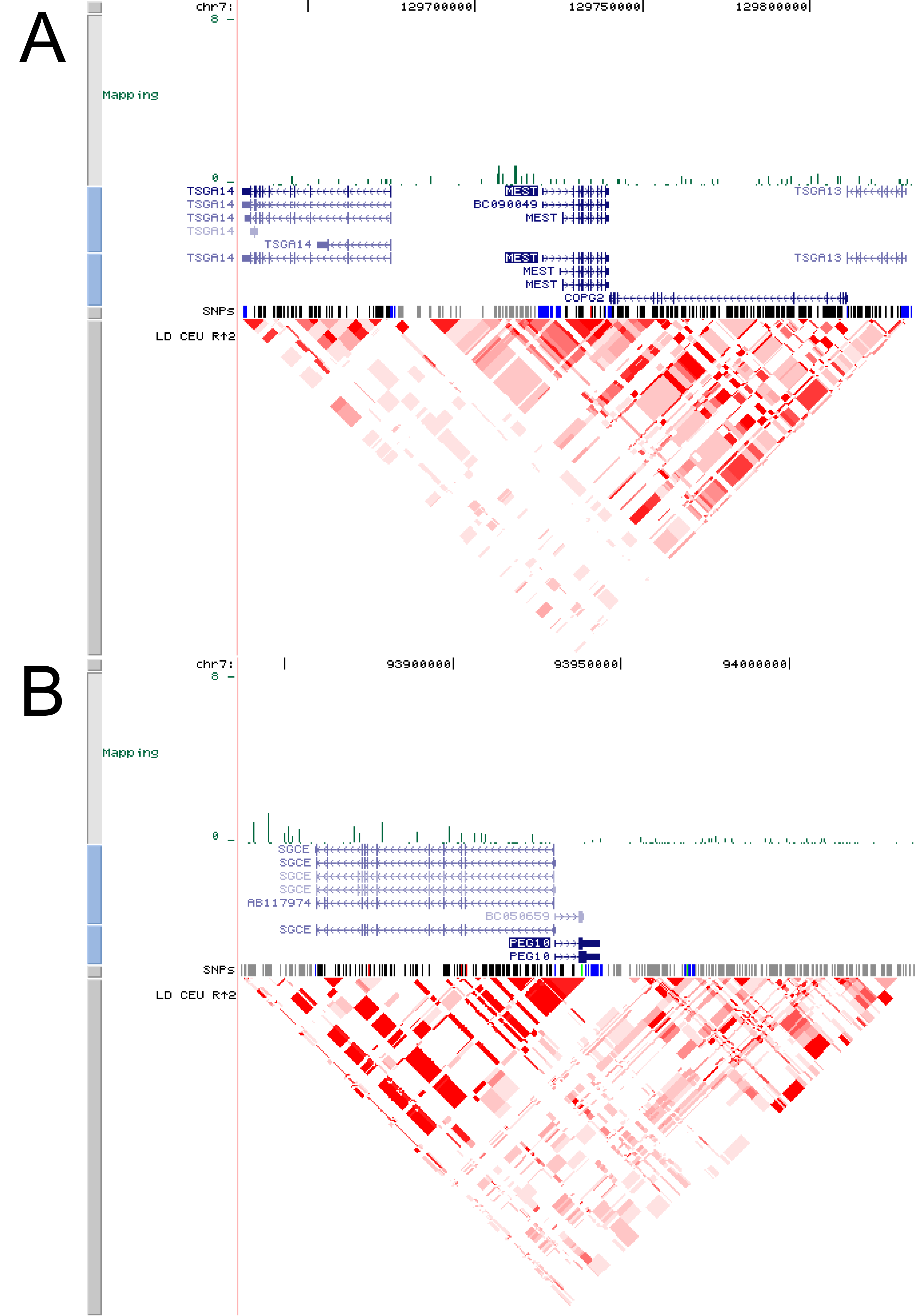

Supplement: Figure S8 — Association mapping of allelic imbalance to regulatory haplotypes for MEST (A) and PEG10 (B). (4.54 MB TIF) [file pgen.1000006.s009.tif]

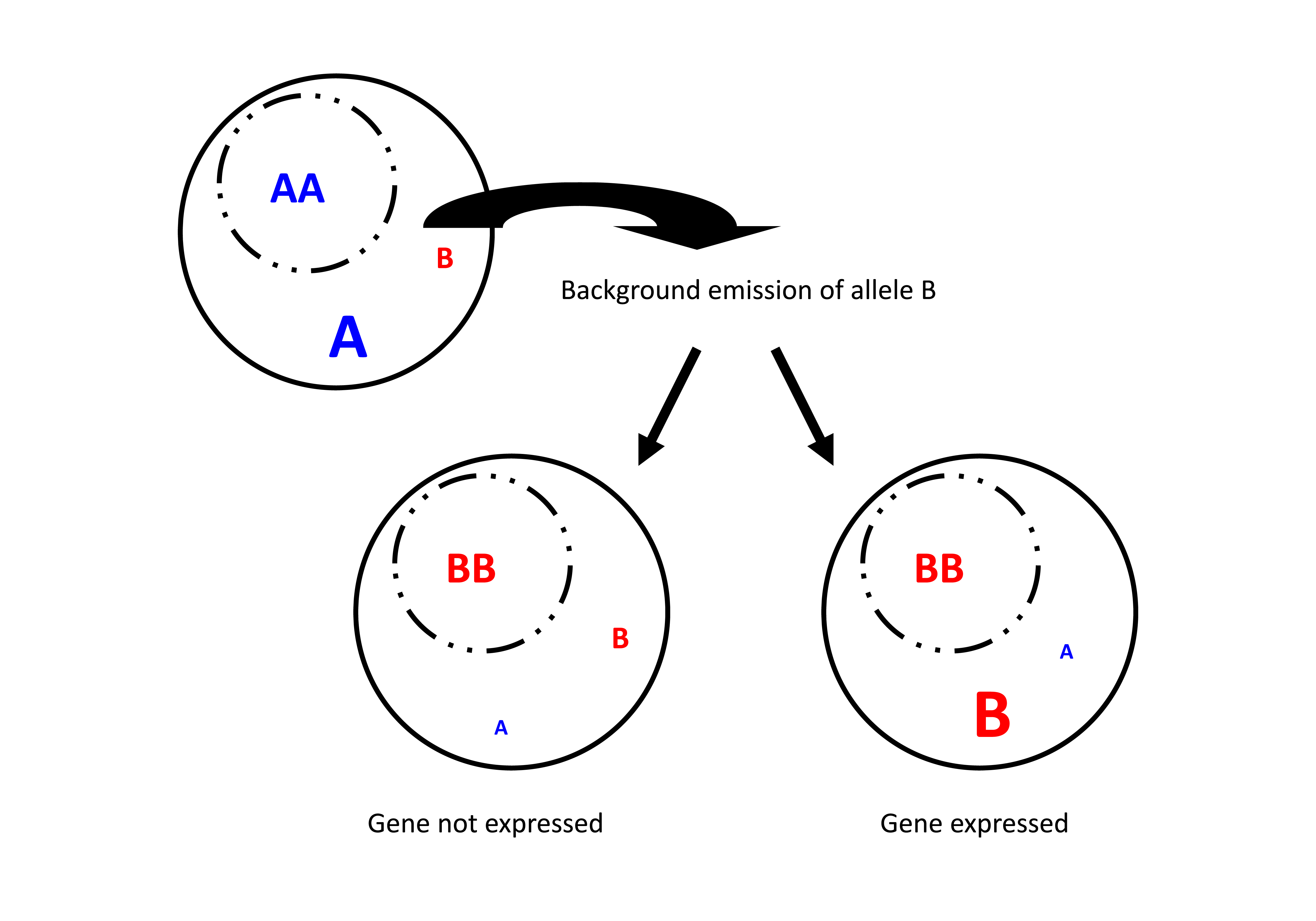

Supplement: Figure S9 — Method used for the detection of transcript expression. See Materials and Methods for details. (1.86 MB TIF) [file pgen.1000006.s010.tif]

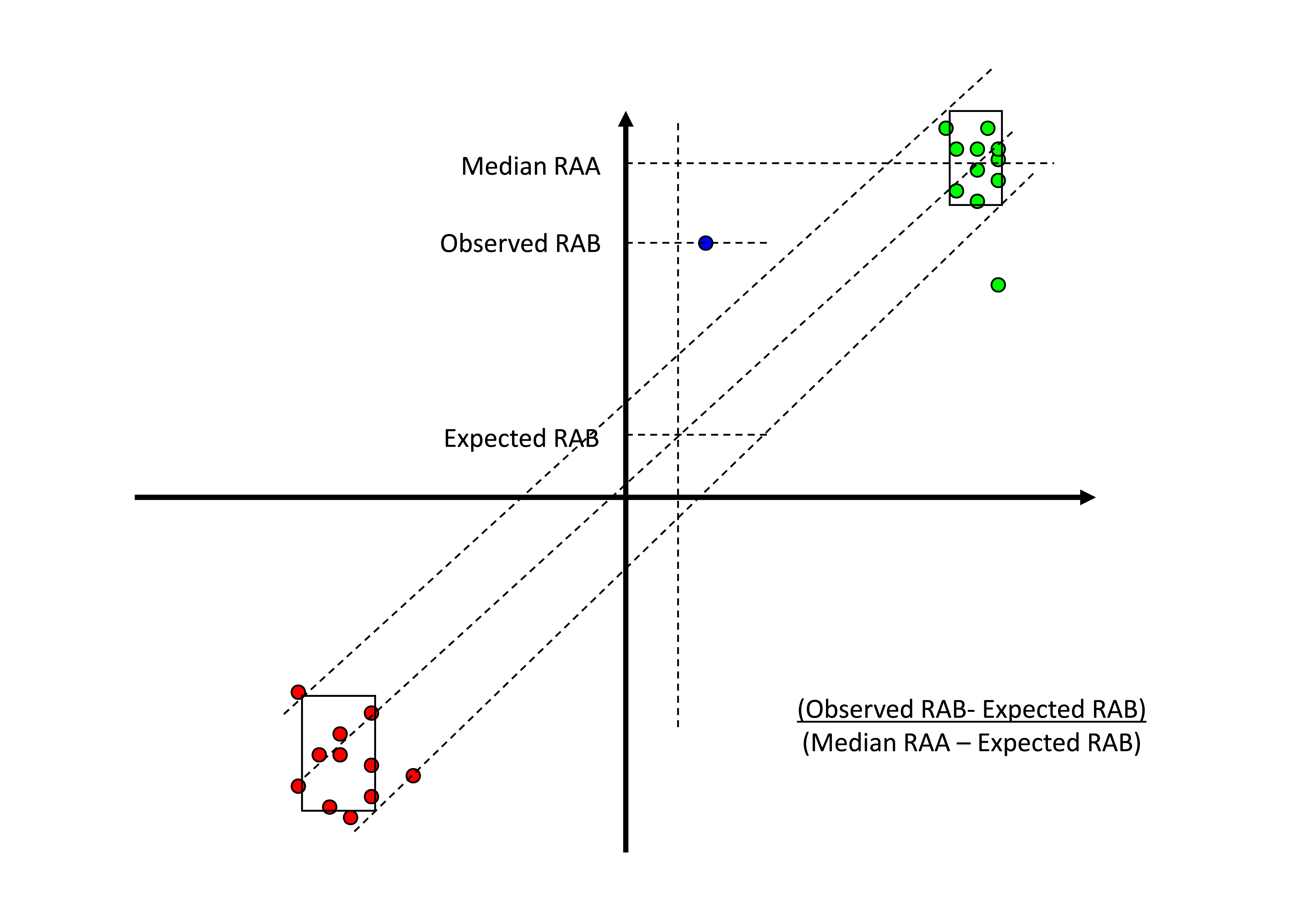

Supplement: Figure S10 — Individual assessment of differential allelic expression on the Illumina ASE assay. See Materials and Methods for details. (1.93 MB TIF) [file pgen.1000006.s011.tif]

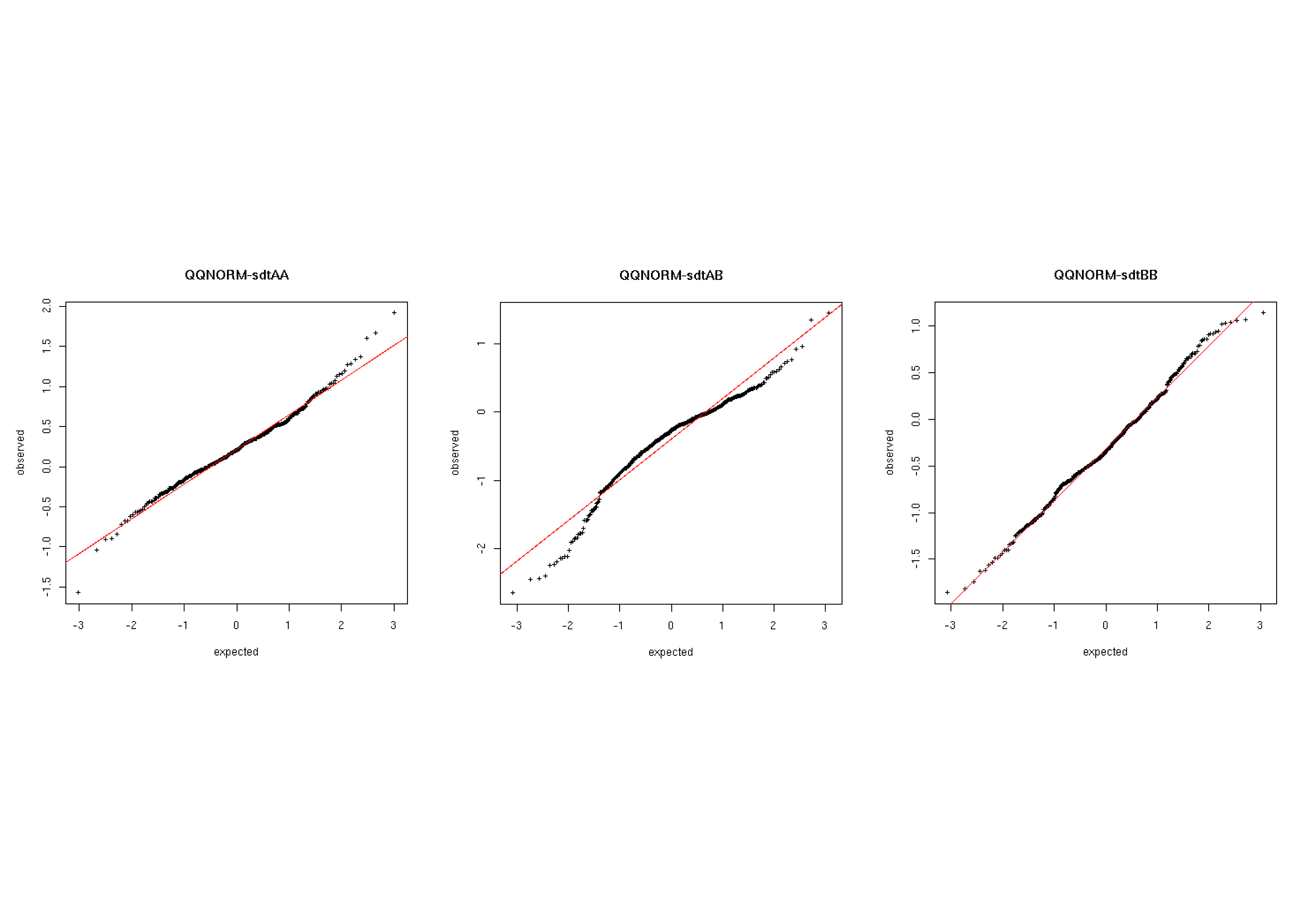

Supplement: Figure S11 — Variance-based assessment of differential allelic expression on the Illumina ASE assay. See Materials and Methods for details. (1.91 MB TIF) [file pgen.1000006.s012.tif]

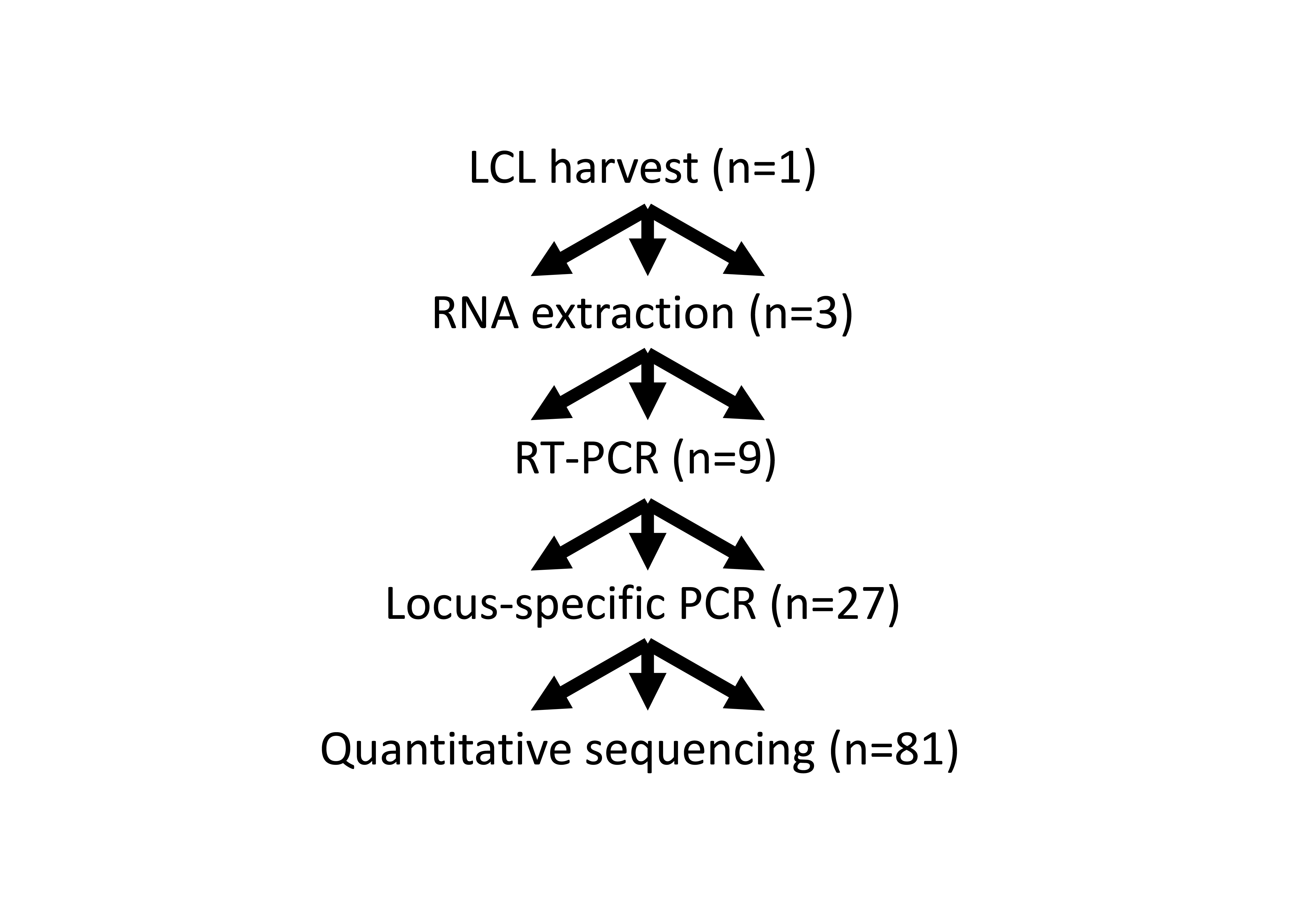

Supplement: Figure S12 — Estimation of experimental variability in quantitative sequencing assay. We performed, for two genes (and five individuals), triplicates of each experimental step: from one cell harvest we extract RNA three times independently. Each extract was then subject to three independent RT-PCRs and each aliquot was amplified three times by locus-specific PCR. Finally, PCR products were sequenced three times and allelic imbalance estimated using PeakPicker v2.0. (1.54 MB TIF) [file pgen.1000006.s013.tif]
