# Supplementary material for: Differential Allelic Expression in the Human Genome: A Robust Approach To Identify Genetic and Epigenetic Cis-Acting Mechanisms Regulating Gene Expression
Source: PLoS Genet. 2008 Feb 29;4(2):e1000006. doi: 10.1371/journal.pgen.1000006 (PMC2265535; doi:10.1371/journal.pgen.1000006)
Supplement: Table S1 — List of the 2,968 SNPs analyzed using the Illumina ASE assay. Origin. Displays if the gene is located in a ENCODE region, on chromosome 21 or 22 and whether the genes was included for its potential involvement in disease etiology. Intron/exon. SNPs in 3′UTR are shown as “exon”. (0.09 MB PDF) [file pgen.1000006.s014.pdf]

Supplemental Table 1. List of the 2,968 SNPs analyzed using the Illumina ASE assay

rs	Gene	Panel	Origin	Intron/Exon	Expressed
rs135106	A4GALT	ENCODE	chr22	exon	yes
rs3788594	A4GALT	ENCODE	chr22	intron	
rs8073904	AATK	CANCER		exon	
rs2230805	ABCA1	CANCER		exon	
rs2246841	ABCA1	CANCER		exon	
rs4149341	ABCA1	CANCER		exon	
rs2230806	ABCA1	CANCER		exon	
rs17064	ABCB1	CANCER		exon	yes
rs3842	ABCB1	CANCER		exon	yes
rs2235036	ABCB1	CANCER		exon	
rs3213619	ABCB1	ENCODE	disease	exon	
rs2229109	ABCB1	ENCODE	disease	exon	
rs17064	ABCB1	ENCODE	disease	exon	yes
rs2302387	ABCB4	CANCER		exon	yes
rs1202283	ABCB4	CANCER		exon	
rs8187799	ABCB4	CANCER		exon	
rs8129984	ABCC13	ENCODE	chr21	exon	
rs17273981	ABCC13	ENCODE	chr21	intron	
rs2273697	ABCC2	CANCER		exon	
rs8187694	ABCC2	CANCER		exon	
rs8187706	ABCC2	CANCER		exon	
rs562	ABCC5	CANCER		exon	yes
rs1132776	ABCC5	CANCER		exon	
rs3749442	ABCC5	CANCER		exon	
rs915842	ABCG1	ENCODE	chr21	exon	
rs183436	ABCG1	ENCODE	chr21	intron	yes
rs2231142	ABCG2	CANCER		exon	
rs2231137	ABCG2	CANCER		exon	
rs1056209	ABL1	CANCER		exon	yes
rs7457	ABL1	CANCER		exon	yes
rs2229070	ABL1	CANCER		exon	
rs2274230	ABL2	CANCER		exon	yes
rs1318056	ABL2	CANCER		exon	
rs8176720	ABO	CANCER		exon	
rs8176745	ABO	CANCER		exon	
rs2276643	ACCN4	ENCODE	encode	exon	
rs1050146	ACTG2	CANCER		exon	
rs10206004	ACVR1	CANCER		exon	
rs1146031	ACVR1	CANCER		exon	
rs1134723	ACVR1	CANCER		exon	
rs11831802	ACVR1B	CANCER		exon	
rs2854464	ACVR1B	CANCER		exon	
rs3058	ACVR1B	CANCER		exon	
rs4556933	ACVR1C	CANCER		exon	
rs1046048	ACVR2B	CANCER		exon	yes
rs7431353	ACVR2B	CANCER		exon	
rs2280091	ADAM33	ENCODE	disease	exon	
rs3746631	ADAM33	ENCODE	disease	exon	
rs2738	ADAMTS1	ENCODE	chr21	exon	

rs	Gene	Panel	Origin	Intron/Exon	Expressed
rs229042	ADAMTS1	ENCODE	chr21	intron	
rs25754	ADAMTS12	CANCER		exon	
rs3813474	ADAMTS12	CANCER		exon	
rs2830585	ADAMTS5	ENCODE	chr21	exon	
rs226794	ADAMTS5	ENCODE	chr21	exon	
rs1444269	ADAMTS5	ENCODE	chr21	exon	
rs17653989	ADAMTS5	ENCODE	chr21	intron	
rs2838820	ADARB1	ENCODE	chr21	exon	yes
rs3788152	ADARB1	ENCODE	chr21	intron	
rs1610037	ADCYAP1	CANCER		exon	
rs2231187	ADCYAP1	CANCER		exon	
rs1006567	ADCYAP1	CANCER		exon	
rs7539542	ADIPOR1	ENCODE	disease	exon	yes
rs1342387	ADIPOR1	ENCODE	disease	intron	yes
rs8192446	ADORA2A	ENCODE	chr22	exon	
rs4530	ADORA2A	ENCODE	chr22	exon	
rs17004919	ADORA2A	ENCODE	chr22	intron	
rs2294918	ADPN	ENCODE	chr22	exon	
rs738409	ADPN	ENCODE	chr22	exon	
rs2076213	ADPN	ENCODE	chr22	exon	
rs4823104	ADPN	ENCODE	chr22	intron	
rs1048101	ADRA1A	ENCODE	disease	exon	
rs17333700	ADRA1A	ENCODE	disease	exon	
rs3739216	ADRA1A	ENCODE	disease	exon	
rs7825488	ADRA1A	ENCODE	disease	intron	
rs1042713	ADRB2	ENCODE	disease	exon	
rs16980612	ADRBK2	ENCODE	chr22	exon	
rs1988253	ADRBK2	ENCODE	chr22	intron	
rs739863	AFF4	ENCODE	encode	exon	yes
rs42395	AFF4	ENCODE	encode	exon	yes
rs2525485	AFF4	ENCODE	encode	intron	yes
rs16849439	AFP	CANCER		exon	
rs13847	AGPAT3	ENCODE	chr21	exon	yes
rs2838438	AGPAT3	ENCODE	chr21	intron	
rs4762	AGT	ENCODE	disease	exon	
rs7079	AGT	ENCODE	disease	exon	
rs11568044	AGT	ENCODE	disease	intron	
rs1800766	AGTR1	CANCER		exon	
rs5182	AGTR1	CANCER		exon	
rs5189	AGTR1	CANCER		exon	
rs4426527	AGXT	CANCER		exon	
rs2066853	AHR	CANCER		exon	yes
rs7292968	AIFL	ENCODE	chr22	exon	
rs4978877	AKAP2	CANCER		exon	yes
rs914358	AKAP2	CANCER		exon	
rs2498799	AKT1	CANCER		exon	yes
rs1321	ALG12	ENCODE	chr22	exon	yes
rs9627786	ALG12	ENCODE	chr22	intron	
rs1881420	ALK	CANCER		exon	
rs2246745	ALK	CANCER		exon	
rs2293564	ALK	CANCER		exon	

rs	Gene	Panel	Origin	Intron/Exon	Expressed
rs2307214	ALOX12	CANCER		exon	
rs312462	ALOX12	CANCER		exon	
rs11571365	ALOX12	CANCER		exon	
rs757537	ANKRD43	ENCODE	encode	exon	
rs9563	AOC3	CANCER		exon	
rs7286796	AP1B1	ENCODE	chr22	exon	yes
rs2071578	AP1B1	ENCODE	chr22	intron	
rs1105307	APBA1	CANCER		exon	
rs12351346	APBA1	CANCER		exon	
rs3751555	APBA2	CANCER		exon	
rs397768	APC	CANCER		exon	yes
rs448475	APC	CANCER		exon	yes
rs866006	APC	CANCER		exon	yes
rs2229992	APC	CANCER		exon	
rs10932806	APEG1	ENCODE	encode	exon	
rs748515	APEG1	ENCODE	encode	intron	
rs2266788	APOA5	ENCODE	encode	exon	
rs7290153	APOBEC3A	ENCODE	chr22	exon	
rs4365560	APOBEC3A	ENCODE	chr22	intron	
rs2281106	APOBEC3C	ENCODE	chr22	intron	yes
rs5750735	APOBEC3F	ENCODE	chr22	exon	yes
rs2076101	APOBEC3F	ENCODE	chr22	exon	yes
rs5750727	APOBEC3F	ENCODE	chr22	intron	
rs5757465	APOBEC3G	ENCODE	chr22	exon	yes
rs12160242	APOBEC3G	ENCODE	chr22	intron	yes
rs8178847	APOH	ENCODE	disease	exon	
rs6933	APOH	ENCODE	disease	exon	
rs4790914	APOH	ENCODE	disease	intron	
rs1807740	APOL1	ENCODE	chr22	exon	yes
rs2239785	APOL1	ENCODE	chr22	exon	yes
rs9610467	APOL1	ENCODE	chr22	intron	yes
rs2017689	APOL2	ENCODE	chr22	exon	
rs9610460	APOL2	ENCODE	chr22	intron	yes
rs132618	APOL3	ENCODE	chr22	exon	yes
rs132621	APOL3	ENCODE	chr22	intron	yes
rs5995250	APOL4	ENCODE	chr22	exon	
rs132736	APOL4	ENCODE	chr22	exon	
rs2007468	APOL4	ENCODE	chr22	exon	
rs12781	APOL4	ENCODE	chr22	exon	
rs5756098	APOL4	ENCODE	chr22	intron	
rs2076673	APOL5	ENCODE	chr22	exon	
rs2076671	APOL5	ENCODE	chr22	exon	
rs4821439	APOL5	ENCODE	chr22	intron	
rs2413361	APOL6	ENCODE	chr22	exon	
rs5999916	APOL6	ENCODE	chr22	intron	
rs4705870	APXL2	ENCODE	encode	exon	
rs9332968	AR	CANCER		exon	
rs9332969	AR	CANCER		exon	
rs9332971	AR	CANCER		exon	
rs1615111	AREG	CANCER		exon	
rs738535	ARFGAP3	ENCODE	chr22	exon	yes

rs	Gene	Panel	Origin	Intron/Exon	Expressed
rs8136141	ARFGAP3	ENCODE	chr22	exon	
rs4393836	ARFGAP3	ENCODE	chr22	intron	yes
rs244468	ARHGAP26	ENCODE	encode	exon	yes
rs10042074	ARHGAP26	ENCODE	encode	intron	
rs2228226	ARHGAP9	CANCER		exon	yes
rs4703	ARHGDIB	CANCER		exon	yes
rs921	ARHGDIB	CANCER		exon	yes
rs1051861	ARID4A	CANCER		exon	yes
rs11552229	ARNT	CANCER		exon	yes
rs2228099	ARNT	CANCER		exon	yes
rs2229175	ARNT	CANCER		exon	
rs139316	ARP10	ENCODE	chr22	exon	yes
rs139297	ARP10	ENCODE	chr22	exon	yes
rs139279	ARP10	ENCODE	chr22	intron	
rs743616	ARSA	ENCODE	chr22	exon	yes
rs6151429	ARSA	ENCODE	chr22	exon	yes
rs2073748	ARVCF	ENCODE	chr22	exon	
rs165815	ARVCF	ENCODE	chr22	exon	
rs165849	ARVCF	ENCODE	chr22	intron	
rs36571	ASCC2	ENCODE	chr22	exon	yes
rs4823054	ASCC2	ENCODE	chr22	exon	
rs140149	ASCC2	ENCODE	chr22	intron	
rs9782	ASCL1	CANCER		exon	yes
rs968528	ASCL2	CANCER		exon	
rs1029396	ASZ1	ENCODE	encode	exon	
rs9886209	ASZ1	ENCODE	encode	intron	
rs1800889	ATM	CANCER		exon	yes
rs609261	ATM	CANCER		exon	yes
rs3218675	ATM	CANCER		exon	
rs3092910	ATM	CANCER		exon	
rs1047700	ATP10A	CANCER		exon	
rs3816800	ATP10A	CANCER		exon	
rs4906743	ATP10A	CANCER		exon	
rs2076743	ATP10A	CANCER		exon	
rs1320525	ATP11A	ENCODE	encode	exon	yes
rs11616247	ATP11A	ENCODE	encode	exon	
rs423117	ATP11A	ENCODE	encode	intron	yes
rs17728665	ATP5O	ENCODE	encode+chr21	exon	yes
rs2834293	ATP5O	ENCODE	encode+chr21	intron	
rs5992084	ATP6V1E1	ENCODE	chr22	exon	yes
rs3532	ATP6V1E1	ENCODE	chr22	exon	yes
rs2227928	ATR	CANCER		exon	yes
rs2229033	ATR	CANCER		exon	
rs9614791	ATXN10	ENCODE	chr22	exon	yes
rs134860	ATXN10	ENCODE	chr22	intron	yes
rs1053338	ATXN7	CANCER		exon	yes
rs3774729	ATXN7	CANCER		exon	yes
rs1053339	ATXN7	CANCER		exon	
rs393521	AXIN1	CANCER		exon	yes
rs1805105	AXIN1	CANCER		exon	
rs758033	AXIN1	ENCODE	encode	exon	yes

rs	Gene	Panel	Origin	Intron/Exon	Expressed
rs214250	AXIN1	ENCODE	encode	exon	
rs393521	AXIN1	ENCODE	encode	exon	yes
rs12925669	AXIN1	ENCODE	encode	intron	
rs3746887	B3GALT5	CANCER		exon	
rs734412	B3GALT5	CANCER		exon	
rs909182	B3GALT5	CANCER		exon	
rs3746887	B3GALT5	ENCODE	chr21	exon	
rs2222996	B3GALT5	ENCODE	chr21	intron	
rs12149	BACE2	ENCODE	chr21	exon	yes
rs1046210	BACE2	ENCODE	chr21	exon	yes
rs734757	BACE2	ENCODE	chr21	intron	
rs388707	BACH1	ENCODE	chr21	exon	yes
rs1153279	BACH1	ENCODE	chr21	intron	
rs1043782	BAG4	CANCER		exon	yes
rs210135	BAK1	CANCER		exon	yes
rs1048108	BARD1	CANCER		exon	yes
rs2070096	BARD1	CANCER		exon	yes
rs2229571	BARD1	CANCER		exon	yes
rs4645900	BAX	CANCER		exon	yes
rs704243	BAX	CANCER		exon	yes
rs128941	BC002942	ENCODE	chr22	exon	yes
rs1152781	BCL11B	ENCODE	encode	exon	
rs807450	BCL11B	ENCODE	encode	intron	
rs1564483	BCL2	CANCER		exon	yes
rs4987852	BCL2	CANCER		exon	yes
rs4987853	BCL2	CANCER		exon	
rs3826007	BCL2A1	CANCER		exon	yes
rs4488761	BCL2L13	ENCODE	chr22	exon	yes
rs16980984	BCL2L13	ENCODE	chr22	intron	
rs1950252	BCL2L2	CANCER		exon	
rs910332	BCL2L2	CANCER		exon	
rs1056932	BCL6	CANCER		exon	
rs2229362	BCL6	CANCER		exon	
rs180817	BCR	CANCER		exon	yes
rs5224	BDKRB2	ENCODE	disease	exon	
rs4905459	BDKRB2	ENCODE	disease	intron	
rs6265	BDNF	CANCER		exon	
rs743642	BGN	CANCER		exon	
rs2072392	BID	ENCODE	chr22	exon	
rs11538	BID	ENCODE	chr22	exon	
rs2305001	BID	ENCODE	chr22	exon	
rs181388	BID	ENCODE	chr22	intron	
rs11090143	BIK	ENCODE	chr22	exon	yes
rs3740932	BIRC3	CANCER		exon	
rs5956583	BIRC4	CANCER		exon	yes
rs5958343	BIRC4	CANCER		exon	yes
rs8371	BIRC4	CANCER		exon	yes
rs9856	BIRC4	CANCER		exon	yes
rs17330644	BIRC4	ENCODE	encode	exon	yes
rs1474593	BIRC4	ENCODE	encode	intron	yes
rs1042489	BIRC5	CANCER		exon	yes

rs	Gene	Panel	Origin	Intron/Exon	Expressed
rs2239680	BIRC5	CANCER		exon	
rs2250788	BLK	CANCER		exon	yes
rs1063147	BLM	CANCER		exon	yes
rs17273563	BLM	CANCER		exon	yes
rs17274095	BLM	CANCER		exon	yes
rs235768	BMP2	CANCER		exon	yes
rs3733549	BMP3	CANCER		exon	
rs17563	BMP4	CANCER		exon	
rs1044104	BMP6	CANCER		exon	yes
rs11528010	BMPR1A	CANCER		exon	yes
rs7078571	BMPR1A	CANCER		exon	yes
rs1048829	BMPR2	CANCER		exon	yes
rs1061157	BMPR2	CANCER		exon	yes
rs3731696	BMPR2	CANCER		exon	yes
rs5998478	BPIL2	ENCODE	encode+chr22	exon	
rs2076045	BPIL2	ENCODE	encode+chr22	intron	
rs16940	BRCA1	CANCER		exon	yes
rs1799966	BRCA1	CANCER		exon	yes
rs799917	BRCA1	CANCER		exon	yes
rs16942	BRCA1	CANCER		exon	
rs144848	BRCA2	CANCER		exon	yes
rs15869	BRCA2	CANCER		exon	yes
rs1801406	BRCA2	CANCER		exon	yes
rs543304	BRCA2	CANCER		exon	yes
rs2056844	BRWD1	ENCODE	encode+chr21	exon	yes
rs1041439	BRWD1	ENCODE	encode+chr21	exon	yes
rs2836933	BRWD1	ENCODE	encode+chr21	exon	
rs13625	BRWD1	ENCODE	encode+chr21	exon	yes
rs2297255	BRWD1	ENCODE	encode+chr21	exon	yes
rs8259	BSG	CANCER		exon	yes
rs1057403	BTK	CANCER		exon	yes
rs700	BTK	CANCER		exon	yes
rs6971	BZRP	ENCODE	chr22	exon	yes
rs138908	BZRP	ENCODE	chr22	intron	
rs2512900	C11orf39	ENCODE	encode	exon	
rs2585	C11orf43	ENCODE	encode	exon	
rs2943	C14orf154	ENCODE	encode	exon	
rs12886549	C14orf154	ENCODE	encode	intron	yes
rs1045001	C16orf33	ENCODE	encode	exon	yes
rs922210	C18orf20	ENCODE	encode	exon	
rs1984359	C18orf20	ENCODE	encode	intron	
rs7290488	C1QTNF6	ENCODE	chr22	exon	yes
rs229519	C1QTNF6	ENCODE	chr22	exon	yes
rs6060450	C20orf173	ENCODE	encode	exon	
rs17092750	C20orf173	ENCODE	encode	exon	
rs4911494	C20orf44	ENCODE	encode	exon	yes
rs2150392	C21orf100	ENCODE	chr21	exon	
rs2150391	C21orf100	ENCODE	chr21	intron	
rs8131523	C21orf123	ENCODE	chr21	exon	
rs2838917	C21orf123	ENCODE	chr21	exon	
rs2838305	C21orf125	ENCODE	chr21	exon	

rs	Gene	Panel	Origin	Intron/Exon	Expressed
rs2838303	C21orf125	ENCODE	chr21	exon	
rs1865758	C21orf125	ENCODE	chr21	intron	
rs220110	C21orf128	ENCODE	chr21	exon	
rs2837029	C21orf13	ENCODE	encode+chr21	exon	yes
rs2837009	C21orf13	ENCODE	encode+chr21	intron	
rs8738	C21orf18	ENCODE	chr21	exon	yes
rs16993814	C21orf18	ENCODE	chr21	exon	
rs2835239	C21orf18	ENCODE	chr21	exon	
rs2835246	C21orf18	ENCODE	chr21	intron	yes
rs10854387	C21orf24	ENCODE	chr21	exon	
rs3746906	C21orf25	ENCODE	chr21	exon	yes
rs9985096	C21orf25	ENCODE	chr21	exon	
rs3168	C21orf25	ENCODE	chr21	exon	yes
rs13050556	C21orf25	ENCODE	chr21	intron	yes
rs2838497	C21orf33	ENCODE	chr21	exon	
rs2838491	C21orf33	ENCODE	chr21	intron	yes
rs11702555	C21orf5	ENCODE	chr21	exon	yes
rs3827183	C21orf5	ENCODE	chr21	exon	yes
rs4817788	C21orf5	ENCODE	chr21	exon	
rs2187300	C21orf5	ENCODE	chr21	intron	
rs9749	C21orf51	ENCODE	chr21	exon	yes
rs10483018	C21orf51	ENCODE	chr21	intron	
rs2833955	C21orf62	ENCODE	encode+chr21	exon	
rs1557264	C21orf62	ENCODE	encode+chr21	intron	
rs7283236	C21orf69	ENCODE	chr21	exon	yes
rs2832236	C21orf7	ENCODE	chr21	exon	yes
rs9978281	C21orf7	ENCODE	chr21	intron	yes
rs2833907	C21orf77	ENCODE	encode+chr21	exon	
rs2833906	C21orf77	ENCODE	encode+chr21	intron	
rs17179966	C21orf84	ENCODE	chr21	exon	
rs162383	C21orf84	ENCODE	chr21	intron	
rs6518223	C21orf86	ENCODE	chr21	exon	
rs10098	C21orf86	ENCODE	chr21	intron	
rs1047978	C21orf91	ENCODE	chr21	exon	yes
rs2258119	C21orf91	ENCODE	chr21	intron	yes
rs1556321	C21orf93	ENCODE	chr21	exon	
rs2838869	C21orf93	ENCODE	chr21	intron	
rs2831368	C21orf94	ENCODE	chr21	exon	
rs2831347	C21orf94	ENCODE	chr21	intron	
rs9704	C22orf13	ENCODE	chr22	exon	yes
rs1008550	C22orf13	ENCODE	chr22	intron	
rs2298375	C22orf15	ENCODE	chr22	exon	
rs9153	C22orf16	ENCODE	chr22	exon	
rs6002555	C22orf18	ENCODE	chr22	exon	yes
rs5758511	C22orf18	ENCODE	chr22	intron	yes
rs737976	C22orf19	ENCODE	chr22	exon	yes
rs1049534	C22orf19	ENCODE	chr22	exon	yes
rs139859	C22orf23	ENCODE	chr22	exon	
rs16998818	C22orf5	ENCODE	chr22	exon	
rs1059804	C22orf5	ENCODE	chr22	exon	
rs5750562	C22orf5	ENCODE	chr22	intron	

rs	Gene	Panel	Origin	Intron/Exon	Expressed
rs6007594	C22orf8	ENCODE	chr22	exon	yes
rs226505	C22orf8	ENCODE	chr22	intron	
rs1043731	C22orf9	ENCODE	chr22	exon	yes
rs2072720	C22orf9	ENCODE	chr22	intron	
rs406658	C4B	CANCER		exon	
rs2545166	C5orf18	CANCER		exon	yes
rs3317	C5orf18	CANCER		exon	yes
rs3733963	C5orf18	CANCER		exon	
rs4640856	C6orf148	ENCODE	encode	exon	
rs7771553	C6orf148	ENCODE	encode	exon	
rs9352000	C6orf150	ENCODE	encode	exon	yes
rs610913	C6orf150	ENCODE	encode	exon	yes
rs311678	C6orf150	ENCODE	encode	exon	
rs311677	C6orf150	ENCODE	encode	intron	yes
rs2072409	C7orf16	ENCODE	disease	exon	
rs3735422	C7orf16	ENCODE	disease	exon	
rs34161	C7orf16	ENCODE	disease	intron	
rs913770	C9orf106	ENCODE	encode	exon	
rs9624395	CABIN1	ENCODE	chr22	exon	
rs5760180	CABIN1	ENCODE	chr22	intron	yes
rs4820812	CABP7	ENCODE	chr22	exon	
rs737978	CABP7	ENCODE	chr22	intron	
rs3747179	CACNA1I	ENCODE	chr22	exon	
rs136852	CACNA1I	ENCODE	chr22	exon	
rs5757731	CACNA1I	ENCODE	chr22	intron	
rs2291068	CACNG6	ENCODE	encode	exon	
rs158194	CACNG6	ENCODE	encode	intron	
rs2271627	CAPG	CANCER		exon	yes
rs2975767	CAPN10	CANCER		exon	yes
rs3749166	CAPN10	CANCER		exon	
rs3173936	CAPZA2	ENCODE	encode	exon	yes
rs3807999	CAPZA2	ENCODE	encode	intron	yes
rs9610775	CARD10	ENCODE	chr22	exon	
rs3817803	CARD10	ENCODE	chr22	exon	
rs5756697	CARD10	ENCODE	chr22	intron	
rs3135500	CARD15	CANCER		exon	yes
rs2066842	CARD15	CANCER		exon	
rs5743266	CARD15	CANCER		exon	
rs13006529	CASP10	CANCER		exon	yes
rs3900115	CASP10	CANCER		exon	yes
rs1049216	CASP3	CANCER		exon	yes
rs1042891	CASP6	CANCER		exon	yes
rs1045485	CASP8	CANCER		exon	
rs3769823	CASP8	CANCER		exon	
rs1045487	CASP8	CANCER		exon	
rs2308941	CASP9	CANCER		exon	
rs2234940	CAT	CANCER		exon	
rs2266625	CAT	CANCER		exon	
rs769217	CAT	CANCER		exon	
rs8042868	CATSPER2	ENCODE	encode	exon	yes
rs12443102	CATSPER2	ENCODE	encode	exon	yes

rs	Gene	Panel	Origin	Intron/Exon	Expressed
rs17727871	CATSPER2	ENCODE	encode	intron	
rs6867	CAV1	CANCER		exon	yes
rs8713	CAV1	CANCER		exon	yes
rs9920	CAV1	CANCER		exon	
rs1049334	CAV1	CANCER		exon	
rs8940	CAV2	CANCER		exon	
rs1047417	CBL	CANCER		exon	yes
rs11217234	CBL	CANCER		exon	
rs2511844	CBL	CANCER		exon	
rs2305035	CBLB	CANCER		exon	yes
rs3772534	CBLB	CANCER		exon	
rs7649466	CBLB	ENCODE	disease	exon	yes
rs2305035	CBLB	ENCODE	disease	exon	yes
rs3772534	CBLB	ENCODE	disease	exon	
rs1042852	CBLB	ENCODE	disease	exon	yes
rs20572	CBR1	ENCODE	chr21	exon	yes
rs1005696	CBR1	ENCODE	chr21	intron	yes
rs706209	CBS	ENCODE	chr21	exon	yes
rs3788050	CBS	ENCODE	chr21	intron	
rs2235140	CBX6	ENCODE	chr22	exon	yes
rs9611041	CBX6	ENCODE	chr22	intron	
rs6448456	CCKAR	CANCER		exon	
rs1042048	CCKBR	CANCER		exon	
rs1805002	CCKBR	CANCER		exon	
rs4586	CCL2	ENCODE	disease	exon	
rs1065341	CCL5	ENCODE	disease	exon	
rs3817655	CCL5	ENCODE	disease	intron	yes
rs769242	CCNA2	CANCER		exon	yes
rs3212898	CCND1	CANCER		exon	
rs3212905	CCND1	CANCER		exon	
rs3862792	CCND1	CANCER		exon	
rs1049612	CCND2	CANCER		exon	yes
rs3217926	CCND2	CANCER		exon	yes
rs3217933	CCND2	CANCER		exon	yes
rs3217921	CCND2	CANCER		exon	
rs9529	CCND3	CANCER		exon	yes
rs3218102	CCND3	CANCER		exon	
rs1406	CCNE1	CANCER		exon	yes
rs4150052	CCNG2	CANCER		exon	yes
rs2266692	CCNH	CANCER		exon	
rs746492	CCR5	CANCER		exon	
rs1800023	CCR5	CANCER		exon	
rs2269714	CD1A	CANCER		exon	
rs366316	CD1A	CANCER		exon	
rs2269715	CD1A	CANCER		exon	
rs699738	CD2	CANCER		exon	
rs1544766	CD209	ENCODE	disease	exon	
rs10410342	CD209	ENCODE	disease	intron	
rs2556	CD34	CANCER		exon	
rs7572	CD34	CANCER		exon	
rs3820521	CD34	CANCER		exon	

rs	Gene	Panel	Origin	Intron/Exon	Expressed
rs8193	CD44	CANCER		exon	yes
rs9666607	CD44	CANCER		exon	yes
rs13347	CD44	CANCER		exon	
rs7357	CD59	CANCER		exon	yes
rs842	CD59	CANCER		exon	yes
rs1049659	CD81	CANCER		exon	
rs1063316	CD81	CANCER		exon	
rs4755266	CD82	CANCER		exon	
rs1129055	CD86	CANCER		exon	yes
rs2681417	CD86	CANCER		exon	yes
rs1871446	CDC2	CANCER		exon	yes
rs3731560	CDC25A	CANCER		exon	
rs8156	CDC25B	CANCER		exon	yes
rs1042124	CDC25C	CANCER		exon	
rs2302341	CDC2L5	CANCER		exon	yes
rs3735135	CDC2L5	CANCER		exon	yes
rs653343	CDC42BPG	ENCODE	encode	exon	
rs7933683	CDC42BPG	ENCODE	encode	exon	
rs7936466	CDC42BPG	ENCODE	encode	exon	
rs647145	CDC42BPG	ENCODE	encode	intron	
rs2281098	CDC42EP1	ENCODE	chr22	exon	
rs2235335	CDC42EP1	ENCODE	chr22	intron	
rs1801552	CDH1	CANCER		exon	
rs1801552	CDH1	ENCODE	disease	exon	yes
rs3912073	CDH1	ENCODE	disease	intron	
rs28216	CDH11	CANCER		exon	
rs17707862	CDH13	CANCER		exon	
rs6565105	CDH13	CANCER		exon	
rs1048613	CDH13	CANCER		exon	
rs1051623	CDH17	CANCER		exon	
rs2243518	CDH17	CANCER		exon	
rs9417	CDH17	CANCER		exon	
rs1041985	CDH2	ENCODE	encode	exon	yes
rs1886699	CDH3	CANCER		exon	
rs2274239	CDH3	CANCER		exon	
rs2296409	CDH3	CANCER		exon	
rs1946482	CDK10	CANCER		exon	yes
rs2069398	CDK2	CANCER		exon	yes
rs42039	CDK6	CANCER		exon	yes
rs8083	CDK9	CANCER		exon	yes
rs1801270	CDKN1A	CANCER		exon	
rs3176359	CDKN1A	CANCER		exon	
rs7330	CDKN1B	CANCER		exon	yes
rs34330	CDKN1B	CANCER		exon	
rs11515	CDKN2A	CANCER		exon	yes
rs3088440	CDKN2A	CANCER		exon	yes
rs3731253	CDKN2A	CANCER		exon	
rs3217984	CDKN2B	CANCER		exon	
rs3217986	CDKN2B	CANCER		exon	
rs3217992	CDKN2B	CANCER		exon	
rs1063192	CDKN2B	CANCER		exon	

rs	Gene	Panel	Origin	Intron/Exon	Expressed
rs12855	CDKN2C	CANCER		exon	yes
rs8102519	CEACAM1	CANCER		exon	
rs12691	CEBPA	CANCER		exon	
rs707656	CEBPA	CANCER		exon	
rs7289141	CECR1	ENCODE	chr22	exon	
rs2231495	CECR1	ENCODE	chr22	exon	yes
rs17807317	CECR1	ENCODE	chr22	exon	
rs1034859	CECR5	ENCODE	chr22	exon	yes
rs740422	CECR5	ENCODE	chr22	intron	
rs971768	CECR6	ENCODE	chr22	exon	yes
rs8141744	CELSR1	ENCODE	chr22	exon	
rs4044210	CELSR1	ENCODE	chr22	exon	
rs6007897	CELSR1	ENCODE	chr22	exon	yes
rs7285515	CELSR1	ENCODE	chr22	exon	
rs6007891	CELSR1	ENCODE	chr22	exon	
rs3748433	CEP250	ENCODE	encode	exon	yes
rs2274238	CEP250	ENCODE	encode	exon	yes
rs3818441	CEP250	ENCODE	encode	intron	yes
rs13057352	CERK	ENCODE	chr22	exon	
rs2748348	CERK	ENCODE	chr22	exon	yes
rs135668	CERK	ENCODE	chr22	intron	
rs2236639	CESK1	ENCODE	chr22	exon	
rs1061147	CFH	ENCODE	disease	exon	
rs800292	CFH	ENCODE	disease	exon	
rs1065489	CFH	ENCODE	disease	exon	
rs512900	CFH	ENCODE	disease	intron	
rs1042077	CFTR	CANCER		exon	
rs1042180	CFTR	CANCER		exon	
rs1800136	CFTR	CANCER		exon	
rs1800136	CFTR	ENCODE	disease+encode	exon	
rs766874	CFTR	ENCODE	disease+encode	exon	
rs2283054	CFTR	ENCODE	disease+encode	intron	
rs1812240	CGI-96	ENCODE	chr22	exon	
rs1418823	CGN	ENCODE	encode	exon	
rs12038198	CGN	ENCODE	encode	exon	
rs7552458	CGN	ENCODE	encode	intron	
rs8596	CHAF1B	ENCODE	chr21	exon	yes
rs218628	CHAF1B	ENCODE	chr21	intron	
rs8178991	CHAT	ENCODE	disease	exon	
rs8178990	CHAT	ENCODE	disease	exon	
rs3810950	CHAT	ENCODE	disease	exon	
rs3750752	CHAT	ENCODE	disease	intron	
rs2066726	CHC1	CANCER		exon	
rs2227975	CHC1	CANCER		exon	
rs2227977	CHC1	CANCER		exon	
rs2272457	CHD2	CANCER		exon	yes
rs4777755	CHD2	CANCER		exon	yes
rs964034	CHD2	CANCER		exon	
rs506504	CHEK1	CANCER		exon	yes
rs1805129	CHEK2	CANCER		exon	
rs9625537	CHEK2	CANCER		exon	

rs	Gene	Panel	Origin	Intron/Exon	Expressed
rs3741490	CHFR	CANCER		exon	yes
rs729940	CHGA	CANCER		exon	
rs941581	CHGA	CANCER		exon	
rs1056825	CHI3L2	CANCER		exon	yes
rs1056831	CHI3L2	CANCER		exon	yes
rs8535	CHI3L2	CANCER		exon	yes
rs8142477	CHKB	ENCODE	chr22	exon	
rs877292	CHKB	ENCODE	chr22	exon	yes
rs2073604	CHKB	ENCODE	chr22	intron	
rs2051358	CHODL	ENCODE	chr21	exon	
rs1043833	CHPF	ENCODE	encode	exon	yes
rs2072659	CHRNB2	ENCODE	disease	exon	
rs1139564	CIITA	ENCODE	disease	exon	yes
rs4774	CIITA	ENCODE	disease	exon	yes
rs12928665	CIITA	ENCODE	disease	intron	
rs2439	CKMT1B	ENCODE	encode	exon	
rs10518820	CKMT1B	ENCODE	encode	intron	
rs1325774	CLDN10	ENCODE	disease	exon	
rs3751334	CLDN10	ENCODE	disease	exon	
rs7333503	CLDN10	ENCODE	disease	intron	
rs17862175	CLDN12	ENCODE	encode	exon	yes
rs917660	CLDN12	ENCODE	encode	intron	yes
rs219780	CLDN14	ENCODE	chr21	exon	
rs128494	CLDN14	ENCODE	chr21	intron	
rs1127155	CLDN4	CANCER		exon	
rs11316	CLDN4	CANCER		exon	
rs8629	CLDN4	CANCER		exon	
rs10314	CLDN5	ENCODE	chr22	exon	
rs13433507	CLDN8	ENCODE	chr21	exon	
rs2834601	CLIC6	ENCODE	chr21	exon	yes
rs6517254	CLIC6	ENCODE	chr21	exon	yes
rs7281533	CLIC6	ENCODE	chr21	intron	
rs7224	CLK1	CANCER		exon	yes
rs3747059	CLTCL1	ENCODE	chr22	exon	
rs5746697	CLTCL1	ENCODE	chr22	exon	
rs807459	CLTCL1	ENCODE	chr22	exon	
rs712952	CLTCL1	ENCODE	chr22	exon	
rs1060376	CLTCL1	ENCODE	chr22	exon	
rs1061325	CLTCL1	ENCODE	chr22	exon	
rs2073738	CLTCL1	ENCODE	chr22	exon	
rs738904	CLTCL1	ENCODE	chr22	exon	
rs762529	CLTCL1	ENCODE	chr22	intron	
rs7867	COL18A1	CANCER		exon	
rs9975285	COL18A1	CANCER		exon	
rs17004785	COL18A1	CANCER		exon	
rs7499	COL18A1	ENCODE	chr21	exon	
rs1050351	COL18A1	ENCODE	chr21	exon	
rs2236467	COL18A1	ENCODE	chr21	exon	
rs879330	COL18A1	ENCODE	chr21	intron	
rs1061237	COL1A1	CANCER		exon	yes
rs412777	COL1A2	CANCER		exon	

rs	Gene	Panel	Origin	Intron/Exon	Expressed
rs1062394	COL1A2	CANCER		exon	
rs2070735	COL4A3	CANCER		exon	
rs1980982	COL6A1	CANCER		exon	
rs9254	COL6A1	ENCODE	chr21	exon	
rs1053312	COL6A1	ENCODE	chr21	exon	
rs3746992	COL6A1	ENCODE	chr21	intron	
rs1042917	COL6A2	ENCODE	chr21	exon	
rs9978055	COL6A2	ENCODE	chr21	intron	
rs740602	COMT	CANCER		exon	
rs2171492	CPA4	CANCER		exon	
rs2306848	CPA4	CANCER		exon	
rs17334859	CPNE1	CANCER		exon	yes
rs2229419	CRAT	ENCODE	encode	exon	
rs3751845	CREBBP	CANCER		exon	
rs3025694	CREBP	CANCER		exon	
rs8139422	CRELD2	ENCODE	chr22	exon	
rs7410773	CRELD2	ENCODE	chr22	intron	
rs2229075	CRK	CANCER		exon	
rs1043242	CRKL	CANCER		exon	yes
rs1548410	CRKL	CANCER		exon	yes
rs2285547	CRKL	CANCER		exon	yes
rs737894	CRKL	ENCODE	chr22	exon	yes
rs2266951	CRKL	ENCODE	chr22	intron	yes
rs872331	CRYAA	ENCODE	chr21	exon	
rs870137	CRYAA	ENCODE	chr21	intron	
rs13050238	CRYZL1	ENCODE	encode+chr21	exon	
rs1006407	CSDC2	ENCODE	chr22	exon	
rs1058885	CSF1	CANCER		exon	
rs3093037	CSF1	CANCER		exon	
rs13117	CSF1R	CANCER		exon	
rs216123	CSF1R	CANCER		exon	
rs25882	CSF2	CANCER		exon	
rs1042658	CSF3	CANCER		exon	
rs2827	CSF3	CANCER		exon	
rs3917981	CSF3R	CANCER		exon	
rs3917991	CSF3R	CANCER		exon	
rs2229730	CSK	CANCER		exon	yes
rs2071501	CSK	CANCER		exon	
rs1801014	CSK	CANCER		exon	
rs805256	CSNK2B	CANCER		exon	yes
rs160279	CSPG2	CANCER		exon	yes
rs188703	CSPG2	CANCER		exon	
rs309559	CSPG2	CANCER		exon	
rs4326559	CTAG2	CANCER		exon	
rs1045480	CTBP1	CANCER		exon	yes
rs6499137	CTCF	CANCER		exon	yes
rs231775	CTLA4	CANCER		exon	
rs1059110	CTNNA1	CANCER		exon	yes
rs2953	CTNNB1	CANCER		exon	yes
rs4135386	CTNNB1	CANCER		exon	
rs8839	CTSD	CANCER		exon	yes

rs	Gene	Panel	Origin	Intron/Exon	Expressed
rs698452	CTSD	CANCER		exon	
rs17571	CTSD	ENCODE	encode	exon	yes
rs2292963	CTSD	ENCODE	encode	intron	
rs13345	CTSH	CANCER		exon	yes
rs3129	CTSH	CANCER		exon	yes
rs4730796	CTTNBP2	ENCODE	encode	exon	
rs4730797	CTTNBP2	ENCODE	encode	intron	
rs1131510	CUL2	CANCER		exon	yes
rs1804430	CXCL12	ENCODE	disease	exon	yes
rs2839695	CXCL12	ENCODE	disease	exon	
rs266088	CXCL12	ENCODE	disease	intron	yes
rs10336	CXCL9	CANCER		exon	yes
rs3733236	CXCL9	CANCER		exon	
rs6571303	CXorf12	ENCODE	encode	exon	yes
rs2266887	CXorf12	ENCODE	encode	intron	
rs137124	CYB5R3	ENCODE	chr22	exon	
rs743887	CYB5R3	ENCODE	chr22	intron	
rs2066852	CYLD	CANCER		exon	yes
rs6163	CYP17A1	ENCODE	disease	exon	
rs10883783	CYP17A1	ENCODE	disease	intron	
rs1048943	CYP1A1	CANCER		exon	
rs2470890	CYP1A2	ENCODE	disease	exon	
rs762551	CYP1A2	ENCODE	disease	intron	
rs10916	CYP1B1	CANCER		exon	yes
rs162549	CYP1B1	CANCER		exon	yes
rs2855658	CYP1B1	CANCER		exon	yes
rs8176345	CYP27B1	ENCODE	disease	exon	
rs769258	CYP2D6	ENCODE	chr22	exon	
rs1058167	CYP2D6	ENCODE	chr22	exon	
rs2515641	CYP2E1	CANCER		exon	
rs966410	CYYR1	ENCODE	chr21	exon	
rs2830239	CYYR1	ENCODE	chr21	exon	
rs2070540	D21S2056E	ENCODE	chr21	exon	yes
rs2276246	D21S2056E	ENCODE	chr21	intron	yes
rs3733801	DAB2	CANCER		exon	
rs1051101	DAD1	CANCER		exon	yes
rs1803479	DAD1	CANCER		exon	yes
rs4981429	DAD1	CANCER		exon	yes
rs1058207	DAP3	CANCER		exon	yes
rs4933	DAP3	CANCER		exon	yes
rs3118863	DAPK1	CANCER		exon	yes
rs1056719	DAPK1	CANCER		exon	
rs3118864	DAPK1	CANCER		exon	
rs1043379	DBC1	CANCER		exon	
rs2274157	DBC1	CANCER		exon	
rs28453	DBC1	CANCER		exon	
rs2229082	DCC	CANCER		exon	
rs9951523	DCC	CANCER		exon	
rs2229080	DCC	ENCODE	disease	exon	
rs9951523	DCC	ENCODE	disease	exon	
rs7242705	DCC	ENCODE	disease	intron	

rs	Gene	Panel	Origin	Intron/Exon	Expressed
rs7441	DCN	CANCER		exon	
rs4647707	DDB2	CANCER		exon	
rs697221	DDIT3	CANCER		exon	yes
rs1049633	DDR1	CANCER		exon	yes
rs8408	DDR1	CANCER		exon	yes
rs1049623	DDR1	CANCER		exon	
rs2298258	DDR2	CANCER		exon	
rs5750609	DDX17	CANCER		exon	yes
rs763121	DDX17	CANCER		exon	yes
rs86796	DDX17	CANCER		exon	yes
rs763121	DDX17	ENCODE	chr22	exon	yes
rs1052639	DDX18	ENCODE	encode	exon	yes
rs311683	DDX43	ENCODE	encode	exon	yes
rs311686	DDX43	ENCODE	encode	exon	yes
rs558198	DDX43	ENCODE	encode	exon	
rs17756426	DDX43	ENCODE	encode	intron	yes
rs487728	DDX6	CANCER		exon	yes
rs488219	DDX6	CANCER		exon	yes
rs1013062	DEK	CANCER		exon	yes
rs5998170	DEPDC5	ENCODE	encode+chr22	exon	yes
rs16989528	DEPDC5	ENCODE	encode+chr22	exon	
rs5998114	DEPDC5	ENCODE	encode+chr22	intron	yes
rs5760062	DERL3	ENCODE	chr22	exon	
rs1058261	DES	CANCER		exon	
rs1058284	DES	ENCODE	encode	exon	
rs12233351	DGCR13	ENCODE	chr22	exon	
rs17743887	DGCR14	ENCODE	chr22	exon	
rs929282	DGCR14	ENCODE	chr22	exon	
rs929283	DGCR14	ENCODE	chr22	intron	
rs6623	DGCR2	ENCODE	chr22	exon	yes
rs2283641	DGCR2	ENCODE	chr22	intron	
rs446213	DGCR6	ENCODE	chr22	exon	
rs408469	DGCR6	ENCODE	chr22	exon	
rs1640299	DGCR8	ENCODE	chr22	exon	yes
rs2269723	DGCR8	ENCODE	chr22	intron	
rs7374	DHCR24	CANCER		exon	yes
rs8990	DHCR24	CANCER		exon	yes
rs945006	DIO3	CANCER		exon	
rs17761627	DIP	ENCODE	chr22	exon	
rs12162984	DIP	ENCODE	chr22	intron	
rs8127941	DIP2A	ENCODE	chr21	exon	
rs2248636	DIP2A	ENCODE	chr21	exon	yes
rs2255397	DIP2A	ENCODE	chr21	exon	yes
rs2070435	DIP2A	ENCODE	chr21	exon	yes
rs7283507	DIP2A	ENCODE	chr21	exon	yes
rs2078203	DIP2A	ENCODE	chr21	intron	yes
rs11122324	DISC1	ENCODE	disease	exon	
rs3738401	DISC1	ENCODE	disease	exon	
rs3081	DISC1	ENCODE	disease	exon	
rs3737597	DISC1	ENCODE	disease	exon	
rs12133766	DISC1	ENCODE	disease	exon	yes

rs	Gene	Panel	Origin	Intron/Exon	Expressed
rs823167	DISC1	ENCODE	disease	intron	
rs9090	DKFZP564O0823	CANCER		exon	yes
rs3796683	DKFZP564O0823	CANCER		exon	
rs17863999	DKFZP686A10121	ENCODE	encode	exon	yes
rs42663	DKFZP686A10121	ENCODE	encode	exon	yes
rs17621789	DKFZP686A10121	ENCODE	encode	exon	
rs17863986	DKFZP686A10121	ENCODE	encode	intron	yes
rs16982614	DKFZp761P1121	ENCODE	chr22	exon	
rs2518827	DKFZp761P1121	ENCODE	chr22	intron	
rs2302154	DKFZp762E1312	ENCODE	encode	exon	
rs12582	DKFZp762E1312	ENCODE	encode	exon	yes
rs17868364	DKFZp762E1312	ENCODE	encode	intron	
rs702681	DKFZP781I1119	ENCODE	encode	exon	yes
rs702680	DKFZP781I1119	ENCODE	encode	intron	yes
rs1044011	DLC1	CANCER		exon	
rs532841	DLC1	CANCER		exon	
rs3739300	DLC1	CANCER		exon	
rs2066575	DLEU1	CANCER		exon	yes
rs7501	DLK1	CANCER		exon	
rs1058009	DLK1	CANCER		exon	
rs1033583	DLL1	CANCER		exon	
rs7383	DMBT1	CANCER		exon	
rs8441	DMBT1	CANCER		exon	
rs1047775	DNAJD1	CANCER		exon	yes
rs17553284	DNAJD1	CANCER		exon	
rs760482	DNAL4	ENCODE	chr22	exon	
rs14040	DNAL4	ENCODE	chr22	exon	yes
rs5757282	DNAL4	ENCODE	chr22	intron	
rs12877	DNASE1L1	CANCER		exon	yes
rs2070807	DNASE1L1	ENCODE	encode	exon	yes
rs17423	DNASE1L1	ENCODE	encode	exon	
rs2283762	DNASE1L1	ENCODE	encode	intron	
rs8111085	DNMT1	CANCER		exon	yes
rs721186	DNMT1	CANCER		exon	
rs10904889	DNMT2	CANCER		exon	yes
rs11254401	DNMT2	CANCER		exon	yes
rs7096233	DNMT2	CANCER		exon	
rs2424932	DNMT3B	CANCER		exon	
rs6058896	DNMT3B	CANCER		exon	
rs7354779	DNMT3L	ENCODE	chr21	exon	
rs3788111	DNMT3L	ENCODE	chr21	intron	
rs11564095	DOLPP1	ENCODE	encode	exon	
rs10760586	DOLPP1	ENCODE	encode	intron	
rs3742836	DPF3	ENCODE	disease	exon	
rs2526935	DPF3	ENCODE	disease	exon	
rs2803956	DPF3	ENCODE	disease	intron	
rs4986918	DRD2	ENCODE	disease	exon	
rs1800499	DRD2	ENCODE	disease	exon	
rs6274	DRD2	ENCODE	disease	exon	
rs2734841	DRD2	ENCODE	disease	intron	
rs1893963	DSC2	CANCER		exon	yes

rs	Gene	Panel	Origin	Intron/Exon	Expressed
rs2297270	DSCAM	ENCODE	chr21	exon	
rs2297263	DSCAM	ENCODE	chr21	exon	
rs2837374	DSCAM	ENCODE	chr21	intron	
rs14194	DSCR2	ENCODE	encode+chr21	exon	yes
rs2245455	DSCR2	ENCODE	encode+chr21	intron	yes
rs3165	DSCR3	CANCER		exon	yes
rs3165	DSCR3	ENCODE	chr21	exon	yes
rs2073355	DSCR3	ENCODE	chr21	intron	
rs2070350	DSCR4	ENCODE	chr21	exon	
rs983553	DSCR4	ENCODE	chr21	intron	
rs2835560	DSCR6	ENCODE	chr21	exon	
rs2277784	DSCR6	ENCODE	chr21	intron	yes
rs3752095	DSG1	CANCER		exon	
rs2076299	DSP	CANCER		exon	
rs4712138	DST	CANCER		exon	yes
rs4715631	DST	CANCER		exon	yes
rs2144407	DST	CANCER		exon	
rs8630	DUSP18	ENCODE	chr22	exon	yes
rs4820893	DUSP18	ENCODE	chr22	intron	
rs3182143	DUSP4	CANCER		exon	
rs11919795	DVL3	CANCER		exon	yes
rs3749231	DVL3	CANCER		exon	yes
rs1803439	DYRK1A	ENCODE	chr21	exon	yes
rs2835726	DYRK1A	ENCODE	chr21	exon	yes
rs12483205	DYRK1A	ENCODE	chr21	intron	yes
rs3213176	E2F1	CANCER		exon	
rs3218171	E2F2	CANCER		exon	
rs3218211	E2F2	CANCER		exon	
rs4134982	E2F3	CANCER		exon	
rs5369	EDN1	CANCER		exon	yes
rs5370	EDN1	CANCER		exon	yes
rs5351	EDNRB	CANCER		exon	yes
rs4885491	EDNRB	CANCER		exon	
rs1801710	EDNRB	CANCER		exon	
rs1390082	EEF1A1	ENCODE	encode	exon	yes
rs6694256	EFNA1	CANCER		exon	yes
rs12904	EFNA1	CANCER		exon	
rs4255378	EFNA1	CANCER		exon	
rs9297	EFNA1	CANCER		exon	
rs3744262	EFNB3	CANCER		exon	
rs7141	EFNB3	CANCER		exon	
rs2237051	EGF	CANCER		exon	
rs4444903	EGF	CANCER		exon	
rs4698803	EGF	CANCER		exon	
rs3733625	EGF	CANCER		exon	
rs10228436	EGFR	CANCER		exon	
rs10251977	EGFR	CANCER		exon	
rs10277413	EGFR	CANCER		exon	
rs2072454	EGFR	CANCER		exon	
rs2072454	EGFR	ENCODE	disease	exon	
rs11543848	EGFR	ENCODE	disease	exon	

rs	Gene	Panel	Origin	Intron/Exon	Expressed
rs10228436	EGFR	ENCODE	disease	exon	
rs10251977	EGFR	ENCODE	disease	exon	
rs17518376	EGFR	ENCODE	disease	exon	
rs2293347	EGFR	ENCODE	disease	exon	
rs4947963	EGFR	ENCODE	disease	intron	yes
rs1211284	EHD1	ENCODE	encode	exon	yes
rs10897533	EHD1	ENCODE	encode	exon	yes
rs12573	EHD1	ENCODE	encode	exon	yes
rs7126744	EHD1	ENCODE	encode	intron	
rs9466	EIF3S6IP	ENCODE	chr22	exon	yes
rs2157472	EIF3S6IP	ENCODE	chr22	intron	yes
rs5753627	EIF4ENIF1	ENCODE	encode+chr22	exon	yes
rs4820972	EIF4ENIF1	ENCODE	encode+chr22	intron	yes
rs2227997	ELK1	CANCER		exon	
rs2302901	ELK3	CANCER		exon	
rs1043327	ELL	CANCER		exon	
rs2277531	ELL3	ENCODE	encode	exon	
rs3844075	ELL3	ENCODE	encode	intron	yes
rs9537	EMID1	ENCODE	chr22	exon	
rs132369	EMID1	ENCODE	chr22	intron	
rs2482	EMR3	CANCER		exon	
rs300239	ENC1	CANCER		exon	yes
rs442425	ENC1	CANCER		exon	yes
rs300259	ENC1	CANCER		exon	
rs1046088	EP300	ENCODE	chr22	exon	yes
rs2267424	EP300	ENCODE	chr22	intron	yes
rs10952549	EPHA1	CANCER		exon	
rs1131883	EPHA1	CANCER		exon	
rs6678618	EPHA2	CANCER		exon	
rs3762717	EPHA3	CANCER		exon	
rs7650466	EPHA3	CANCER		exon	
rs7349683	EPHA5	CANCER		exon	
rs345730	EPHA7	CANCER		exon	
rs2278107	EPHA7	CANCER		exon	
rs209696	EPHA8	CANCER		exon	
rs209698	EPHA8	CANCER		exon	
rs999765	EPHA8	CANCER		exon	
rs3732566	EPHB1	CANCER		exon	
rs7644369	EPHB1	CANCER		exon	
rs2295022	EPHB2	CANCER		exon	
rs9862375	EPHB3	CANCER		exon	
rs9881589	EPHB3	CANCER		exon	
rs7652033	EPHB3	CANCER		exon	
rs8177146	EPHB6	CANCER		exon	
rs8177153	EPHB6	CANCER		exon	
rs8177159	EPHB6	CANCER		exon	
rs1051740	EPHX1	CANCER		exon	
rs1051741	EPHX1	CANCER		exon	
rs2292568	EPHX1	CANCER		exon	
rs2235481	EPM2A	CANCER		exon	
rs17567	EPS15	CANCER		exon	yes

rs	Gene	Panel	Origin	Intron/Exon	Expressed
rs7308	EPS15	CANCER		exon	yes
rs8664	EPS8	CANCER		exon	yes
rs1058808	ERBB2	CANCER		exon	
rs773123	ERBB3	CANCER		exon	
rs2229046	ERBB3	CANCER		exon	
rs11615	ERCC1	CANCER		exon	yes
rs1052559	ERCC2	CANCER		exon	yes
rs13181	ERCC2	CANCER		exon	yes
rs4150521	ERCC3	CANCER		exon	
rs1799801	ERCC4	CANCER		exon	
rs17655	ERCC5	CANCER		exon	yes
rs2227869	ERCC5	CANCER		exon	yes
rs1047769	ERCC5	CANCER		exon	
rs2228528	ERCC6	CANCER		exon	yes
rs2229760	ERCC6	CANCER		exon	yes
rs2228524	ERCC6	CANCER		exon	
rs2836441	ERG	CANCER		exon	
rs2836441	ERG	ENCODE	chr21	exon	
rs17230484	ERG	ENCODE	chr21	exon	
rs3088093	ERN1	CANCER		exon	yes
rs196912	ERN1	CANCER		exon	
rs2077647	ESR1	CANCER		exon	
rs3798577	ESR1	CANCER		exon	
rs1801132	ESR1	CANCER		exon	
rs4986934	ESR1	CANCER		exon	
rs4937333	ETS1	CANCER		exon	yes
rs8705	ETS1	CANCER		exon	yes
rs1051420	ETS2	CANCER		exon	yes
rs11254	ETS2	CANCER		exon	yes
rs461155	ETS2	ENCODE	chr21	exon	yes
rs1209953	ETS2	ENCODE	chr21	intron	yes
rs3823702	ETV1	CANCER		exon	
rs9639168	ETV1	CANCER		exon	
rs1058028	ETV6	CANCER		exon	yes
rs7505	EVI2A	CANCER		exon	yes
rs6953296	EXOC4	ENCODE	disease	exon	
rs2042456	EXOC4	ENCODE	disease	exon	yes
rs7790835	EXOC4	ENCODE	disease	intron	
rs3734279	EYA4	CANCER		exon	
rs9493627	EYA4	CANCER		exon	
rs5960	F10	ENCODE	encode	exon	
rs3211719	F10	ENCODE	encode	intron	
rs1801719	F2R	CANCER		exon	yes
rs2227800	F2R	CANCER		exon	
rs2230849	F2R	CANCER		exon	
rs6046	F7	ENCODE	encode	exon	
rs6042	F7	ENCODE	encode	exon	
rs1475931	F7	ENCODE	encode	intron	
rs1800291	F8	ENCODE	encode	exon	yes
rs1050705	F8	ENCODE	encode	exon	yes
rs17281377	F8	ENCODE	encode	intron	

rs	Gene	Panel	Origin	Intron/Exon	Expressed
rs16999837	FAM19A5	ENCODE	chr22	exon	
rs17764077	FAM19A5	ENCODE	chr22	intron	
rs757368	FAM3B	ENCODE	chr21	exon	
rs2838012	FAM3B	ENCODE	chr21	exon	
rs12627067	FAM3B	ENCODE	chr21	intron	
rs11914082	FAM83F	ENCODE	chr22	exon	
rs2958651	FAM83F	ENCODE	chr22	exon	
rs17406434	FAM83F	ENCODE	chr22	intron	
rs11649210	FANCA	CANCER		exon	
rs1800331	FANCA	CANCER		exon	
rs2239359	FANCA	CANCER		exon	
rs7647987	FANCD2	CANCER		exon	yes
rs7626117	FANCD2	CANCER		exon	
rs7761870	FANCE	CANCER		exon	
rs4447177	FANCF	CANCER		exon	yes
rs10500938	FANCF	CANCER		exon	
rs848291	FANCL	CANCER		exon	yes
rs1468063	FAS	CANCER		exon	yes
rs3218612	FAS	CANCER		exon	
rs9658776	FAS	CANCER		exon	
rs2288648	FASTK	CANCER		exon	
rs1280099	FAT	CANCER		exon	
rs2637777	FAT	CANCER		exon	
rs458021	FAT	CANCER		exon	
rs7381	FBLN1	ENCODE	chr22	exon	
rs9682	FBLN1	ENCODE	chr22	exon	
rs6007065	FBLN1	ENCODE	chr22	intron	
rs710174	FBXO7	ENCODE	encode+chr22	exon	yes
rs8140067	FBXO7	ENCODE	encode+chr22	intron	yes
rs10917661	FCGR2B	ENCODE	disease	exon	
rs6681090	FCGR2B	ENCODE	disease	exon	
rs7522061	FCRL3	ENCODE	disease	exon	
rs2282284	FCRL3	ENCODE	disease	exon	
rs6691569	FCRL3	ENCODE	disease	exon	yes
rs11264793	FCRL3	ENCODE	disease	intron	yes
rs4246215	FEN1	CANCER		exon	yes
rs412334	FEN1	CANCER		exon	
rs1133392	FER	CANCER		exon	yes
rs2229085	FER	CANCER		exon	yes
rs919771	FER	CANCER		exon	yes
rs2227989	FES	CANCER		exon	
rs34000	FGF1	CANCER		exon	
rs34001	FGF1	CANCER		exon	
rs34002	FGF1	CANCER		exon	
rs33999	FGF1	ENCODE	encode	exon	
rs1460922	FGF12	CANCER		exon	
rs1460923	FGF12	CANCER		exon	
rs1460924	FGF12	CANCER		exon	
rs1476217	FGF2	CANCER		exon	yes
rs3747676	FGF2	CANCER		exon	yes
rs3804158	FGF2	CANCER		exon	

rs	Gene	Panel	Origin	Intron/Exon	Expressed
rs3793405	FGF20	ENCODE	disease	exon	
rs6991982	FGF20	ENCODE	disease	intron	
rs3733336	FGF5	CANCER		exon	
rs2241280	FGF6	CANCER		exon	
rs7358740	FGF6	CANCER		exon	
rs546782	FGF9	CANCER		exon	yes
rs9509841	FGF9	CANCER		exon	
rs2304000	FGFR1	CANCER		exon	
rs1047057	FGFR2	CANCER		exon	
rs1047100	FGFR2	CANCER		exon	
rs1801043	FGFR2	CANCER		exon	
rs4647928	FGFR3	CANCER		exon	
rs1076890	FGFR4	CANCER		exon	yes
rs376618	FGFR4	CANCER		exon	
rs452885	FGFR4	CANCER		exon	
rs9018	FHL1	CANCER		exon	yes
rs9627391	FLJ10945	ENCODE	chr22	exon	
rs8137493	FLJ10945	ENCODE	chr22	intron	
rs17512204	FLJ10996	ENCODE	encode	exon	yes
rs1046330	FLJ10996	ENCODE	encode	exon	yes
rs10490631	FLJ10996	ENCODE	encode	intron	yes
rs1048310	FLJ20232	ENCODE	chr22	exon	yes
rs2232088	FLJ20232	ENCODE	chr22	exon	
rs17400681	FLJ20232	ENCODE	chr22	exon	
rs4821903	FLJ20232	ENCODE	chr22	intron	yes
rs9615938	FLJ20699	ENCODE	chr22	exon	yes
rs6008434	FLJ20699	ENCODE	chr22	intron	
rs1029365	FLJ21062	ENCODE	encode	exon	yes
rs3761805	FLJ21062	ENCODE	encode	exon	yes
rs17862129	FLJ21062	ENCODE	encode	exon	
rs194537	FLJ21062	ENCODE	encode	intron	
rs13057910	FLJ21125	ENCODE	chr22	exon	
rs1053001	FLJ21125	ENCODE	chr22	exon	yes
rs11704009	FLJ21125	ENCODE	chr22	intron	
rs2277841	FLJ23322	ENCODE	chr22	exon	yes
rs3747163	FLJ23322	ENCODE	chr22	exon	yes
rs2281086	FLJ23322	ENCODE	chr22	intron	
rs2839716	FLJ23584	ENCODE	chr22	exon	
rs3747203	FLJ23588	ENCODE	chr22	exon	
rs137794	FLJ23588	ENCODE	chr22	exon	yes
rs137731	FLJ23588	ENCODE	chr22	exon	
rs9614382	FLJ23588	ENCODE	chr22	exon	
rs9614177	FLJ23588	ENCODE	chr22	intron	
rs17319801	FLJ25421	ENCODE	chr22	exon	
rs11704363	FLJ25421	ENCODE	chr22	intron	
rs9603932	FLJ26443	ENCODE	encode	exon	
rs7338610	FLJ26443	ENCODE	encode	exon	
rs4907733	FLJ26443	ENCODE	encode	exon	
rs11620577	FLJ26443	ENCODE	encode	intron	
rs11090909	FLJ27365	ENCODE	chr22	exon	
rs3747243	FLJ27365	ENCODE	chr22	exon	

rs	Gene	Panel	Origin	Intron/Exon	Expressed
rs8135478	FLJ27365	ENCODE	chr22	exon	
rs9626891	FLJ27365	ENCODE	chr22	intron	
rs2003752	FLJ31568	ENCODE	chr22	exon	
rs1807113	FLJ31568	ENCODE	chr22	intron	
rs5762795	FLJ33814	ENCODE	chr22	exon	yes
rs2235432	FLJ33814	ENCODE	chr22	intron	yes
rs2240176	FLJ35801	ENCODE	chr22	exon	yes
rs11089483	FLJ35801	ENCODE	chr22	intron	
rs11705259	FLJ36046	ENCODE	chr22	exon	
rs861854	FLJ36046	ENCODE	chr22	exon	
rs619484	FLJ41733	ENCODE	chr21	exon	
rs137843	FLJ41993	ENCODE	chr22	exon	
rs135793	FLJ44385	ENCODE	chr22	exon	
rs135783	FLJ44385	ENCODE	chr22	intron	
rs9653664	FLJ45139	ENCODE	chr21	exon	
rs414845	FLJ45139	ENCODE	chr21	intron	
rs766425	FLJ46020	ENCODE	chr21	exon	
rs17655956	FLJ46020	ENCODE	chr21	intron	
rs2337970	FLJ46257	ENCODE	chr22	exon	
rs877528	FLJ46257	ENCODE	chr22	exon	
rs4334580	FLJ46257	ENCODE	chr22	exon	
rs9615561	FLJ46257	ENCODE	chr22	intron	
rs2296189	FLT1	CANCER		exon	
rs1933437	FLT3	CANCER		exon	
rs7338903	FLT3	CANCER		exon	
rs307826	FLT4	CANCER		exon	
rs11949194	FLT4	CANCER		exon	
rs1053238	FN1	CANCER		exon	
rs1263	FN1	CANCER		exon	
rs1264	FN1	CANCER		exon	
rs2239615	FOS	CANCER		exon	
rs7101	FOS	CANCER		exon	
rs1049739	FOSB	CANCER		exon	yes
rs1049698	FOSB	CANCER		exon	
rs708905	FOSB	CANCER		exon	
rs2279990	FOSL2	CANCER		exon	
rs9471607	FOXP4	ENCODE	encode	exon	yes
rs9381075	FOXP4	ENCODE	encode	intron	
rs792310	FPGT	CANCER		exon	yes
rs1064261	FRAP1	CANCER		exon	yes
rs1057079	FRAP1	CANCER		exon	
rs11121705	FRAP1	CANCER		exon	
rs495565	FRK	CANCER		exon	yes
rs580396	FRK	CANCER		exon	yes
rs1338507	FRK	CANCER		exon	
rs2306674	FRMD5	ENCODE	encode	exon	
rs12442297	FRMD5	ENCODE	encode	intron	
rs1057227	FRS3	ENCODE	encode	exon	yes
rs3761781	FRS3	ENCODE	encode	intron	
rs13009	FRZB	CANCER		exon	
rs7775	FRZB	CANCER		exon	

rs	Gene	Panel	Origin	Intron/Exon	Expressed
rs3779536	FSCN3	ENCODE	encode	exon	
rs806213	FSCN3	ENCODE	encode	intron	
rs10432965	FTCD	ENCODE	chr21	exon	
rs1980983	FTCD	ENCODE	chr21	intron	
rs6810	FVT1	CANCER		exon	yes
rs1057979	FYN	CANCER		exon	
rs1178947	FZD9	CANCER		exon	
rs1050757	G6PD	CANCER		exon	
rs1050829	G6PD	CANCER		exon	
rs1050757	G6PD	ENCODE	encode	exon	yes
rs743544	G6PD	ENCODE	encode	intron	
rs17281349	GAB3	ENCODE	encode	exon	
rs3813455	GAB3	ENCODE	encode	exon	yes
rs5945109	GAB3	ENCODE	encode	intron	
rs5992604	GAB4	ENCODE	chr22	exon	
rs5992598	GAB4	ENCODE	chr22	exon	
rs5748765	GAB4	ENCODE	chr22	intron	
rs140685	GABRA5	CANCER		exon	
rs3219151	GABRA6	ENCODE	disease	exon	
rs13184586	GABRA6	ENCODE	disease	exon	
rs7704209	GABRA6	ENCODE	disease	intron	
rs25409	GABRB3	CANCER		exon	
rs140679	GABRG3	CANCER		exon	
rs2267161	GAL3ST1	ENCODE	chr22	exon	
rs4149490	GAL3ST1	ENCODE	chr22	intron	
rs5371	GALR1	CANCER		exon	
rs5374	GALR1	CANCER		exon	
rs5376	GALR1	CANCER		exon	
rs9984077	GART	ENCODE	encode+chr21	exon	yes
rs8971	GART	ENCODE	encode+chr21	exon	yes
rs7283354	GART	ENCODE	encode+chr21	intron	yes
rs1047365	GAS7	CANCER		exon	yes
rs2240739	GAS7	CANCER		exon	yes
rs2270121	GAS7	CANCER		exon	yes
rs13911	GCAT	ENCODE	chr22	exon	yes
rs710187	GCAT	ENCODE	chr22	exon	
rs2071910	GCAT	ENCODE	chr22	intron	
rs989099	GCC1	ENCODE	encode	exon	yes
rs3007	GDF10	CANCER		exon	
rs9860	GDF10	CANCER		exon	
rs143384	GDF5	ENCODE	encode	exon	
rs224330	GDF5	ENCODE	encode	exon	
rs224332	GDF5	ENCODE	encode	intron	
rs10491279	GDF9	ENCODE	encode	exon	
rs1325432	GFI1	CANCER		exon	yes
rs4970714	GFI1	CANCER		exon	yes
rs2667	GFPT1	ENCODE	disease	exon	yes
rs12473304	GFPT1	ENCODE	disease	intron	yes
rs2269547	GGA1	ENCODE	chr22	exon	yes
rs6000842	GGA1	ENCODE	chr22	intron	
rs7288201	GGTLA1	ENCODE	chr22	exon	

rs	Gene	Panel	Origin	Intron/Exon	Expressed
rs2274083	GJB2	CANCER		exon	
rs2274084	GJB2	CANCER		exon	
rs3751385	GJB2	CANCER		exon	
rs7305145	GLI1	CANCER		exon	
rs7973381	GLI1	CANCER		exon	
rs3738880	GLI2	CANCER		exon	
rs719337	GMPPA	ENCODE	encode	exon	
rs2276640	GMPPA	ENCODE	encode	intron	
rs1062597	GNA13	CANCER		exon	
rs7121	GNAS	CANCER		exon	yes
rs15952	GNAS	CANCER		exon	
rs3730171	GNAS	CANCER		exon	
rs9612234	GNAZ	ENCODE	chr22	exon	yes
rs1805058	GNAZ	ENCODE	chr22	exon	
rs3788339	GNAZ	ENCODE	chr22	intron	
rs3752174	GNG7	CANCER		exon	yes
rs3792215	GPC1	CANCER		exon	
rs3792216	GPC1	CANCER		exon	
rs571247	GPR116	CANCER		exon	
rs678312	GPR116	CANCER		exon	
rs133074	GPR24	ENCODE	chr22	exon	
rs1800668	GPX1	CANCER		exon	
rs11548	GPX3	CANCER		exon	
rs2070593	GPX3	CANCER		exon	
rs2230303	GPX3	CANCER		exon	
rs3807551	GRB10	CANCER		exon	
rs363538	GRIK1	ENCODE	chr21	exon	
rs363430	GRIK1	ENCODE	chr21	exon	
rs363504	GRIK1	ENCODE	chr21	exon	
rs363501	GRIK1	ENCODE	chr21	intron	
rs2073549	GRM3	ENCODE	disease	exon	
rs701332	GRM3	ENCODE	disease	intron	
rs712723	GRM8	ENCODE	encode	exon	yes
rs6976644	GRM8	ENCODE	encode	intron	
rs4986945	GRPR	CANCER		exon	
rs4986946	GRPR	CANCER		exon	
rs2227958	GSTM1	CANCER		exon	
rs1332018	GSTM3	ENCODE	disease	exon	
rs1109138	GSTM3	ENCODE	disease	exon	
rs1571858	GSTM3	ENCODE	disease	intron	yes
rs4891	GSTP1	CANCER		exon	yes
rs947894	GSTP1	CANCER		exon	yes
rs6008729	GTSE1	ENCODE	chr22	exon	yes
rs140054	GTSE1	ENCODE	chr22	exon	yes
rs9615947	GTSE1	ENCODE	chr22	exon	yes
rs6008622	GTSE1	ENCODE	chr22	exon	yes
rs9615344	GTSE1	ENCODE	chr22	intron	
rs2816	GUCY2D	CANCER		exon	
rs3829789	GUCY2D	CANCER		exon	
rs494589	GUCY2F	CANCER		exon	
rs9530	GUSB	CANCER		exon	yes

rs	Gene	Panel	Origin	Intron/Exon	Expressed
rs6000898	H1F0	ENCODE	chr22	exon	
rs13385	HBEGF	CANCER		exon	yes
rs7268	HBEGF	CANCER		exon	yes
rs4150238	HBEGF	CANCER		exon	
rs2782	hCAP-H2	ENCODE	chr22	exon	
rs6010121	hCAP-H2	ENCODE	chr22	intron	
rs3796088	HCG3	ENCODE	encode	exon	
rs362303	HD	ENCODE	disease	exon	yes
rs362272	HD	ENCODE	disease	exon	yes
rs2276881	HD	ENCODE	disease	exon	yes
rs362331	HD	ENCODE	disease	exon	yes
rs363125	HD	ENCODE	disease	exon	yes
rs363075	HD	ENCODE	disease	exon	
rs10009935	HD	ENCODE	disease	intron	
rs375171	HDAC5	CANCER		exon	
rs1044138	HDAC5	CANCER		exon	
rs1127346	HDAC6	CANCER		exon	
rs2408874	HDAC7A	CANCER		exon	yes
rs7418	HDAC7A	CANCER		exon	yes
rs7306788	HDAC7A	CANCER		exon	
rs2023938	HDAC9	CANCER		exon	yes
rs801524	HDAC9	CANCER		exon	yes
rs2074633	HDAC9	CANCER		exon	
rs9842	HDGF	CANCER		exon	yes
rs1045537	HFE	CANCER		exon	
rs1799945	HFE	CANCER		exon	
rs5745687	HGF	CANCER		exon	
rs11727676	HHIP	CANCER		exon	
rs12507427	HHIP	CANCER		exon	
rs3747086	HIC2	CANCER		exon	
rs464694	HIC2	CANCER		exon	
rs11549465	HIF1A	CANCER		exon	yes
rs689797	HISPPD2A	ENCODE	encode	exon	yes
rs2255663	HISPPD2A	ENCODE	encode	intron	
rs11244	HLA	CANCER		exon	yes
rs16871435	HLA	CANCER		exon	yes
rs2070121	HLA	CANCER		exon	yes
rs7194	HLA	CANCER		exon	yes
rs7905	HLA	CANCER		exon	yes
rs8084	HLA	CANCER		exon	yes
rs8807	HLA	CANCER		exon	yes
rs9273960	HLA	CANCER		exon	yes
rs9277534	HLA	CANCER		exon	yes
rs9277535	HLA	CANCER		exon	yes
rs2071470	HLA	CANCER		exon	
rs1042190	HLA	CANCER		exon	
rs9272934	HLA	CANCER		exon	
rs9469266	HLA	CANCER		exon	
rs17841951	HLA	CANCER		exon	
rs1065758	HLCS	ENCODE	chr21	exon	
rs2845804	HLCS	ENCODE	chr21	intron	

rs	Gene	Panel	Origin	Intron/Exon	Expressed
rs12940636	HLF	CANCER		exon	
rs17746075	HLF	CANCER		exon	
rs1474557	HMG2L1	ENCODE	chr22	exon	
rs1053593	HMG2L1	ENCODE	chr22	exon	yes
rs2235145	HMG2L1	ENCODE	chr22	intron	
rs299290	HMMR	CANCER		exon	yes
rs1800961	HNF4A	ENCODE	disease	exon	
rs736823	HNF4A	ENCODE	disease	exon	
rs11086926	HNF4A	ENCODE	disease	exon	
rs6031546	HNF4A	ENCODE	disease	intron	
rs4245861	HNMT	ENCODE	disease	exon	
rs17759	HNRPAB	CANCER		exon	yes
rs6461992	HOXA11	ENCODE	encode	exon	
rs6968828	HOXA11	ENCODE	encode	intron	
rs1989935	HOXA3	ENCODE	encode	exon	
rs2301720	HOXA7	ENCODE	encode	exon	
rs10259620	HOXA9	CANCER		exon	
rs10259620	HOXA9	ENCODE	encode	exon	
rs8556	HOXB13	CANCER		exon	
rs9900627	HOXB13	CANCER		exon	
rs1249077	HOXC6	CANCER		exon	
rs1042328	HPN	CANCER		exon	
rs1688029	HPN	CANCER		exon	
rs3747134	HPS4	ENCODE	chr22	exon	yes
rs3747129	HPS4	ENCODE	chr22	exon	yes
rs2014410	HPS4	ENCODE	chr22	exon	
rs3752589	HPS4	ENCODE	chr22	exon	yes
rs722997	HPS4	ENCODE	chr22	intron	yes
rs11099592	HPSE	CANCER		exon	
rs3747011	HRMT1L1	ENCODE	chr21	exon	yes
rs1054028	HS3ST2	CANCER		exon	
rs208951	HS3ST2	CANCER		exon	
rs3803711	HS3ST4	ENCODE	encode	exon	
rs2943332	HS3ST4	ENCODE	encode	intron	
rs9625679	HS747E2A	ENCODE	chr22	exon	
rs1061810	HSD17B12	CANCER		exon	yes
rs2838343	HSF2BP	ENCODE	chr21	exon	
rs162398	HSF2BP	ENCODE	chr21	exon	
rs1063391	HSPA2	CANCER		exon	
rs5749426	HSPC117	ENCODE	encode+chr22	exon	yes
rs12530	HSPC117	ENCODE	encode+chr22	exon	
rs12158923	HSPC117	ENCODE	encode+chr22	intron	
rs1048659	HTF9C	ENCODE	chr22	exon	
rs1633445	HTF9C	ENCODE	chr22	intron	yes
rs6296	HTR1B	CANCER		exon	
rs6298	HTR1B	CANCER		exon	
rs3125	HTR2A	CANCER		exon	
rs6313	HTR2A	CANCER		exon	
rs6314	HTR2A	CANCER		exon	
rs2833598	HUNK	ENCODE	chr21	exon	
rs8128071	HUNK	ENCODE	chr21	intron	

rs	Gene	Panel	Origin	Intron/Exon	Expressed
rs1056007	IAPP	CANCER		exon	
rs5488	IAPP	CANCER		exon	
rs5484	IAPP	CANCER		exon	
rs3093032	ICAM1	CANCER		exon	yes
rs5490	ICAM1	CANCER		exon	
rs5491	ICAM1	CANCER		exon	
rs4819388	ICOSLG	ENCODE	chr21	exon	yes
rs3746963	ICOSLG	ENCODE	chr21	intron	yes
rs1802548	ID1	CANCER		exon	
rs8116155	ID1	CANCER		exon	
rs2834202	IFNAR1	ENCODE	encode+chr21	exon	yes
rs2257167	IFNAR1	ENCODE	encode+chr21	exon	yes
rs2856968	IFNAR1	ENCODE	encode+chr21	intron	yes
rs4986956	IFNAR2	ENCODE	encode+chr21	exon	yes
rs11088247	IFNAR2	ENCODE	encode+chr21	intron	yes
rs11914	IFNGR1	CANCER		exon	yes
rs1059293	IFNGR2	CANCER		exon	yes
rs6214	IGF1	CANCER		exon	yes
rs6219	IGF1	CANCER		exon	yes
rs6217	IGF1	CANCER		exon	
rs2228531	IGF1R	CANCER		exon	
rs2229765	IGF1R	CANCER		exon	
rs3743262	IGF1R	CANCER		exon	
rs3213234	IGF2	CANCER		exon	
rs1003483	IGF2AS	CANCER		exon	
rs1003483	IGF2AS	ENCODE	encode	exon	
rs4244808	IGF2AS	ENCODE	encode	intron	
rs998075	IGF2R	CANCER		exon	yes
rs8191886	IGF2R	CANCER		exon	
rs8191842	IGF2R	CANCER		exon	
rs4619	IGFBP1	CANCER		exon	
rs4988515	IGFBP1	CANCER		exon	
rs6670	IGFBP3	CANCER		exon	yes
rs1052946	IGFBP5	CANCER		exon	
rs11575213	IGFBP5	CANCER		exon	
rs3276	IGFBP5	CANCER		exon	
rs8141940	IGLL1	ENCODE	chr22	exon	
rs4445669	IGSF4	CANCER		exon	yes
rs3731881	IHH	CANCER		exon	
rs3024496	IL10	CANCER		exon	yes
rs3024498	IL10	CANCER		exon	yes
rs3024510	IL10	CANCER		exon	
rs3024498	IL10	ENCODE	disease	exon	yes
rs3024495	IL10	ENCODE	disease	intron	
rs3171425	IL10RB	ENCODE	encode+chr21	exon	
rs2834167	IL10RB	ENCODE	encode+chr21	exon	
rs2284552	IL10RB	ENCODE	encode+chr21	intron	yes
rs1042505	IL11	CANCER		exon	
rs1042506	IL11	CANCER		exon	
rs568408	IL12A	CANCER		exon	yes
rs1368439	IL12B	CANCER		exon	yes

rs	Gene	Panel	Origin	Intron/Exon	Expressed
rs3213119	IL12B	ENCODE	disease	exon	
rs1368439	IL12B	ENCODE	disease	exon	yes
rs2853697	IL12B	ENCODE	disease	intron	
rs20541	IL13	CANCER		exon	
rs848	IL13	CANCER		exon	
rs20541	IL13	ENCODE	disease+encode	exon	
rs1295686	IL13	ENCODE	disease+encode	intron	
rs1131445	IL16	CANCER		exon	yes
rs3726	IL16	CANCER		exon	yes
rs859	IL16	CANCER		exon	yes
rs879576	IL17R	ENCODE	chr22	exon	
rs1025689	IL17RB	CANCER		exon	yes
rs1043261	IL17RB	CANCER		exon	yes
rs6798958	IL17RB	CANCER		exon	
rs949323	IL18BP	CANCER		exon	yes
rs1304037	IL1A	CANCER		exon	yes
rs17561	IL1A	CANCER		exon	yes
rs1071676	IL1B	CANCER		exon	yes
rs16062	IL1B	CANCER		exon	
rs315951	IL1RN	CANCER		exon	
rs2069763	IL2	CANCER		exon	
rs3087209	IL2	CANCER		exon	
rs3218367	IL2RB	ENCODE	chr22	exon	
rs84458	IL2RB	ENCODE	chr22	intron	
rs40401	IL3	CANCER		exon	
rs40401	IL3	ENCODE	encode	exon	
rs31481	IL3	ENCODE	encode	intron	
rs2070874	IL4	CANCER		exon	
rs2070874	IL4	ENCODE	disease+encode	exon	
rs2227545	IL8	CANCER		exon	
rs1043388	ILK	CANCER		exon	yes
rs1043390	ILK	CANCER		exon	yes
rs2292195	ILK	CANCER		exon	yes
rs1053474	IMPACT	CANCER		exon	yes
rs677688	IMPACT	CANCER		exon	yes
rs1053480	IMPACT	CANCER		exon	
rs1061386	ING1	CANCER		exon	yes
rs1441043	ING1	CANCER		exon	
rs2962	INSR	CANCER		exon	yes
rs6413501	INSR	CANCER		exon	
rs7258741	INSR	CANCER		exon	
rs1059701	IRAK1	CANCER		exon	yes
rs11465988	IRAK3	CANCER		exon	
rs4251545	IRAK4	CANCER		exon	yes
rs17622685	IRF1	ENCODE	disease+encode	exon	
rs1061501	IRF7	CANCER		exon	
rs1061502	IRF7	CANCER		exon	
rs2289046	IRS2	CANCER		exon	yes
rs2289047	IRS2	CANCER		exon	yes
rs1865434	IRS2	CANCER		exon	
rs1017	ISL1	CANCER		exon	

rs	Gene	Panel	Origin	Intron/Exon	Expressed
rs1109527	ITGA2	CANCER		exon	
rs3212651	ITGA2	CANCER		exon	
rs5912	ITGA2B	ENCODE	disease	exon	
rs9890900	ITGA2B	ENCODE	disease	intron	
rs2293649	ITGA6	CANCER		exon	
rs1316757	ITGB1	CANCER		exon	yes
rs10593	ITGB1BP1	CANCER		exon	yes
rs4798	ITGB1BP1	CANCER		exon	yes
rs235326	ITGB2	ENCODE	chr21	exon	yes
rs684	ITGB2	ENCODE	chr21	exon	yes
rs180318	ITGB2	ENCODE	chr21	intron	
rs9367	ITGB4	CANCER		exon	
rs27988	ITK	CANCER		exon	
rs3892245	ITK	CANCER		exon	
rs1900941	ITPR2	CANCER		exon	yes
rs2291264	ITPR2	CANCER		exon	yes
rs2230375	ITPR2	CANCER		exon	
rs2229634	ITPR3	CANCER		exon	yes
rs749338	ITPR3	CANCER		exon	yes
rs7765820	ITPR3	CANCER		exon	
rs1048672	ITSN1	ENCODE	chr21	exon	
rs1108000	ITSN1	ENCODE	chr21	exon	
rs1892700	ITSN1	ENCODE	chr21	intron	
rs7828	JAG1	CANCER		exon	yes
rs3742943	JAG2	CANCER		exon	
rs2230724	JAK2	CANCER		exon	yes
rs7048717	JAK2	CANCER		exon	yes
rs2274472	JAK2	CANCER		exon	
rs3008	JAK3	CANCER		exon	yes
rs2829877	JAM2	ENCODE	chr21	exon	yes
rs4816260	JAM2	ENCODE	chr21	intron	yes
rs4980809	JARID1A	CANCER		exon	yes
rs3827358	JOSD1	ENCODE	chr22	exon	yes
rs4820345	JOSD1	ENCODE	chr22	intron	yes
rs4647018	JUN	CANCER		exon	
rs1061595	JUNB	CANCER		exon	
rs16982300	JUND	CANCER		exon	
rs10478	KATNAL1	ENCODE	encode	exon	yes
rs9508679	KATNAL1	ENCODE	encode	intron	
rs11702354	KCNE1	ENCODE	chr21	exon	
rs2834502	KCNE1	ENCODE	chr21	exon	
rs2834485	KCNE1	ENCODE	chr21	exon	
rs2070358	KCNE1	ENCODE	chr21	intron	
rs2230033	KCNJ15	ENCODE	chr21	exon	yes
rs2836266	KCNJ15	ENCODE	chr21	intron	
rs2070995	KCNJ6	ENCODE	chr21	exon	
rs702859	KCNJ6	ENCODE	chr21	exon	
rs857979	KCNJ6	ENCODE	chr21	intron	
rs10798	KCNQ1	CANCER		exon	yes
rs8234	KCNQ1	CANCER		exon	
rs2519184	KCNQ1	CANCER		exon	

rs	Gene	Panel	Origin	Intron/Exon	Expressed
rs12662818	KCNQ5	ENCODE	encode	exon	
rs1543621	KCNQ5	ENCODE	encode	intron	yes
rs2305948	KDR	CANCER		exon	
rs2269687	KIAA0125	CANCER		exon	
rs7279959	KIAA0179	ENCODE	chr21	exon	
rs7282122	KIAA0179	ENCODE	chr21	intron	yes
rs3747113	KIAA0376	ENCODE	chr22	exon	yes
rs1009668	KIAA0792	CANCER		exon	
rs2292561	KIAA0792	CANCER		exon	
rs2740174	KIAA0792	CANCER		exon	
rs1665105	KIAA0828	CANCER		exon	yes
rs4731575	KIAA0828	CANCER		exon	yes
rs4728164	KIAA0828	CANCER		exon	
rs12225230	KIAA0999	ENCODE	encode	exon	yes
rs10047459	KIAA0999	ENCODE	encode	intron	yes
rs1402815	KIAA1804	CANCER		exon	
rs3795374	KIAA1804	CANCER		exon	
rs963982	KIAA1804	CANCER		exon	
rs7289607	KIAA1904	ENCODE	chr22	exon	
rs11242126	KIF3A	ENCODE	encode	exon	yes
rs17623617	KIF3A	ENCODE	encode	intron	
rs4806591	KIR2DS4	ENCODE	encode	exon	
rs4806583	KIR2DS4	ENCODE	encode	intron	
rs17699088	KIR3DP1	ENCODE	encode	exon	
rs11671355	KIR3DP1	ENCODE	encode	intron	
rs2213181	KIT	CANCER		exon	
rs8022	KIT	CANCER		exon	
rs140519	KLHDC7B	ENCODE	chr22	exon	yes
rs8353	KLHL22	ENCODE	chr22	exon	yes
rs8142474	KLHL22	ENCODE	chr22	intron	
rs1061368	KLK10	CANCER		exon	
rs2569454	KLK10	CANCER		exon	
rs10426	KLK10	CANCER		exon	
rs1048328	KLK11	CANCER		exon	
rs1048344	KLK11	CANCER		exon	
rs2288892	KLK11	CANCER		exon	
rs1058205	KLK3	ENCODE	disease	exon	
rs17632542	KLK3	ENCODE	disease	exon	
rs2003783	KLK3	ENCODE	disease	exon	
rs2659122	KLK3	ENCODE	disease	exon	
rs2292186	KLK3	ENCODE	disease	intron	
rs1137282	KRAS	CANCER		exon	yes
rs12587	KRAS	CANCER		exon	yes
rs13096	KRAS	CANCER		exon	yes
rs9266	KRAS	CANCER		exon	yes
rs713924	KREMEN1	ENCODE	chr22	exon	
rs14024	KRT1	CANCER		exon	
rs903	KRT13	CANCER		exon	
rs4761924	KRT5	CANCER		exon	
rs6603	KRT5	CANCER		exon	
rs233315	KRTAP10-1	ENCODE	chr21	exon	

rs	Gene	Panel	Origin	Intron/Exon	Expressed
rs2329834	KRTAP10-2	ENCODE	chr21	exon	
rs370092	KRTAP10-7	ENCODE	chr21	exon	
rs411254	KRTAP10-8	ENCODE	chr21	exon	
rs9636845	KRTAP11-1	ENCODE	chr21	exon	
rs9306112	KRTAP12-3	ENCODE	chr21	exon	
rs9984726	KRTAP12-4	ENCODE	chr21	exon	
rs877346	KRTAP13-2	ENCODE	chr21	exon	
rs999597	KRTAP13-4	ENCODE	chr21	exon	
rs1023364	KRTAP19-6	ENCODE	chr21	exon	
rs2236427	KRTAP26-1	ENCODE	chr21	exon	
rs1047265	LAF4	CANCER		exon	
rs3786640	LAIR1	ENCODE	encode	exon	
rs4806752	LAIR1	ENCODE	encode	intron	
rs2287828	LAIR2	ENCODE	encode	exon	
rs6509880	LAIR2	ENCODE	encode	intron	
rs7561	LAMB1	CANCER		exon	yes
rs2070917	LAMB1	CANCER		exon	
rs1547715	LAMC1	CANCER		exon	
rs20559	LAMC1	CANCER		exon	
rs3738829	LAMC1	CANCER		exon	
rs86487	LARGE	ENCODE	chr22	exon	yes
rs1046166	LARGE	ENCODE	chr22	exon	
rs16992034	LARGE	ENCODE	chr22	exon	yes
rs2413177	LARGE	ENCODE	chr22	intron	
rs1802141	LAT	CANCER		exon	
rs9937462	LAT	CANCER		exon	
rs6519877	LDOC1L	ENCODE	chr22	exon	
rs708459	LEAP-2	ENCODE	encode	exon	yes
rs183716	LENG4	ENCODE	encode	exon	
rs1050527	LENG4	ENCODE	encode	exon	yes
rs3745439	LENG9	ENCODE	encode	exon	
rs3753082	LIF	CANCER		exon	
rs929271	LIF	ENCODE	chr22	exon	
rs737921	LIF	ENCODE	chr22	intron	
rs20579	LIG1	CANCER		exon	
rs20581	LIG1	CANCER		exon	
rs20580	LIG1	CANCER		exon	
rs1052536	LIG3	CANCER		exon	
rs10131	LIG4	CANCER		exon	yes
rs1805388	LIG4	CANCER		exon	yes
rs1805386	LIG4	CANCER		exon	
rs1052120	LILRA2	ENCODE	encode	exon	
rs10407899	LILRA2	ENCODE	encode	intron	
rs10419832	LILRA4	ENCODE	encode	exon	
rs7253755	LILRA4	ENCODE	encode	intron	
rs8101605	LILRB1	ENCODE	encode	exon	yes
rs3745410	LILRB3	ENCODE	encode	exon	yes
rs3745871	LILRB4	ENCODE	encode	exon	
rs17207369	LILRP2	ENCODE	encode	exon	yes
rs7249176	LILRP2	ENCODE	encode	intron	
rs4141404	LIMK2	CANCER		exon	

rs	Gene	Panel	Origin	Intron/Exon	Expressed
rs4141405	LIMK2	CANCER		exon	
rs2073859	LIMK2	CANCER		exon	
rs4141404	LIMK2	ENCODE	chr22	exon	yes
rs4141405	LIMK2	ENCODE	chr22	exon	
rs6074	LIPC	ENCODE	disease	exon	
rs3829461	LIPC	ENCODE	disease	exon	
rs6083	LIPC	ENCODE	disease	exon	
rs8192701	LIPC	ENCODE	disease	intron	
rs7278737	LIPI	ENCODE	chr21	exon	
rs1556276	LIPI	ENCODE	chr21	intron	
rs1051643	LMNB1	CANCER		exon	yes
rs3749830	LMNB1	CANCER		exon	yes
rs2071458	LMO1	CANCER		exon	
rs3750952	LMO1	CANCER		exon	
rs1042359	LMO1	CANCER		exon	
rs2273799	LMO2	CANCER		exon	
rs3740617	LMO2	CANCER		exon	
rs3740616	LMO2	CANCER		exon	
rs10268268	LMTK2	CANCER		exon	
rs3801294	LMTK2	CANCER		exon	
rs3801296	LMTK2	CANCER		exon	
rs3091367	LOC129138	ENCODE	chr22	exon	
rs2298428	LOC150223	ENCODE	chr22	exon	yes
rs2269729	LOC168850	ENCODE	encode	exon	yes
rs5749088	LOC200312	ENCODE	chr22	exon	
rs757870	LOC200312	ENCODE	chr22	intron	
rs2838389	LOC284837	ENCODE	chr21	exon	
rs6518322	LOC284837	ENCODE	chr21	exon	yes
rs2329577	LOC284837	ENCODE	chr21	intron	
rs916362	LOC348645	ENCODE	chr22	exon	
rs5769707	LOC348645	ENCODE	chr22	exon	
rs135878	LOC348645	ENCODE	chr22	intron	
rs6519442	LOC388882	ENCODE	chr22	exon	
rs17842461	LOC388882	ENCODE	chr22	intron	
rs5768372	LOC388915	ENCODE	chr22	exon	
rs11705447	LOC388915	ENCODE	chr22	exon	
rs6008564	LOC388915	ENCODE	chr22	intron	
rs12168111	LOC400891	ENCODE	chr22	exon	
rs5997219	LOC400891	ENCODE	chr22	intron	
rs41173	LOC400924	ENCODE	chr22	exon	yes
rs1045523	LOC402055	ENCODE	chr22	exon	yes
rs4820682	LOC402055	ENCODE	chr22	exon	yes
rs5761561	LOC402055	ENCODE	chr22	intron	yes
rs17074067	LOC440131	ENCODE	encode	exon	
rs17074065	LOC440131	ENCODE	encode	intron	
rs2522050	LOC441108	ENCODE	encode	exon	yes
rs10071051	LOC441108	ENCODE	encode	intron	yes
rs11702489	LOC441956	ENCODE	chr21	exon	
rs2286442	LOC550631	ENCODE	chr22	exon	yes
rs740223	LOC550631	ENCODE	chr22	exon	
rs737950	LOC550631	ENCODE	chr22	intron	

rs	Gene	Panel	Origin	Intron/Exon	Expressed
rs9607875	LOC91689	ENCODE	chr22	exon	
rs4668123	LRP2	CANCER		exon	
rs831043	LRP2	CANCER		exon	
rs830994	LRP2	CANCER		exon	
rs17616994	LRP5L	ENCODE	chr22	exon	
rs9624807	LRP5L	ENCODE	chr22	exon	
rs9624806	LRP5L	ENCODE	chr22	intron	yes
rs9071	LRRC14	CANCER		exon	yes
rs3781701	LRRC32	CANCER		exon	
rs1048326	LRRK1	CANCER		exon	yes
rs3764739	LRRK1	CANCER		exon	
rs548195	LSP1	ENCODE	encode	exon	yes
rs2089910	LSP1	ENCODE	encode	exon	yes
rs5013907	LSP1	ENCODE	encode	intron	yes
rs2839158	LSS	ENCODE	chr21	exon	yes
rs2254522	LSS	ENCODE	chr21	exon	yes
rs914247	LSS	ENCODE	chr21	exon	yes
rs2968	LSS	ENCODE	chr21	exon	yes
rs2187118	LSS	ENCODE	chr21	intron	
rs2239704	LTA	CANCER		exon	yes
rs2857713	LTA	CANCER		exon	yes
rs3093544	LTA	CANCER		exon	
rs2857713	LTA	ENCODE	disease	exon	yes
rs2224122	LTB4R	CANCER		exon	
rs3742511	LTB4R	CANCER		exon	
rs7026	LU	CANCER		exon	
rs11543125	LYN	CANCER		exon	
rs3747081	LZTR1	ENCODE	chr22	exon	
rs7289487	LZTR1	ENCODE	chr22	intron	yes
rs1801368	MAD1L1	CANCER		exon	
rs2008144	MAGEA1	CANCER		exon	
rs8920	MAGEL2	CANCER		exon	
rs9785	MAGEL2	CANCER		exon	
rs2233070	MAGEL2	CANCER		exon	
rs1059442	MALT1	CANCER		exon	yes
rs689931	MAP1A	ENCODE	encode	exon	
rs523156	MAP1A	ENCODE	encode	intron	
rs4792219	MAP2K4	CANCER		exon	yes
rs2072074	MAP2K6	CANCER		exon	yes
rs832582	MAP3K1	CANCER		exon	yes
rs832583	MAP3K1	CANCER		exon	yes
rs702689	MAP3K1	CANCER		exon	
rs2172710	MAP3K7	CANCER		exon	
rs9451441	MAP3K7	CANCER		exon	
rs1042058	MAP3K8	CANCER		exon	yes
rs8177038	MAP3K8	CANCER		exon	
rs8177039	MAP3K8	CANCER		exon	
rs2286053	MAP3K9	CANCER		exon	
rs3829955	MAP3K9	CANCER		exon	
rs1990032	MAP3K9	CANCER		exon	
rs2276008	MAPK1	ENCODE	chr22	exon	

rs	Gene	Panel	Origin	Intron/Exon	Expressed
rs743411	MAPK1	ENCODE	chr22	intron	
rs958	MAPK10	CANCER		exon	yes
rs2272857	MAPK12	CANCER		exon	
rs2066776	MAPK12	CANCER		exon	
rs2272857	MAPK12	ENCODE	chr22	exon	yes
rs742184	MAPK12	ENCODE	chr22	intron	
rs3804451	MAPK14	CANCER		exon	yes
rs8510	MAPK14	CANCER		exon	yes
rs3288	MAPK4	CANCER		exon	
rs3752086	MAPK4	CANCER		exon	
rs9605	MAPK9	CANCER		exon	yes
rs220721	MAS1	CANCER		exon	
rs13056550	MB	ENCODE	chr22	exon	
rs7292	MB	ENCODE	chr22	exon	
rs1056680	MB	ENCODE	chr22	exon	
rs2076141	MB	ENCODE	chr22	intron	
rs3786254	MBD2	CANCER		exon	yes
rs7614	MBD2	CANCER		exon	yes
rs1259938	MBD2	CANCER		exon	
rs7914	MCAM	CANCER		exon	yes
rs1549181	MCC	CANCER		exon	
rs2227948	MCC	CANCER		exon	
rs3756600	MCC	CANCER		exon	
rs9604022	MCF2L	ENCODE	encode	exon	
rs2281723	MCF2L	ENCODE	encode	exon	
rs7986677	MCF2L	ENCODE	encode	exon	
rs4907574	MCF2L	ENCODE	encode	intron	
rs878471	MCL1	CANCER		exon	yes
rs893293	MCM2	CANCER		exon	
rs9975588	MCM3AP	ENCODE	chr21	exon	yes
rs11702450	MCM3AP	ENCODE	chr21	exon	yes
rs2839181	MCM3AP	ENCODE	chr21	exon	yes
rs2839165	MCM3AP	ENCODE	chr21	intron	yes
rs4645824	MCM5	ENCODE	chr22	exon	yes
rs133417	MCM5	ENCODE	chr22	exon	
rs4645731	MCM5	ENCODE	chr22	intron	
rs3087350	MCM6	CANCER		exon	yes
rs1055521	MDFIC	ENCODE	encode	exon	yes
rs2709507	MDFIC	ENCODE	encode	intron	yes
rs769412	MDM2	CANCER		exon	yes
rs4245739	MDM4	CANCER		exon	yes
rs4252745	MDM4	CANCER		exon	yes
rs1299563	MDS1	CANCER		exon	
rs6796432	MDS1	CANCER		exon	
rs2734647	MECP2	CANCER		exon	yes
rs2734647	MECP2	ENCODE	encode	exon	yes
rs2075596	MECP2	ENCODE	encode	intron	
rs4930276	MEN1	CANCER		exon	
rs607969	MEN1	ENCODE	encode	exon	
rs669976	MEN1	ENCODE	encode	intron	
rs10863	MEST	CANCER		exon	yes

rs	Gene	Panel	Origin	Intron/Exon	Expressed
rs2023748	MET	CANCER		exon	
rs41736	MET	CANCER		exon	
rs2023748	MET	ENCODE	encode	exon	
rs41736	MET	ENCODE	encode	exon	
rs13223756	MET	ENCODE	encode	exon	
rs11762213	MET	ENCODE	encode	exon	
rs38840	MET	ENCODE	encode	intron	
rs2228368	MFAP1	ENCODE	encode	exon	yes
rs16965120	MFAP1	ENCODE	encode	intron	yes
rs1054206	MFAP4	CANCER		exon	
rs11556024	MGC13125	ENCODE	encode	exon	
rs11216129	MGC13125	ENCODE	encode	intron	yes
rs2239961	MGC16703	ENCODE	chr22	intron	
rs4615	MGC17330	ENCODE	chr22	exon	yes
rs4820961	MGC17330	ENCODE	chr22	intron	yes
rs2301521	MGC26816	ENCODE	chr22	exon	
rs133335	MGC26816	ENCODE	chr22	exon	
rs40497	MGC33648	ENCODE	encode	exon	yes
rs2257505	MGC33648	ENCODE	encode	exon	yes
rs33321	MGC33648	ENCODE	encode	intron	yes
rs16989427	MGC50372	ENCODE	encode+chr22	exon	yes
rs1803965	MGMT	CANCER		exon	yes
rs9610875	MICAL-L1	ENCODE	chr22	exon	
rs5750508	MICAL-L1	ENCODE	chr22	intron	
rs4821944	MKL1	ENCODE	chr22	exon	yes
rs8137675	MKL1	ENCODE	chr22	intron	
rs2239669	MKRN3	CANCER		exon	
rs3817373	MLF1	CANCER		exon	
rs1050629	MLF2	CANCER		exon	yes
rs2302371	MLF2	CANCER		exon	yes
rs1800146	MLH1	CANCER		exon	
rs175080	MLH3	CANCER		exon	
rs2071702	MLL	CANCER		exon	
rs9332801	MLL	CANCER		exon	
rs10123995	MLLT3	CANCER		exon	
rs6906754	MLLT4	CANCER		exon	yes
rs1132306	MLLT4	CANCER		exon	
rs3213590	MLLT4	CANCER		exon	
rs12765	MME	CANCER		exon	
rs2118074	MME	CANCER		exon	
rs6665	MME	CANCER		exon	
rs5854	MMP1	CANCER		exon	
rs470558	MMP1	CANCER		exon	
rs2071230	MMP1	CANCER		exon	
rs470168	MMP10	CANCER		exon	
rs486055	MMP10	CANCER		exon	
rs1042704	MMP14	CANCER		exon	
rs2236302	MMP14	CANCER		exon	
rs1042703	MMP14	CANCER		exon	
rs2242295	MMP19	CANCER		exon	
rs7201	MMP2	CANCER		exon	

rs	Gene	Panel	Origin	Intron/Exon	Expressed
rs6060341	MMP24	ENCODE	encode	exon	
rs2425019	MMP24	ENCODE	encode	intron	
rs2499953	MMP26	ENCODE	encode	exon	
rs2442422	MMP26	ENCODE	encode	intron	
rs679620	MMP3	CANCER		exon	
rs10502001	MMP7	CANCER		exon	yes
rs14983	MMP7	CANCER		exon	yes
rs1940475	MMP8	CANCER		exon	
rs2250889	MMP9	CANCER		exon	
rs17476776	MN1	ENCODE	chr22	exon	
rs12233370	MN1	ENCODE	chr22	intron	
rs737924	MORC2	ENCODE	chr22	exon	yes
rs2072132	MORC2	ENCODE	chr22	exon	yes
rs2294396	MOV10L1	ENCODE	chr22	exon	
rs2272838	MOV10L1	ENCODE	chr22	exon	
rs2272836	MOV10L1	ENCODE	chr22	exon	
rs2340601	MOV10L1	ENCODE	chr22	exon	
rs12169319	MOV10L1	ENCODE	chr22	exon	
rs9617066	MOV10L1	ENCODE	chr22	exon	
rs738490	MOV10L1	ENCODE	chr22	intron	
rs3734744	MOXD1	ENCODE	encode	exon	yes
rs15017	MOXD1	ENCODE	encode	exon	yes
rs17704981	MOXD1	ENCODE	encode	exon	
rs710080	MPG	ENCODE	encode	exon	
rs2759	MPO	CANCER		exon	
rs6003217	MPPED1	ENCODE	chr22	exon	
rs2413761	MPPED1	ENCODE	chr22	exon	
rs12484468	MPPED1	ENCODE	chr22	intron	
rs2833761	MRAP	ENCODE	chr21	exon	
rs2155209	MRE11A	CANCER		exon	yes
rs12812	MRPL23	ENCODE	encode	exon	yes
rs1618613	MRPL23	ENCODE	encode	intron	
rs3830160	MRPL28	ENCODE	encode	exon	
rs3178972	MRPL39	ENCODE	chr21	exon	yes
rs1135638	MRPL39	ENCODE	chr21	exon	yes
rs7115	MRPS6	ENCODE	chr21	exon	yes
rs13052524	MRPS6	ENCODE	chr21	intron	yes
rs4987188	MSH2	CANCER		exon	
rs4987189	MSH2	CANCER		exon	
rs4987188	MSH2	ENCODE	disease	exon	
rs1805355	MSH3	CANCER		exon	yes
rs184967	MSH3	CANCER		exon	yes
rs1800935	MSH6	CANCER		exon	yes
rs1042820	MSH6	CANCER		exon	
rs2020908	MSH6	CANCER		exon	
rs1062633	MST1R	CANCER		exon	
rs13815	MT	ENCODE	chr22	exon	yes
rs2066910	MT	ENCODE	chr22	intron	yes
rs1801131	MTHFR	CANCER		exon	yes
rs1537515	MTHFR	CANCER		exon	
rs1801133	MTHFR	CANCER		exon	

rs	Gene	Panel	Origin	Intron/Exon	Expressed
rs4846049	MTHFR	CANCER		exon	
rs16988135	MTMR3	ENCODE	chr22	exon	yes
rs3788418	MTMR3	ENCODE	chr22	intron	yes
rs4509087	MTO1	ENCODE	encode	exon	yes
rs3857463	MTO1	ENCODE	encode	intron	
rs2274419	MUSK	CANCER		exon	
rs578430	MUSK	CANCER		exon	
rs3219484	MUTYH	CANCER		exon	
rs1557370	MX1	ENCODE	disease+chr21	exon	
rs1050008	MX1	ENCODE	disease+chr21	exon	yes
rs2070229	MX1	ENCODE	disease+chr21	exon	yes
rs467960	MX1	ENCODE	disease+chr21	exon	yes
rs461093	MX1	ENCODE	disease+chr21	intron	yes
rs2301803	MX2	ENCODE	chr21	exon	
rs2838027	MX2	ENCODE	chr21	intron	
rs14401	MXI1	CANCER		exon	yes
rs17658	MXI1	CANCER		exon	yes
rs1803997	MXI1	CANCER		exon	
rs285162	MYBL2	CANCER		exon	
rs2070583	MYC	CANCER		exon	yes
rs4645949	MYC	CANCER		exon	
rs922	MYCN	CANCER		exon	
rs1050111	MYH11	CANCER		exon	
rs2075511	MYH11	CANCER		exon	
rs9619601	MYH9	ENCODE	chr22	exon	
rs710181	MYH9	ENCODE	chr22	exon	
rs5756130	MYH9	ENCODE	chr22	exon	yes
rs2481	MYH9	ENCODE	chr22	exon	yes
rs735854	MYH9	ENCODE	chr22	intron	yes
rs2700352	MYLK	CANCER		exon	
rs4678047	MYLK	CANCER		exon	
rs2236005	MYO18B	ENCODE	chr22	exon	
rs5996988	MYO18B	ENCODE	chr22	exon	
rs17704912	MYO18B	ENCODE	chr22	exon	
rs4820658	MYO18B	ENCODE	chr22	exon	
rs738642	MYO18B	ENCODE	chr22	exon	
rs5761170	MYO18B	ENCODE	chr22	exon	
rs3859866	MYO18B	ENCODE	chr22	exon	
rs13058434	MYO18B	ENCODE	chr22	exon	
rs133885	MYO18B	ENCODE	chr22	exon	
rs5761163	MYO18B	ENCODE	chr22	intron	
rs4987076	NAT1	ENCODE	disease	exon	
rs7017402	NAT1	ENCODE	disease	intron	
rs1041983	NAT2	CANCER		exon	
rs1208	NAT2	CANCER		exon	
rs1799929	NAT2	CANCER		exon	
rs1061302	NBS1	CANCER		exon	yes
rs1063045	NBS1	CANCER		exon	yes
rs2826892	NCAM2	ENCODE	chr21	exon	
rs2017705	NCAM2	ENCODE	chr21	exon	
rs6518017	NCAM2	ENCODE	chr21	intron	

rs	Gene	Panel	Origin	Intron/Exon	Expressed
rs2072712	NCF4	ENCODE	chr22	exon	yes
rs4821544	NCF4	ENCODE	chr22	intron	
rs1131171	NCL	CANCER		exon	yes
rs2076546	NCOA3	CANCER		exon	yes
rs2230782	NCOA3	CANCER		exon	yes
rs2076547	NCOA3	CANCER		exon	
rs9394782	NCR2	ENCODE	encode	exon	yes
rs9471575	NCR2	ENCODE	encode	intron	
rs1801311	NDUFA6	ENCODE	chr22	exon	yes
rs7245	NDUFA6	ENCODE	chr22	exon	yes
rs4147641	NDUFA6	ENCODE	chr22	intron	yes
rs4148972	NDUFV3	ENCODE	chr21	exon	yes
rs2839600	NDUFV3	ENCODE	chr21	intron	yes
rs165602	NEFH	ENCODE	chr22	exon	yes
rs165607	NEFH	ENCODE	chr22	intron	
rs1131854	NEO1	CANCER		exon	
rs3736510	NEO1	CANCER		exon	
rs1801052	NF1	CANCER		exon	yes
rs2285892	NF1	CANCER		exon	
rs2230851	NF1	CANCER		exon	
rs1008515	NF2	CANCER		exon	yes
rs1034880	NF2	CANCER		exon	
rs7291645	NF2	CANCER		exon	
rs1008515	NF2	ENCODE	chr22	exon	yes
rs9614004	NF2	ENCODE	chr22	intron	
rs1057157	NFAM1	ENCODE	chr22	exon	yes
rs742157	NFAM1	ENCODE	chr22	intron	
rs1609993	NFKB1	CANCER		exon	yes
rs4648072	NFKB1	CANCER		exon	
rs11574851	NFKB2	CANCER		exon	yes
rs10782383	NFKBIA	CANCER		exon	yes
rs11569620	NFKBIA	CANCER		exon	
rs2273650	NFKBIA	CANCER		exon	
rs1957106	NFKBIA	CANCER		exon	
rs6121023	NFS1	ENCODE	encode	exon	yes
rs6060546	NFS1	ENCODE	encode	intron	
rs6330	NGFB	CANCER		exon	
rs734194	NGFR	CANCER		exon	
rs6002436	NHP2L1	ENCODE	chr22	exon	
rs132806	NHP2L1	ENCODE	chr22	exon	yes
rs3213190	NID	CANCER		exon	yes
rs873	NID	CANCER		exon	
rs2071529	NID	CANCER		exon	
rs7609	NIPSNAP1	ENCODE	chr22	exon	yes
rs469363	NIPSNAP1	ENCODE	chr22	intron	yes
rs2293054	NOS1	ENCODE	disease	exon	
rs9658445	NOS1	ENCODE	disease	exon	
rs3741475	NOS1	ENCODE	disease	exon	
rs9658562	NOS1	ENCODE	disease	exon	
rs9658550	NOS1	ENCODE	disease	intron	
rs1137933	NOS2A	CANCER		exon	

rs	Gene	Panel	Origin	Intron/Exon	Expressed
rs1549758	NOS3	CANCER		exon	
rs3918232	NOS3	ENCODE	disease	exon	
rs1799983	NOS3	ENCODE	disease	exon	
rs1800783	NOS3	ENCODE	disease	intron	
rs6563	NOTCH1	CANCER		exon	
rs699780	NOTCH2	CANCER		exon	yes
rs835575	NOTCH2	CANCER		exon	yes
rs835576	NOTCH2	CANCER		exon	yes
rs699779	NOTCH2	CANCER		exon	
rs1043996	NOTCH3	CANCER		exon	
rs1044006	NOTCH3	CANCER		exon	
rs1044009	NOTCH3	CANCER		exon	
rs423023	NOTCH4	CANCER		exon	
rs915894	NOTCH4	CANCER		exon	
rs4142496	NPR2	CANCER		exon	
rs9785023	NPY	CANCER		exon	
rs1800566	NQO1	CANCER		exon	yes
rs10517	NQO1	CANCER		exon	
rs2288539	NR2F6	CANCER		exon	yes
rs14804	NRAS	CANCER		exon	yes
rs10503929	NRG1	CANCER		exon	
rs7820838	NRG1	CANCER		exon	
rs1056947	NRIP1	ENCODE	chr21	exon	yes
rs3825074	NRXN2	ENCODE	encode	exon	
rs3741399	NRXN2	ENCODE	encode	exon	
rs3802947	NRXN2	ENCODE	encode	intron	
rs6334	NTRK1	CANCER		exon	
rs2289657	NTRK2	CANCER		exon	
rs7167629	NTRK3	CANCER		exon	
rs11698783	NTSR1	CANCER		exon	
rs17066360	NUFIP1	CANCER		exon	yes
rs3750912	NUMA1	CANCER		exon	
rs2673084	NUP50	ENCODE	chr22	exon	yes
rs2459216	OAT	CANCER		exon	yes
rs11244705	OAT	CANCER		exon	
rs1049500	ODC1	CANCER		exon	
rs3218997	OGG1	CANCER		exon	
rs3218999	OGG1	CANCER		exon	
rs3219012	OGG1	CANCER		exon	
rs762178	OLIG2	ENCODE	encode+chr21	exon	
rs3360	OPCML	CANCER		exon	
rs11034596	OR51A7	ENCODE	encode	exon	
rs10742622	OR51B2	ENCODE	encode	exon	yes
rs10837771	OR51B4	ENCODE	encode	exon	
rs4910551	OR51B5	ENCODE	encode	exon	
rs4910756	OR51B6	ENCODE	encode	exon	
rs1030726	OR51F1	ENCODE	encode	exon	
rs7114668	OR51F2	ENCODE	encode	exon	
rs12796015	OR51G1	ENCODE	encode	exon	
rs16907312	OR51G2	ENCODE	encode	exon	
rs1498486	OR51I1	ENCODE	encode	exon	

rs	Gene	Panel	Origin	Intron/Exon	Expressed
rs2030094	OR51I2	ENCODE	encode	exon	
rs2445290	OR51L1	ENCODE	encode	exon	
rs1498467	OR51M1	ENCODE	encode	exon	
rs2736588	OR51Q1	ENCODE	encode	exon	
rs12361955	OR51S1	ENCODE	encode	exon	
rs11036212	OR51V1	ENCODE	encode	exon	
rs1077126	OR52B6	ENCODE	encode	exon	
rs417425	OR52D1	ENCODE	encode	exon	
rs2445332	OR52E2	ENCODE	encode	exon	
rs7934354	OR52H1	ENCODE	encode	exon	yes
rs7936512	OR52N4	ENCODE	encode	exon	
rs2053116	OR52R1	ENCODE	encode	exon	
rs7397032	OR56B1	ENCODE	encode	exon	yes
rs3804085	OSBP2	ENCODE	chr22	exon	
rs6921341	OSTM1	ENCODE	encode	exon	
rs9486785	OSTM1	ENCODE	encode	intron	
rs208294	P2RX7	CANCER		exon	
rs2230912	P2RX7	CANCER		exon	
rs3751142	P2RX7	CANCER		exon	
rs2541953	P2RXL1	ENCODE	chr22	exon	
rs2277838	P2RXL1	ENCODE	chr22	exon	
rs1548411	P2RXL1	ENCODE	chr22	exon	
rs9625334	P2RXL1	ENCODE	chr22	intron	
rs2899365	PACSIN2	ENCODE	chr22	exon	
rs4822212	PACSIN2	ENCODE	chr22	intron	
rs11681	PALM2	CANCER		exon	yes
rs1801474	PARK2	ENCODE	disease	exon	
rs1801582	PARK2	ENCODE	disease	exon	
rs3734464	PARK2	ENCODE	disease	exon	
rs1983609	PARVB	ENCODE	chr22	exon	yes
rs1007863	PARVB	ENCODE	chr22	exon	
rs2267591	PARVB	ENCODE	chr22	intron	
rs11089032	PCBP3	ENCODE	chr21	exon	
rs4819157	PCBP3	ENCODE	chr21	intron	yes
rs14359	PCDH1	CANCER		exon	
rs11004439	PCDH15	ENCODE	encode	exon	
rs7921598	PCDH15	ENCODE	encode	exon	
rs2135720	PCDH15	ENCODE	encode	exon	
rs10825114	PCDH15	ENCODE	encode	exon	
rs4481935	PCDH15	ENCODE	encode	intron	
rs1042531	PCK1	ENCODE	disease	exon	yes
rs1804160	PCK1	ENCODE	disease	exon	
rs1062600	PCK1	ENCODE	disease	exon	
rs6070157	PCK1	ENCODE	disease	exon	
rs2236744	PCK1	ENCODE	disease	intron	
rs8632	PCNA	CANCER		exon	
rs2073380	PCNT	ENCODE	chr21	exon	
rs2073376	PCNT	ENCODE	chr21	exon	yes
rs2070426	PCNT	ENCODE	chr21	exon	
rs2839256	PCNT	ENCODE	chr21	exon	
rs7277175	PCNT	ENCODE	chr21	exon	

rs	Gene	Panel	Origin	Intron/Exon	Expressed
rs2839245	PCNT	ENCODE	chr21	exon	yes
rs16979162	PCNT	ENCODE	chr21	exon	
rs6518291	PCNT	ENCODE	chr21	exon	yes
rs2839227	PCNT	ENCODE	chr21	exon	
rs2249057	PCNT	ENCODE	chr21	exon	yes
rs16979065	PCNT	ENCODE	chr21	intron	
rs2066954	PDCD2	CANCER		exon	yes
rs8770	PDCD2	CANCER		exon	yes
rs1249950	PDE1B	CANCER		exon	
rs702530	PDE4D	ENCODE	disease	exon	yes
rs9611112	PDGFB	ENCODE	chr22	exon	
rs2285099	PDGFB	ENCODE	chr22	intron	
rs10015469	PDGFRA	CANCER		exon	
rs3690	PDGFRA	CANCER		exon	
rs2228230	PDGFRA	CANCER		exon	
rs246388	PDGFRB	CANCER		exon	
rs17110944	PDGFRB	CANCER		exon	
rs400037	PDIA2	ENCODE	encode	exon	
rs2685127	PDIA2	ENCODE	encode	exon	
rs3087657	PDIA3	ENCODE	encode	exon	yes
rs1053492	PDIA3	ENCODE	encode	exon	yes
rs10163054	PDIA3	ENCODE	encode	intron	yes
rs162881	PDLIM4	ENCODE	encode	exon	
rs162904	PDLIM4	ENCODE	encode	intron	
rs2812	PECAM1	CANCER		exon	yes
rs6809	PECAM1	CANCER		exon	yes
rs2278680	PECAM1	CANCER		exon	
rs13073	PEG10	CANCER		exon	yes
rs3750105	PEG10	CANCER		exon	yes
rs7810469	PEG10	CANCER		exon	yes
rs1055359	PEG3	CANCER		exon	
rs2191432	PEG3	CANCER		exon	
rs464385	PEX26	ENCODE	chr22	exon	yes
rs12484657	PEX26	ENCODE	chr22	exon	
rs362187	PEX26	ENCODE	chr22	intron	
rs1057034	PFKL	ENCODE	chr21	exon	yes
rs11771034	PFTK1	ENCODE	encode	exon	yes
rs11767691	PFTK1	ENCODE	encode	exon	
rs994511	PFTK1	ENCODE	encode	intron	yes
rs1065201	PGEA1	ENCODE	chr22	exon	yes
rs5757213	PGEA1	ENCODE	chr22	intron	yes
rs8185	PGF	CANCER		exon	
rs1042838	PGR	CANCER		exon	
rs1042839	PGR	CANCER		exon	
rs518162	PGR	CANCER		exon	
rs1049620	PHB	CANCER		exon	yes
rs762979	PHF21B	ENCODE	chr22	exon	
rs3747225	PHF21B	ENCODE	chr22	intron	
rs13390	PHLDA2	CANCER		exon	
rs17333103	PI3	CANCER		exon	
rs3729680	PIK3CA	CANCER		exon	yes

rs	Gene	Panel	Origin	Intron/Exon	Expressed
rs3730089	PIK3R1	CANCER		exon	yes
rs706713	PIK3R1	CANCER		exon	yes
rs3730090	PIK3R1	CANCER		exon	
rs1056847	PIK4CB	ENCODE	encode	exon	yes
rs2864115	PIK4CB	ENCODE	encode	exon	
rs12737576	PIK4CB	ENCODE	encode	intron	
rs10507	PIM1	CANCER		exon	
rs1044085	PISD	ENCODE	encode+chr22	exon	yes
rs16989334	PISD	ENCODE	encode+chr22	intron	
rs1475853	PIWIL3	ENCODE	chr22	exon	
rs131476	PIWIL3	ENCODE	chr22	intron	
rs2728121	PKD2	CANCER		exon	yes
rs10965	PKD2	CANCER		exon	
rs8135363	PKDREJ	ENCODE	chr22	exon	
rs378528	PKNOX1	ENCODE	chr21	exon	yes
rs413621	PKNOX1	ENCODE	chr21	intron	yes
rs11573156	PLA2G2A	CANCER		exon	
rs11677	PLA2G2A	CANCER		exon	
rs2236771	PLA2G2A	CANCER		exon	
rs2074735	PLA2G3	ENCODE	chr22	exon	
rs2072193	PLA2G3	ENCODE	chr22	exon	
rs2232183	PLA2G3	ENCODE	chr22	exon	
rs2232184	PLA2G3	ENCODE	chr22	intron	
rs2267369	PLA2G6	ENCODE	chr22	exon	
rs2076114	PLA2G6	ENCODE	chr22	intron	
rs8130833	PLAC4	ENCODE	chr21	exon	
rs9015	PLAC4	ENCODE	chr21	exon	
rs7278659	PLAC4	ENCODE	chr21	intron	
rs2076684	PLAGL1	CANCER		exon	yes
rs9373409	PLAGL1	CANCER		exon	yes
rs7773115	PLAGL1	CANCER		exon	
rs6061216	PLAGL2	CANCER		exon	yes
rs1804184	PLAT	CANCER		exon	
rs8178782	PLAT	CANCER		exon	
rs2227564	PLAU	CANCER		exon	
rs4065	PLAU	CANCER		exon	
rs2302524	PLAUR	CANCER		exon	yes
rs4760	PLAUR	CANCER		exon	
rs399145	PLAUR	CANCER		exon	
rs4252125	PLG	CANCER		exon	
rs871774	PLS3	CANCER		exon	
rs2075760	PLSCR3	CANCER		exon	yes
rs12602945	PLXDC1	CANCER		exon	
rs3817405	PLXDC2	CANCER		exon	yes
rs989767	PLXDC2	CANCER		exon	
rs5945431	PLXNA3	ENCODE	encode	exon	yes
rs5987266	PLXNA3	ENCODE	encode	exon	yes
rs5986981	PLXNA3	ENCODE	encode	intron	
rs9479	PML	CANCER		exon	yes
rs5742915	PML	CANCER		exon	
rs743581	PML	CANCER		exon	

rs	Gene	Panel	Origin	Intron/Exon	Expressed
rs13422	PMP22	CANCER		exon	
rs2066459	PMS1	CANCER		exon	yes
rs1805321	PMS2	CANCER		exon	
rs1805322	PMS2	CANCER		exon	
rs1805323	PMS2	CANCER		exon	
rs739231	PNPLA5	ENCODE	chr22	exon	
rs470093	PNPLA5	ENCODE	chr22	exon	
rs5764372	PNPLA5	ENCODE	chr22	intron	
rs2297285	POFUT2	ENCODE	chr21	exon	yes
rs2838859	POFUT2	ENCODE	chr21	exon	yes
rs2256000	POFUT2	ENCODE	chr21	intron	
rs2285137	POLDIP3	ENCODE	chr22	exon	
rs137091	POLDIP3	ENCODE	chr22	intron	
rs17367849	POLR3H	ENCODE	chr22	exon	
rs1810460	POLR3H	ENCODE	chr22	intron	
rs216590	POLR3K	ENCODE	encode	intron	yes
rs1800206	PPARA	ENCODE	chr22	exon	
rs4253800	PPARA	ENCODE	chr22	exon	
rs4253788	PPARA	ENCODE	chr22	intron	
rs1053046	PPARD	CANCER		exon	yes
rs2076167	PPARD	CANCER		exon	
rs3856806	PPARG	CANCER		exon	
rs1801282	PPARG	CANCER		exon	
rs3856806	PPARG	ENCODE	disease	exon	yes
rs1801282	PPARG	ENCODE	disease	exon	
rs2972164	PPARG	ENCODE	disease	intron	
rs1042040	PPAT	CANCER		exon	yes
rs3733326	PPAT	CANCER		exon	yes
rs17086746	PPAT	CANCER		exon	
rs861818	PPIL2	ENCODE	chr22	exon	
rs1103229	PPIL2	ENCODE	chr22	intron	yes
rs9610645	PPM1F	ENCODE	chr22	exon	
rs5756076	PPM1F	ENCODE	chr22	exon	
rs9607401	PPM1F	ENCODE	chr22	intron	
rs10413435	PPP2R1A	CANCER		exon	yes
rs647080	PPP2R1B	CANCER		exon	
rs2480452	PPP2R4	ENCODE	encode	exon	yes
rs7849160	PPP2R4	ENCODE	encode	intron	
rs8405	PRAME	ENCODE	chr22	exon	
rs2073725	PRAME	ENCODE	chr22	intron	
rs11264542	PRCC	CANCER		exon	yes
rs1203651	PRDM2	CANCER		exon	yes
rs3795753	PRDM2	CANCER		exon	yes
rs2903	PRDM2	CANCER		exon	
rs6958	PRKAR1A	CANCER		exon	yes
rs8905	PRKAR1A	CANCER		exon	yes
rs8080306	PRKAR1A	CANCER		exon	
rs1051992	PRKCDBP	CANCER		exon	yes
rs2242244	PRKCG	ENCODE	encode	exon	
rs3745406	PRKCG	ENCODE	encode	exon	
rs16983466	PRODH	ENCODE	chr22	exon	

rs	Gene	Panel	Origin	Intron/Exon	Expressed
rs450046	PRODH	ENCODE	chr22	exon	
rs385440	PRODH	ENCODE	chr22	intron	
rs3329	PRR5	ENCODE	chr22	exon	
rs5765890	PRR5	ENCODE	chr22	intron	
rs6667	PRSS1	CANCER		exon	
rs2824804	PRSS7	ENCODE	chr21	exon	
rs2824790	PRSS7	ENCODE	chr21	exon	
rs2824721	PRSS7	ENCODE	chr21	exon	
rs7282779	PRSS7	ENCODE	chr21	intron	
rs3736001	PSCA	CANCER		exon	
rs2294008	PSCA	CANCER		exon	
rs2239822	PSCD4	ENCODE	chr22	exon	yes
rs5995410	PSCD4	ENCODE	chr22	intron	
rs13248	PSIP1	CANCER		exon	
rs7172	PSMB4	ENCODE	encode	exon	yes
rs2066836	PTCH	CANCER		exon	
rs357564	PTCH	CANCER		exon	
rs16909910	PTCH	CANCER		exon	
rs2295997	PTCH2	CANCER		exon	yes
rs2295996	PTCH2	CANCER		exon	
rs17197	PTGER2	ENCODE	disease	exon	yes
rs1254594	PTGER2	ENCODE	disease	intron	
rs10306187	PTGS1	CANCER		exon	
rs10306190	PTGS1	CANCER		exon	
rs10306188	PTGS1	CANCER		exon	
rs2206593	PTGS2	CANCER		exon	yes
rs5275	PTGS2	CANCER		exon	yes
rs689470	PTGS2	CANCER		exon	
rs6244	PTHLH	CANCER		exon	
rs6253	PTHLH	CANCER		exon	
rs7460	PTK2	CANCER		exon	yes
rs1030526	PTK2B	CANCER		exon	yes
rs2271920	PTK2B	CANCER		exon	
rs6011873	PTK6	CANCER		exon	
rs1051709	PTK7	CANCER		exon	
rs6905948	PTK7	CANCER		exon	
rs2301262	PTPN6	CANCER		exon	
rs16997057	PTPNS1	CANCER		exon	yes
rs1065772	PTPRF	CANCER		exon	
rs10890266	PTPRF	CANCER		exon	
rs1065771	PTPRF	CANCER		exon	
rs2292245	PTPRG	CANCER		exon	
rs17634074	PTPRG	CANCER		exon	
rs2288516	PTPRH	CANCER		exon	
rs2288521	PTPRH	CANCER		exon	
rs2288419	PTPRH	CANCER		exon	
rs1558787	PTPRO	CANCER		exon	
rs6488782	PTPRO	CANCER		exon	
rs11312	PTTG1IP	ENCODE	chr21	exon	yes
rs1344110	PTTG1IP	ENCODE	chr21	intron	
rs2020945	PWP2H	ENCODE	chr21	exon	yes

rs	Gene	Panel	Origin	Intron/Exon	Expressed
rs2516521	PWP2H	ENCODE	chr21	intron	
rs4767884	PXN	CANCER		exon	yes
rs3742039	PXN	CANCER		exon	
rs737819	RAB36	ENCODE	chr22	exon	
rs5759611	RAB36	ENCODE	chr22	exon	
rs2239774	RAC2	ENCODE	chr22	exon	yes
rs12166968	RAC2	ENCODE	chr22	exon	yes
rs8137698	RAC2	ENCODE	chr22	intron	
rs8240	RAD23A	CANCER		exon	yes
rs12593359	RAD51	CANCER		exon	yes
rs11855560	RAD51	CANCER		exon	
rs1051672	RAD52	CANCER		exon	yes
rs7301931	RAD52	CANCER		exon	yes
rs7310449	RAD52	CANCER		exon	yes
rs1048771	RAD54L	CANCER		exon	yes
rs17102080	RAD54L	CANCER		exon	
rs1051208	RAF1	CANCER		exon	yes
rs3730297	RAF1	CANCER		exon	
rs4151025	RAG1	ENCODE	disease	exon	
rs872053	RAG1	ENCODE	disease	exon	
rs4151002	RAG1	ENCODE	disease	intron	
rs1065518	RALB	CANCER		exon	yes
rs934722	RALB	CANCER		exon	
rs14035	RAN	CANCER		exon	yes
rs71948	RANGAP1	ENCODE	chr22	exon	
rs1953	RANGAP1	ENCODE	chr22	exon	yes
rs1983631	RANGAP1	ENCODE	chr22	intron	
rs6573	RAP1A	CANCER		exon	yes
rs12873919	RAP2A	CANCER		exon	yes
rs2389910	RAP2A	CANCER		exon	yes
rs2229773	RARA	CANCER		exon	
rs3741434	RARG	CANCER		exon	
rs10276	RARRES1	CANCER		exon	
rs4680457	RARRES1	CANCER		exon	
rs2307064	RARRES1	CANCER		exon	
rs736212	RASD2	ENCODE	chr22	exon	
rs2272861	RASD2	ENCODE	chr22	intron	
rs11855231	RASGRF1	CANCER		exon	
rs2230518	RASGRF1	CANCER		exon	
rs6495360	RASGRF1	CANCER		exon	
rs2301562	RASGRP2	ENCODE	encode	exon	
rs2073498	RASSF1	CANCER		exon	yes
rs2073497	RASSF1	CANCER		exon	
rs3810619	RAXLX	ENCODE	chr22	exon	
rs7291048	RAXLX	ENCODE	chr22	exon	
rs361863	RAXLX	ENCODE	chr22	exon	
rs362112	RAXLX	ENCODE	chr22	intron	
rs11240353	RBBP5	CANCER		exon	yes
rs12033568	RBBP5	CANCER		exon	
rs11240354	RBBP5	CANCER		exon	
rs7593	RBBP6	CANCER		exon	yes

rs	Gene	Panel	Origin	Intron/Exon	Expressed
rs9922288	RBBP6	CANCER		exon	
rs16973840	RBBP6	CANCER		exon	
rs10748	RBL2	CANCER		exon	yes
rs3929	RBL2	CANCER		exon	yes
rs1131220	RBL2	CANCER		exon	
rs2822445	RBM11	ENCODE	chr21	exon	yes
rs382583	RBM11	ENCODE	chr21	intron	
rs6060536	RBM12	ENCODE	encode	exon	yes
rs17092937	RBM12	ENCODE	encode	intron	
rs1061474	RBM5	CANCER		exon	yes
rs522162	RDBP	CANCER		exon	yes
rs760070	RDBP	CANCER		exon	
rs1061627	RECQL	CANCER		exon	yes
rs1065751	RECQL	CANCER		exon	
rs6499	RECQL	CANCER		exon	
rs7116571	RELA	CANCER		exon	
rs5705	REN	ENCODE	disease	exon	
rs3795575	REN	ENCODE	disease	intron	
rs1800860	RET	CANCER		exon	
rs1800861	RET	CANCER		exon	
rs2075912	RET	CANCER		exon	
rs2075913	RET	CANCER		exon	
rs2075914	RET	ENCODE	disease	exon	
rs1800860	RET	ENCODE	disease	exon	
rs2075912	RET	ENCODE	disease	exon	
rs2506011	RET	ENCODE	disease	intron	
rs1047148	RFC4	CANCER		exon	yes
rs13053624	RFPL1	ENCODE	chr22	exon	
rs465736	RFPL1	ENCODE	chr22	exon	
rs2106107	RFPL1	ENCODE	chr22	intron	
rs16989988	RFPL2	ENCODE	encode+chr22	exon	
rs136473	RFPL2	ENCODE	encode+chr22	intron	
rs5749409	RFPL3	ENCODE	encode+chr22	exon	
rs16987625	RFPL3	ENCODE	encode+chr22	exon	
rs5754008	RFPL3	ENCODE	encode+chr22	intron	
rs10778502	RFX4	ENCODE	disease	exon	
rs3809240	RFX4	ENCODE	disease	intron	
rs2233851	RFX5	ENCODE	encode	exon	
rs7552906	RFX5	ENCODE	encode	exon	yes
rs2233850	RFX5	ENCODE	encode	intron	
rs1007298	Rgr	ENCODE	chr22	exon	
rs2070446	Rgr	ENCODE	chr22	exon	
rs5751713	Rgr	ENCODE	chr22	exon	
rs2070448	Rgr	ENCODE	chr22	intron	
rs3743879	RGS11	ENCODE	encode	exon	
rs2685125	RGS11	ENCODE	encode	intron	
rs3448	RHOA	CANCER		exon	yes
rs15932	RHOA	CANCER		exon	
rs11538960	RHOC	CANCER		exon	
rs2245466	RHOH	CANCER		exon	yes
rs2072770	RIBC2	ENCODE	chr22	exon	

rs	Gene	Panel	Origin	Intron/Exon	Expressed
rs1022477	RIBC2	ENCODE	chr22	exon	yes
rs9614466	RIBC2	ENCODE	chr22	intron	
rs2272990	RIPK1	CANCER		exon	
rs3212254	RIPK3	CANCER		exon	yes
rs2838113	RIPK4	CANCER		exon	
rs3746890	RIPK4	CANCER		exon	
rs3746893	RIPK4	CANCER		exon	
rs6586238	RIPK4	ENCODE	chr21	exon	
rs13049286	RIPK4	ENCODE	chr21	exon	
rs3746890	RIPK4	ENCODE	chr21	exon	
rs3746895	RIPK4	ENCODE	chr21	intron	
rs10889205	RLF	CANCER		exon	yes
rs16827078	RLF	CANCER		exon	
rs16827079	RLF	CANCER		exon	
rs3812471	RNF139	CANCER		exon	yes
rs8595	RNF185	ENCODE	chr22	exon	yes
rs5749222	RNF185	ENCODE	chr22	intron	yes
rs6668545	ROR1	CANCER		exon	
rs7527017	ROR1	CANCER		exon	
rs2230577	ROR2	CANCER		exon	
rs1135169	ROR2	CANCER		exon	
rs529038	ROS1	CANCER		exon	
rs9489124	ROS1	CANCER		exon	
rs9489143	ROS1	CANCER		exon	
rs12727	RPA1	CANCER		exon	yes
rs4822360	RTDR1	ENCODE	chr22	exon	
rs9612225	RTDR1	ENCODE	chr22	intron	
rs1131274	RYK	CANCER		exon	yes
rs1047325	S100A2	CANCER		exon	yes
rs8401	S100A2	CANCER		exon	
rs9722	S100B	ENCODE	chr21	exon	
rs881827	S100B	ENCODE	chr21	intron	
rs2294920	SAMM50	ENCODE	chr22	exon	
rs738491	SAMM50	ENCODE	chr22	intron	
rs7281104	SAMSN1	ENCODE	chr21	exon	yes
rs9617007	SAPS2	ENCODE	chr22	exon	
rs8142229	SAPS2	ENCODE	chr22	intron	yes
rs2232950	SCGB1D2	ENCODE	disease	exon	
rs2232949	SCGB1D2	ENCODE	disease	intron	
rs12148	SCO2	ENCODE	chr22	exon	
rs6003114	SCUBE1	ENCODE	chr22	exon	
rs138993	SCUBE1	ENCODE	chr22	exon	
rs11703432	SCUBE1	ENCODE	chr22	intron	
rs1947	SDF2L1	ENCODE	chr22	exon	
rs13054355	SDF2L1	ENCODE	chr22	intron	
rs17145159	SDHD	CANCER		exon	
rs1061660	SEC14L2	ENCODE	chr22	exon	
rs757660	SEC14L2	ENCODE	chr22	exon	
rs4820853	SEC14L3	ENCODE	chr22	exon	
rs2269961	SEC14L3	ENCODE	chr22	exon	
rs2240345	SEC14L3	ENCODE	chr22	exon	

rs	Gene	Panel	Origin	Intron/Exon	Expressed
rs5749102	SEC14L3	ENCODE	chr22	exon	
rs5749103	SEC14L3	ENCODE	chr22	intron	
rs9608956	SEC14L4	ENCODE	chr22	exon	
rs17738527	SEC14L4	ENCODE	chr22	exon	
rs13750	SEC14L4	ENCODE	chr22	exon	
rs9606736	SEC14L4	ENCODE	chr22	intron	
rs503068	SEC63	ENCODE	encode	exon	yes
rs642954	SEC63	ENCODE	encode	intron	yes
rs797821	SEMA3A	CANCER		exon	
rs1058425	SEMA3C	CANCER		exon	
rs2272351	SEMA3C	CANCER		exon	
rs133302	SEPTIN3	ENCODE	chr22	exon	
rs739293	SEPTIN4	ENCODE	chr22	intron	
rs137055	SERHL2	ENCODE	chr22	exon	
rs8135177	SERHL2	ENCODE	chr22	intron	
rs6108	SERPINA5	CANCER		exon	
rs6115	SERPINA5	CANCER		exon	
rs938	SERPINA5	CANCER		exon	
rs963075	SERPINB10	ENCODE	encode	exon	yes
rs17072146	SERPINB10	ENCODE	encode	exon	
rs724558	SERPINB10	ENCODE	encode	exon	yes
rs8097425	SERPINB10	ENCODE	encode	exon	yes
rs17072102	SERPINB10	ENCODE	encode	intron	yes
rs1020694	SERPINB13	ENCODE	encode	exon	
rs17071357	SERPINB13	ENCODE	encode	exon	
rs715350	SERPINB13	ENCODE	encode	intron	
rs6104	SERPINB2	CANCER		exon	yes
rs6098	SERPINB2	CANCER		exon	
rs2288288	SERPINB2	CANCER		exon	
rs6102	SERPINB2	ENCODE	encode	exon	yes
rs6098	SERPINB2	ENCODE	encode	exon	yes
rs2288288	SERPINB2	ENCODE	encode	exon	
rs2288287	SERPINB2	ENCODE	encode	intron	yes
rs12454742	SERPINB5	CANCER		exon	
rs1455555	SERPINB5	CANCER		exon	
rs894	SERPINB5	CANCER		exon	
rs17782413	SERPINB7	ENCODE	encode	exon	
rs17781440	SERPINB7	ENCODE	encode	exon	
rs1548320	SERPINB7	ENCODE	encode	intron	
rs3169983	SERPINB8	ENCODE	encode	exon	yes
rs4675	SERPIND1	ENCODE	chr22	exon	
rs5907	SERPIND1	ENCODE	chr22	exon	
rs178029	SERPIND1	ENCODE	chr22	intron	
rs1050813	SERPINE1	CANCER		exon	
rs11178	SERPINE1	CANCER		exon	
rs6092	SERPINE1	CANCER		exon	
rs268687	SERTAD1	ENCODE	disease	exon	yes
rs1210894	SEZ6L	CANCER		exon	
rs663048	SEZ6L	CANCER		exon	
rs137203	SEZ6L	CANCER		exon	
rs1210894	SEZ6L	ENCODE	chr22	exon	

rs	Gene	Panel	Origin	Intron/Exon	Expressed
rs663048	SEZ6L	ENCODE	chr22	exon	
rs137203	SEZ6L	ENCODE	chr22	exon	
rs134757	SEZ6L	ENCODE	chr22	intron	
rs523200	SF1	ENCODE	encode	exon	yes
rs680273	SF1	ENCODE	encode	intron	
rs4820004	SF3A1	ENCODE	chr22	exon	
rs10376	SF3A1	ENCODE	chr22	exon	yes
rs5749063	SF3A1	ENCODE	chr22	intron	
rs2006771	SFI1	ENCODE	encode+chr22	exon	yes
rs5753700	SFI1	ENCODE	encode+chr22	exon	yes
rs16989753	SFI1	ENCODE	encode+chr22	exon	
rs1127379	SFRP1	CANCER		exon	
rs3242	SFRP1	CANCER		exon	
rs12914	SFRP1	CANCER		exon	
rs3024822	SFTPB	CANCER		exon	
rs9752	SFTPB	CANCER		exon	
rs7316	SFTPB	CANCER		exon	
rs1126931	SFTPC	CANCER		exon	
rs7592	SFTPC	CANCER		exon	
rs1124	SFTPC	CANCER		exon	
rs1051246	SFTPD	CANCER		exon	
rs721917	SFTPD	CANCER		exon	
rs1522	SH3BGR	ENCODE	chr21	exon	yes
rs9974333	SH3BGR	ENCODE	chr21	exon	
rs6517549	SH3BGR	ENCODE	chr21	exon	
rs2837037	SH3BGR	ENCODE	chr21	intron	
rs1894529	SH3BP1	ENCODE	chr22	exon	
rs8142714	SH3BP1	ENCODE	chr22	intron	
rs1263416	SH3BP2	CANCER		exon	
rs231394	SH3BP2	CANCER		exon	
rs231399	SH3BP2	CANCER		exon	
rs1138374	SHB	CANCER		exon	
rs8190	SIAH1	CANCER		exon	yes
rs2073417	SIM2	ENCODE	chr21	exon	
rs2073601	SIM2	ENCODE	chr21	exon	
rs2269188	SIM2	ENCODE	chr21	intron	
rs2258772	SIN3B	CANCER		exon	yes
rs3184577	SIN3B	CANCER		exon	yes
rs12461731	SIN3B	CANCER		exon	
rs3772172	SKIL	CANCER		exon	yes
rs314378	SLC12A9	CANCER		exon	
rs1058396	SLC14A1	CANCER		exon	
rs2298720	SLC14A1	CANCER		exon	
rs3819179	SLC14A1	CANCER		exon	
rs2235573	SLC16A8	ENCODE	chr22	exon	
rs1004764	SLC16A8	ENCODE	chr22	intron	
rs1051266	SLC19A1	ENCODE	chr21	exon	yes
rs1051298	SLC19A1	ENCODE	chr21	exon	yes
rs3788190	SLC19A1	ENCODE	chr21	intron	
rs1048047	SLC22A18	CANCER		exon	
rs3127593	SLC22A2	CANCER		exon	

rs	Gene	Panel	Origin	Intron/Exon	Expressed
rs3127594	SLC22A2	CANCER		exon	
rs315993	SLC22A2	CANCER		exon	
rs2076828	SLC22A3	CANCER		exon	
rs2292334	SLC22A3	CANCER		exon	
rs3088442	SLC22A3	CANCER		exon	
rs10479002	SLC22A4	ENCODE	encode	exon	
rs272879	SLC22A4	ENCODE	encode	exon	
rs272893	SLC22A4	ENCODE	encode	exon	
rs419291	SLC22A4	ENCODE	encode	intron	yes
rs11568525	SLC22A5	ENCODE	encode	exon	
rs671473	SLC22A5	ENCODE	encode	intron	yes
rs11806	SLC25A17	ENCODE	chr22	exon	yes
rs11173459	SLC2A13	ENCODE	encode	exon	yes
rs4238071	SLC2A13	ENCODE	encode	intron	
rs17178345	SLC37A1	ENCODE	chr21	exon	yes
rs2839550	SLC37A1	ENCODE	chr21	exon	yes
rs8129819	SLC37A1	ENCODE	chr21	intron	
rs2429467	SLC38A4	CANCER		exon	
rs2430921	SLC38A4	CANCER		exon	
rs11183605	SLC38A4	CANCER		exon	
rs684428	SLC4A3	ENCODE	encode	exon	
rs635311	SLC4A3	ENCODE	encode	exon	
rs612078	SLC4A3	ENCODE	encode	intron	
rs17683430	SLC5A1	ENCODE	encode+chr22	exon	
rs4821027	SLC5A1	ENCODE	encode+chr22	intron	
rs5998322	SLC5A4	ENCODE	encode+chr22	exon	
rs8142488	SLC5A4	ENCODE	encode+chr22	intron	
rs1709189	SLC5A8	CANCER		exon	
rs2279834	SLC5A8	CANCER		exon	
rs6354	SLC6A4	ENCODE	disease	exon	
rs6355	SLC6A4	ENCODE	disease	exon	
rs1042173	SLC6A4	ENCODE	disease	exon	yes
rs17091302	SLC6A8	CANCER		exon	
rs2072549	SLC7A4	ENCODE	chr22	exon	
rs2270384	SLC7A4	ENCODE	chr22	exon	
rs2075278	SLC7A4	ENCODE	chr22	exon	
rs7420	SLC9A3R1	ENCODE	disease	exon	yes
rs2305216	SLC9A3R1	ENCODE	disease	exon	
rs12939882	SLC9A3R1	ENCODE	disease	intron	
rs10470	SMAD4	CANCER		exon	
rs1801250	SMAD4	CANCER		exon	
rs3809922	SMAD7	CANCER		exon	
rs3809923	SMAD7	CANCER		exon	
rs2290725	SMARCA3	CANCER		exon	yes
rs3182285	SMARCA3	CANCER		exon	yes
rs7935	SMARCA4	CANCER		exon	yes
rs11090285	SMARCB1	CANCER		exon	yes
rs5030612	SMARCB1	CANCER		exon	yes
rs5030612	SMARCB1	ENCODE	chr22	exon	yes
rs2267030	SMARCB1	ENCODE	chr22	intron	
rs6007010	SMC1L2	ENCODE	chr22	exon	yes

rs	Gene	Panel	Origin	Intron/Exon	Expressed
rs9614653	SMC1L2	ENCODE	chr22	exon	
rs9614647	SMC1L2	ENCODE	chr22	intron	
rs1061280	SMO	CANCER		exon	yes
rs1061285	SMO	CANCER		exon	yes
rs3824	SMO	CANCER		exon	yes
rs2228617	SMO	CANCER		exon	
rs2016607	SMO	CANCER		exon	
rs2075780	SMO	CANCER		exon	
rs5997872	SMTN	ENCODE	chr22	exon	yes
rs2286214	SMTN	ENCODE	chr22	intron	
rs9613204	SNAP29	ENCODE	chr22	exon	
rs165598	SNAP29	ENCODE	chr22	intron	
rs356165	SNCA	ENCODE	disease	exon	
rs1800373	SNCG	CANCER		exon	
rs9325593	SNCG	CANCER		exon	
rs1802015	SNCG	CANCER		exon	
rs17004546	SNF1LK	ENCODE	chr21	exon	
rs587087	SNF1LK	ENCODE	chr21	exon	
rs705	SNRPN	CANCER		exon	yes
rs752873	SNRPN	CANCER		exon	
rs1308137	SNX27	ENCODE	encode	exon	yes
rs17641132	SNX27	ENCODE	encode	intron	
rs139883	SOX10	ENCODE	chr22	exon	
rs139887	SOX10	ENCODE	chr22	intron	
rs1054204	SPARC	CANCER		exon	yes
rs1059279	SPARC	CANCER		exon	yes
rs1059829	SPARC	CANCER		exon	yes
rs1057233	SPI1	CANCER		exon	yes
rs4754	SPP1	CANCER		exon	
rs9138	SPP1	CANCER		exon	
rs1126616	SPP1	CANCER		exon	
rs593668	SPP2	ENCODE	encode	exon	
rs17864800	SPP2	ENCODE	encode	intron	
rs2229442	SREBF2	ENCODE	chr22	exon	
rs9607849	SREBF2	ENCODE	chr22	intron	
rs9610669	SSTR3	ENCODE	chr22	exon	
rs1053004	STAT3	CANCER		exon	yes
rs1053023	STAT3	CANCER		exon	yes
rs3744483	STAT3	CANCER		exon	yes
rs3198502	STAT5A	CANCER		exon	yes
rs17306192	STCH	ENCODE	disease+chr21	exon	
rs2822640	STCH	ENCODE	disease+chr21	intron	yes
rs194524	STEAP2	ENCODE	encode	exon	yes
rs13228098	STEAP2	ENCODE	encode	exon	
rs194520	STEAP2	ENCODE	encode	exon	yes
rs13309883	STEAP2	ENCODE	encode	intron	yes
rs673951	STK11IP	ENCODE	encode	exon	
rs7604210	STK11IP	ENCODE	encode	intron	
rs4822091	SUHW1	ENCODE	chr22	exon	
rs362230	SUHW1	ENCODE	chr22	intron	
rs414781	SUHW2	ENCODE	chr22	exon	yes

rs	Gene	Panel	Origin	Intron/Exon	Expressed
rs138056	SULT4A1	ENCODE	chr22	exon	
rs763120	SULT4A1	ENCODE	chr22	intron	
rs235292	SUMO3	ENCODE	chr21	exon	yes
rs2329902	SUMO3	ENCODE	chr21	intron	yes
rs8141797	SUSD2	ENCODE	chr22	exon	
rs3752497	SUSD2	ENCODE	chr22	exon	
rs3788368	SUSD2	ENCODE	chr22	intron	
rs1049164	SYK	CANCER		exon	yes
rs2290887	SYK	CANCER		exon	yes
rs2290890	SYK	CANCER		exon	yes
rs17772478	SYN3	ENCODE	encode+chr22	exon	
rs2097312	SYN3	ENCODE	encode+chr22	intron	
rs1010169	SYNGR1	ENCODE	chr22	exon	
rs137698	SYNGR1	ENCODE	chr22	intron	
rs2254562	SYNJ1	ENCODE	encode+chr21	exon	yes
rs13554	SYNJ1	ENCODE	encode+chr21	exon	yes
rs844985	SYNJ1	ENCODE	encode+chr21	intron	yes
rs907609	SYT8	ENCODE	encode	exon	
rs2070930	TAL1	CANCER		exon	
rs7664	TAL1	CANCER		exon	
rs2070929	TAL1	CANCER		exon	
rs4823086	TBC1D10A	ENCODE	chr22	exon	yes
rs2097919	TBC1D10A	ENCODE	chr22	intron	
rs15411	TBC1D22A	ENCODE	chr22	exon	yes
rs2301558	TBX1	ENCODE	chr22	exon	
rs8137465	TBX1	ENCODE	chr22	intron	
rs12721470	TBX21	ENCODE	disease	exon	
rs10514934	TBX21	ENCODE	disease	intron	
rs2070116	TCF20	ENCODE	chr22	exon	yes
rs9611746	TCF20	ENCODE	chr22	intron	yes
rs6567211	TCF4	CANCER		exon	yes
rs8766	TCF4	CANCER		exon	yes
rs1056877	TCF7L2	CANCER		exon	
rs10418	TCN2	ENCODE	chr22	exon	yes
rs4820889	TCN2	ENCODE	chr22	exon	
rs9621049	TCN2	ENCODE	chr22	exon	yes
rs1801198	TCN2	ENCODE	chr22	exon	yes
rs16988828	TCN2	ENCODE	chr22	intron	
rs6517105	TCP10L	ENCODE	encode+chr21	exon	yes
rs1015048	TCP10L	ENCODE	encode+chr21	intron	
rs4135113	TDG	CANCER		exon	yes
rs3189859	TDGF1	CANCER		exon	yes
rs4141433	TEF	ENCODE	chr22	exon	
rs17365991	TEF	ENCODE	chr22	exon	
rs2273719	TEK	CANCER		exon	
rs542913	TEK	CANCER		exon	
rs639225	TEK	CANCER		exon	
rs938886	TEP1	CANCER		exon	yes
rs1760903	TEP1	CANCER		exon	
rs1760904	TEP1	CANCER		exon	
rs2853690	TERT	CANCER		exon	

rs	Gene	Panel	Origin	Intron/Exon	Expressed
rs4710	TES	CANCER		exon	yes
rs2896181	TES	CANCER		exon	
rs4710	TES	ENCODE	encode	exon	yes
rs4428611	TES	ENCODE	encode	intron	yes
rs9429072	TESK2	CANCER		exon	yes
rs11169735	TFCP2	ENCODE	disease	exon	
rs1015149	TFEB	ENCODE	encode	exon	
rs2273068	TFEB	ENCODE	encode	exon	yes
rs14063	TFEB	ENCODE	encode	exon	
rs2073157	TFEB	ENCODE	encode	intron	yes
rs13276	TFF1	CANCER		exon	
rs225334	TFF2	CANCER		exon	
rs225334	TFF2	ENCODE	chr21	exon	yes
rs225335	TFF2	ENCODE	chr21	intron	
rs11701143	TFF3	ENCODE	chr21	exon	
rs2236705	TFF3	ENCODE	chr21	intron	
rs7297	TFG	CANCER		exon	yes
rs6441516	TFG	CANCER		exon	
rs1008530	TFIP11	ENCODE	chr22	exon	
rs17402286	TFIP11	ENCODE	chr22	exon	
rs2032574	TFIP11	ENCODE	chr22	intron	yes
rs4264	TFPI2	CANCER		exon	
rs4517	TFPI2	CANCER		exon	
rs17788379	TFRC	CANCER		exon	yes
rs406271	TFRC	CANCER		exon	yes
rs1058211	TGFA	CANCER		exon	
rs3732253	TGFA	CANCER		exon	
rs473698	TGFA	CANCER		exon	
rs538118	TGFA	CANCER		exon	
rs1800472	TGFB1	ENCODE	disease	exon	
rs900	TGFB2	CANCER		exon	
rs11466412	TGFB2	CANCER		exon	
rs10129478	TGFB3	CANCER		exon	
rs7854	TGFBI	CANCER		exon	
rs868	TGFBR1	CANCER		exon	yes
rs2228048	TGFBR2	CANCER		exon	
rs902	TGFBR3	CANCER		exon	yes
rs6356	TH	ENCODE	encode	exon	
rs2070762	TH	ENCODE	encode	intron	
rs2292305	THBS1	CANCER		exon	yes
rs1051442	THBS1	CANCER		exon	
rs2228263	THBS1	CANCER		exon	
rs1040	THBS2	CANCER		exon	
rs3253	THBS2	CANCER		exon	
rs8089	THBS2	CANCER		exon	
rs6141	THPO	CANCER		exon	
rs762194	TIAM1	CANCER		exon	
rs1199039	TIE1	CANCER		exon	
rs1043428	TIMP1	CANCER		exon	
rs2277698	TIMP2	CANCER		exon	
rs2267184	TIMP3	CANCER		exon	

rs	Gene	Panel	Origin	Intron/Exon	Expressed
rs2267185	TIMP3	CANCER		exon	
rs9862	TIMP3	CANCER		exon	
rs1427384	TIMP3	ENCODE	encode+chr22	exon	
rs9862	TIMP3	ENCODE	encode+chr22	exon	
rs5749512	TIMP3	ENCODE	encode+chr22	intron	
rs2291166	TJP1	CANCER		exon	
rs17062695	TJP2	CANCER		exon	yes
rs3812536	TJP2	CANCER		exon	yes
rs1065769	TK1	CANCER		exon	yes
rs2872817	TKTL1	ENCODE	encode	exon	
rs17281251	TKTL1	ENCODE	encode	intron	
rs10771314	TM7SF3	CANCER		exon	yes
rs4856	TM7SF3	CANCER		exon	yes
rs641738	TMC4	ENCODE	encode	exon	
rs6509847	TMC4	ENCODE	encode	intron	
rs2838475	TMEM1	ENCODE	chr21	exon	yes
rs3788097	TMEM1	ENCODE	chr21	intron	
rs2834216	TMEM50B	ENCODE	encode+chr21	exon	yes
rs1532	TMEM50B	ENCODE	encode+chr21	exon	yes
rs3743887	TMEM8	ENCODE	encode	exon	yes
rs2838038	TMPRSS2	ENCODE	chr21	exon	
rs928302	TMPRSS3	ENCODE	chr21	exon	
rs2839500	TMPRSS3	ENCODE	chr21	exon	yes
rs2839492	TMPRSS3	ENCODE	chr21	intron	
rs8924	TMPRSS4	CANCER		exon	
rs11704654	TMPRSS6	ENCODE	chr22	exon	
rs2235324	TMPRSS6	ENCODE	chr22	exon	
rs4820268	TMPRSS6	ENCODE	chr22	exon	yes
rs5756504	TMPRSS6	ENCODE	chr22	intron	
rs2274750	TNC	CANCER		exon	
rs3093665	TNF	CANCER		exon	yes
rs2515924	TNF	CANCER		exon	
rs4645843	TNF	CANCER		exon	
rs3093704	TNFAIP1	CANCER		exon	yes
rs2230229	TNFRSF10A	CANCER		exon	
rs1047275	TNFRSF10B	CANCER		exon	yes
rs11135695	TNFRSF10B	CANCER		exon	yes
rs1047266	TNFRSF10B	CANCER		exon	
rs7957	TNFRSF10D	CANCER		exon	yes
rs7290134	TNFRSF13C	ENCODE	chr22	exon	yes
rs1804532	TNFRSF1A	CANCER		exon	
rs4149584	TNFRSF1A	ENCODE	disease	exon	
rs1860545	TNFRSF1A	ENCODE	disease	intron	
rs1061622	TNFRSF1B	CANCER		exon	yes
rs1061624	TNFRSF1B	CANCER		exon	yes
rs1061628	TNFRSF1B	CANCER		exon	yes
rs1131532	TNFSF10	CANCER		exon	yes
rs1131535	TNFSF10	CANCER		exon	yes
rs1131542	TNFSF10	CANCER		exon	yes
rs3181368	TNFSF8	CANCER		exon	
rs2075759	TNK1	CANCER		exon	

rs	Gene	Panel	Origin	Intron/Exon	Expressed
rs3761718	TNK2	CANCER		exon	
rs12485008	TNRC6B	ENCODE	chr22	exon	yes
rs6001734	TNRC6B	ENCODE	chr22	intron	yes
rs4626	TOB1	CANCER		exon	yes
rs202641	TOB2	ENCODE	chr22	exon	yes
rs5750668	TOMM22	ENCODE	chr22	exon	yes
rs13695	TOP2A	CANCER		exon	yes
rs4968187	TP53	CANCER		exon	
rs1800372	TP53	CANCER		exon	
rs689647	TP53BP1	ENCODE	encode	exon	yes
rs690367	TP53BP1	ENCODE	encode	exon	yes
rs2602141	TP53BP1	ENCODE	encode	exon	yes
rs1058298	TP53BP1	ENCODE	encode	intron	yes
rs12048341	TP73	CANCER		exon	
rs1801174	TP73	CANCER		exon	
rs9662633	TP73	ENCODE	disease	exon	
rs2181484	TP73	ENCODE	disease	intron	yes
rs17110747	TPH2	ENCODE	disease	exon	
rs4290270	TPH2	ENCODE	disease	exon	
rs11178999	TPH2	ENCODE	disease	intron	
rs3753565	TPR	CANCER		exon	yes
rs1131877	TRAF3	CANCER		exon	yes
rs1670195	TRIM29	CANCER		exon	
rs3740996	TRIM5	ENCODE	encode	exon	yes
rs3740995	TRIM5	ENCODE	encode	exon	yes
rs10769167	TRIM5	ENCODE	encode	intron	yes
rs7120209	TRIM6	ENCODE	encode	exon	yes
rs3751005	TRIM6	ENCODE	encode	exon	
rs7927012	TRIM6	ENCODE	encode	intron	yes
rs16933844	TRIM6-TRIM34	ENCODE	encode	exon	
rs4821708	TRIOBP	ENCODE	chr22	exon	
rs5756805	TRIOBP	ENCODE	chr22	intron	
rs6706	TRIP6	CANCER		exon	yes
rs13585	TRMU	ENCODE	chr22	exon	yes
rs2074234	TRPM5	CANCER		exon	
rs800342	TRPM5	CANCER		exon	
rs17865681	TRPM8	ENCODE	encode	exon	
rs11563071	TRPM8	ENCODE	encode	exon	
rs11563208	TRPM8	ENCODE	encode	exon	
rs17862932	TRPM8	ENCODE	encode	exon	
rs13004520	TRPM8	ENCODE	encode	exon	
rs10170399	TRPM8	ENCODE	encode	intron	
rs1050700	TSC1	CANCER		exon	
rs2809243	TSC1	CANCER		exon	
rs2809244	TSC1	CANCER		exon	
rs1051771	TSC2	CANCER		exon	yes
rs7595	TSEN34	ENCODE	encode	exon	
rs635608	TSEN34	ENCODE	encode	intron	yes
rs2839536	TSGA2	ENCODE	chr21	exon	yes
rs2839531	TSGA2	ENCODE	chr21	exon	yes
rs7283245	TSGA2	ENCODE	chr21	intron	

rs	Gene	Panel	Origin	Intron/Exon	Expressed
rs3783941	TSHR	ENCODE	disease	exon	
rs1991517	TSHR	ENCODE	disease	exon	
rs2239610	TSHR	ENCODE	disease	intron	
rs2074022	TSPAN32	ENCODE	encode	exon	
rs10831733	TSPAN32	ENCODE	encode	intron	
rs1057769	TSSC4	CANCER		exon	
rs2234278	TSSC4	CANCER		exon	
rs2234279	TSSC4	CANCER		exon	
rs1052756	TSSK2	ENCODE	chr22	exon	
rs2835665	TTC3	ENCODE	chr21	exon	yes
rs1053966	TTC3	ENCODE	chr21	exon	yes
rs1053808	TTC3	ENCODE	chr21	exon	yes
rs2835574	TTC3	ENCODE	chr21	exon	yes
rs9974286	TTC3	ENCODE	chr21	intron	yes
rs2076155	TTLL1	ENCODE	chr22	exon	yes
rs5759125	TTLL1	ENCODE	chr22	exon	yes
rs2072884	TTLL1	ENCODE	chr22	intron	
rs3745420	TTYH1	ENCODE	encode	exon	
rs1077829	TTYH1	ENCODE	encode	intron	
rs12159975	TUBA8	ENCODE	chr22	exon	yes
rs1053395	TUBB4	CANCER		exon	yes
rs3099129	TUBB4	CANCER		exon	yes
rs11703226	TUBGCP6	ENCODE	chr22	exon	yes
rs4838864	TUBGCP6	ENCODE	chr22	exon	
rs2272854	TUBGCP6	ENCODE	chr22	intron	
rs1891592	TUFT1	ENCODE	encode	exon	
rs3790507	TUFT1	ENCODE	encode	intron	
rs5992495	TXNRD2	ENCODE	chr22	exon	yes
rs1044732	TXNRD2	ENCODE	chr22	exon	yes
rs280523	TYK2	CANCER		exon	
rs2790	TYMS	CANCER		exon	yes
rs699517	TYMS	CANCER		exon	yes
rs3786362	TYMS	CANCER		exon	
rs2277536	TYRO3	CANCER		exon	
rs1802029	TYROBP	ENCODE	disease	exon	
rs3817624	TYROBP	ENCODE	disease	intron	
rs6554	UBA52	CANCER		exon	yes
rs13048049	UBASH3A	ENCODE	chr21	exon	
rs11203202	UBASH3A	ENCODE	chr21	intron	
rs1060574	UBQLN3	ENCODE	encode	exon	
rs10929303	UGT1A1	CANCER		exon	
rs11683356	UGT1A10	ENCODE	encode	exon	
rs17868336	UGT1A3	ENCODE	encode	exon	
rs11891311	UGT1A3	ENCODE	encode	intron	
rs6755571	UGT1A4	ENCODE	encode	exon	
rs871514	UGT1A4	ENCODE	encode	intron	
rs3755321	UGT1A5	ENCODE	encode	exon	
rs17862870	UGT1A5	ENCODE	encode	intron	
rs7577677	UGT1A7	CANCER		exon	
rs7577677	UGT1A7	ENCODE	encode	exon	
rs4663888	UGT1A7	ENCODE	encode	intron	

rs	Gene	Panel	Origin	Intron/Exon	Expressed
rs10929303	UGT1A9	ENCODE	disease+encode	exon	
rs4663871	UGT1A9	ENCODE	disease+encode	intron	
rs7280633	UMODL1	ENCODE	chr21	exon	yes
rs3819142	UMODL1	ENCODE	chr21	exon	
rs220159	UMODL1	ENCODE	chr21	exon	yes
rs220146	UMODL1	ENCODE	chr21	exon	
rs2839464	UMODL1	ENCODE	chr21	exon	
rs2072797	UNC84B	ENCODE	chr22	exon	yes
rs1043524	UNC84B	ENCODE	chr22	exon	yes
rs138702	UNC84B	ENCODE	chr22	intron	
rs1314	UPB1	ENCODE	chr22	exon	yes
rs2070474	UPB1	ENCODE	chr22	exon	
rs3788369	UPB1	ENCODE	chr22	intron	
rs1057356	UPK3A	ENCODE	chr22	exon	
rs2075951	UPK3A	ENCODE	chr22	exon	
rs2673088	UPK3A	ENCODE	chr22	exon	
rs2142551	UPK3A	ENCODE	chr22	intron	
rs2274802	USP16	ENCODE	chr21	exon	yes
rs1034123	USP16	ENCODE	chr21	intron	yes
rs3180408	USP18	ENCODE	chr22	exon	yes
rs2297248	USP25	ENCODE	chr21	exon	
rs1027392	USP29	CANCER		exon	
rs3764574	USP29	CANCER		exon	
rs3795003	USP29	CANCER		exon	
rs2185798	USP49	ENCODE	encode	exon	yes
rs1010	VAMP8	CANCER		exon	yes
rs1058588	VAMP8	CANCER		exon	yes
rs3731828	VAMP8	CANCER		exon	yes
rs602990	VAV2	CANCER		exon	yes
rs11887	VBP1	CANCER		exon	yes
rs3599	VBP1	CANCER		exon	
rs2010963	VEGF	CANCER		exon	yes
rs25648	VEGF	CANCER		exon	yes
rs3025039	VEGF	CANCER		exon	yes
rs1642742	VHL	CANCER		exon	
rs2230143	VIL2	CANCER		exon	
rs7294	VKORC1	ENCODE	disease	exon	yes
rs1800387	VWF	ENCODE	disease	exon	
rs1063857	VWF	ENCODE	disease	exon	
rs1800380	VWF	ENCODE	disease	exon	
rs1800386	VWF	ENCODE	disease	exon	
rs216867	VWF	ENCODE	disease	exon	
rs933408	VWF	ENCODE	disease	intron	
rs2248490	WDR4	ENCODE	chr21	exon	yes
rs11911090	WDR4	ENCODE	chr21	exon	
rs6586250	WDR4	ENCODE	chr21	exon	yes
rs3746939	WDR4	ENCODE	chr21	exon	yes
rs9977828	WDR4	ENCODE	chr21	intron	yes
rs1049403	WEE1	CANCER		exon	yes
rs3741627	WNT10B	CANCER		exon	
rs2024233	WNT2	CANCER		exon	yes

rs	Gene	Panel	Origin	Intron/Exon	Expressed
rs2024233	WNT2	ENCODE	encode	exon	yes
rs733153	WNT2	ENCODE	encode	intron	
rs2273368	WNT2B	CANCER		exon	
rs3773606	WNT5A	CANCER		exon	
rs1060180	WRB	ENCODE	encode+chr21	exon	yes
rs2094874	WRB	ENCODE	encode+chr21	intron	
rs1346044	WRN	CANCER		exon	yes
rs1800392	WRN	CANCER		exon	yes
rs3087425	WRN	CANCER		exon	
rs1042347	WT1	CANCER		exon	
rs5030317	WT1	CANCER		exon	
rs5030320	WT1	CANCER		exon	
rs5748648	XKR3	ENCODE	chr22	exon	
rs5748622	XKR3	ENCODE	chr22	exon	
rs16981669	XKR3	ENCODE	chr22	intron	
rs3176751	XPA	CANCER		exon	
rs2228000	XPC	CANCER		exon	yes
rs2229090	XPC	CANCER		exon	yes
rs2470352	XPC	CANCER		exon	yes
rs1799782	XRCC1	CANCER		exon	yes
rs3547	XRCC1	CANCER		exon	yes
rs2228487	XRCC1	CANCER		exon	
rs3218536	XRCC2	CANCER		exon	yes
rs3218545	XRCC2	CANCER		exon	
rs3218539	XRCC2	CANCER		exon	
rs861539	XRCC3	CANCER		exon	
rs1056503	XRCC4	CANCER		exon	yes
rs2035990	XRCC4	CANCER		exon	yes
rs1051677	XRCC5	CANCER		exon	yes
rs1051685	XRCC5	CANCER		exon	yes
rs207906	XRCC5	CANCER		exon	yes
rs1060922	YES1	CANCER		exon	yes
rs1061035	YES1	CANCER		exon	yes
rs1049583	YWHAH	CANCER		exon	
rs2278699	ZAP70	CANCER		exon	yes
rs3192177	ZAP70	CANCER		exon	
rs910796	ZBED4	ENCODE	chr22	exon	yes
rs761878	ZBED4	ENCODE	chr22	intron	
rs5751084	ZC3H7B	ENCODE	chr22	exon	yes
rs9607793	ZC3H7B	ENCODE	chr22	exon	
rs4822021	ZC3H7B	ENCODE	chr22	exon	yes
rs2024567	ZC3H7B	ENCODE	chr22	intron	
rs9605069	ZDHHC8	ENCODE	chr22	exon	
rs175174	ZDHHC8	ENCODE	chr22	intron	
rs1860565	ZIM2	CANCER		exon	
rs2302376	ZIM2	CANCER		exon	
rs2370134	ZIM3	CANCER		exon	
rs7251328	ZIM3	CANCER		exon	
rs2070132	ZNF146	CANCER		exon	yes
rs4806293	ZNF146	CANCER		exon	yes
rs11041108	ZNF215	CANCER		exon	yes

rs	Gene	Panel	Origin	Intron/Exon	Expressed
rs2239730	ZNF215	CANCER		exon	yes
rs2239731	ZNF215	CANCER		exon	yes
rs2245431	ZNF294	ENCODE	chr21	exon	yes
rs2254796	ZNF294	ENCODE	chr21	exon	yes
rs11088101	ZNF294	ENCODE	chr21	intron	yes
rs11702690	ZNF295	ENCODE	chr21	exon	yes
rs2298265	ZNF687	ENCODE	encode	exon	yes
rs9612413	ZNF70	ENCODE	chr22	exon	yes
rs5759986	ZNF70	ENCODE	chr22	intron	
rs4020	ZNF74	ENCODE	chr22	exon	yes
rs362199	ZNF74	ENCODE	chr22	intron	
rs2241666	ZWINT	CANCER		exon	yes
