# Supplementary material for: Differential Allelic Expression in the Human Genome: A Robust Approach To Identify Genetic and Epigenetic Cis-Acting Mechanisms Regulating Gene Expression
Source: PLoS Genet. 2008 Feb 29;4(2):e1000006. doi: 10.1371/journal.pgen.1000006 (PMC2265535; doi:10.1371/journal.pgen.1000006)
Supplement: Table S2 — All SNPs expressed in at least three heterozygous individuals (0.05 MB PDF) [file pgen.1000006.s015.pdf]

Supplemental Table 2. All SNPs expressed in at least three heterozygous individuals

rs	Gene	Panel	chr	# Hets	Variance & Mean	meanAI	A vs. B
rs135106	A4GALT	Encode	22	10		NA	NA
rs17064	ABCB1	Encode	7	7		NA	NA
rs17064	ABCB1	Cancer	7	6		NA	NA
rs3842	ABCB1	Cancer	7	14	High variance	NA	NA
rs2302387	ABCB4	Cancer	7	14		NA	NA
rs562	ABCC5	Cancer	3	28		55:45	0 vs. 22
rs183436	ABCG1	Encode	21	36		75:25	0 vs. 34
rs1056209	ABL1	Cancer	9	15		NA	NA
rs7457	ABL1	Cancer	9	18		NA	NA
rs2274230	ABL2	Cancer	1	9	High variance	70:30	1 vs. 6
rs1046048	ACVR2B	Cancer	3	36		50:50	1 vs. 12
rs2838820	ADARB1	Encode	21	22		55:45	2 vs. 11
rs1342387	ADIPOR1	Encode	1	27		50:50	2 vs. 1
rs7539542	ADIPOR1	Encode	1	34		55:45	0 vs. 26
rs2525485	AFF4	Encode	5	40		50:50	3 vs. 13
rs42395	AFF4	Encode	5	40		50:50	0 vs. 13
rs739863	AFF4	Encode	5	25		55:45	25 vs. 0
rs13847	AGPAT3	Encode	21	27		50:50	1 vs. 9
rs2066853	AHR	Cancer	7	7		55:45	5 vs. 0
rs4978877	AKAP2	Cancer	9	9		50:50	2 vs. 0
rs2498799	AKT1	Cancer	14	34		55:45	0 vs. 26
rs1321	ALG12	Encode	22	32		NA	NA
rs7286796	AP1B1	Encode	22	39		50:50	2 vs. 0
rs397768	APC	Cancer	5	49		50:50	4 vs. 17
rs448475	APC	Cancer	5	55		50:50	4 vs. 31
rs866006	APC	Cancer	5	53		50:50	1 vs. 10
rs2281106	APOBEC3C	Encode	22	29		55:45	0 vs. 10
rs2076101	APOBEC3F	Encode	22	33		50:50	11 vs. 0
rs5750735	APOBEC3F	Encode	22	34		50:50	5 vs. 6
rs12160242	APOBEC3G	Encode	22	10		NA	NA
rs5757465	APOBEC3G	Encode	22	39		55:45	0 vs. 38
rs1807740	APOL1	Encode	22	15		50:50	1 vs. 4
rs2239785	APOL1	Encode	22	25		55:45	7 vs. 10
rs9610467	APOL1	Encode	22	13		NA	NA
rs9610460	APOL2	Encode	22	46		55:45	26 vs. 6
rs132618	APOL3	Encode	22	33		50:50	0 vs. 13
rs132621	APOL3	Encode	22	27		50:50	2 vs. 9
rs4393836	ARFGAP3	Encode	22	8		50:50	4 vs. 0
rs738535	ARFGAP3	Encode	22	44		50:50	3 vs. 1
rs244468	ARHGAP26	Encode	5	36		50:50	6 vs. 4
rs4703	ARHGDIB	Cancer	12	40		50:50	0 vs. 23
rs921	ARHGDIB	Cancer	12	12		NA	NA
rs1051861	ARID4A	Cancer	14	36		55:45	0 vs. 26
rs11552229	ARNT	Cancer	1	36		50:50	8 vs. 0
rs2228099	ARNT	Cancer	1	37		50:50	0 vs. 2
rs139297	ARP10	Encode	22	34		50:50	2 vs. 11
rs139316	ARP10	Encode	22	35		55:45	29 vs. 0
rs6151429	ARSA	Encode	22	24		85:15	24 vs. 0
rs743616	ARSA	Encode	22	37		60:40	18 vs. 12

rs	Gene	Panel	chr	# Hets	Variance & Mean	meanAI	A vs. B
rs36571	ASCC2	Encode	22	6		NA	NA
rs9782	ASCL1	Cancer	12	28		60:40	3 vs. 11
rs1800889	ATM	Cancer	11	4		50:50	0 vs. 3
rs609261	ATM	Cancer	11	41		55:45	1 vs. 29
rs1320525	ATP11A	Encode	13	30		50:50	11 vs. 2
rs423117	ATP11A	Encode	13	15		55:45	1 vs. 6
rs17728665	ATP5O	Encode	21	14		50:50	0 vs. 13
rs3532	ATP6V1E1	Encode	22	29		60:40	29 vs. 0
rs5992084	ATP6V1E1	Encode	22	33		55:45	0 vs. 32
rs2227928	ATR	Cancer	3	35		55:45	2 vs. 17
rs134860	ATXN10	Encode	22	16		50:50	1 vs. 1
rs9614791	ATXN10	Encode	22	6		50:50	2 vs. 1
rs1053338	ATXN7	Cancer	3	36		50:50	1 vs. 11
rs3774729	ATXN7	Cancer	3	39		50:50	1 vs. 19
rs393521	AXIN1	Encode	16	24		55:45	0 vs. 22
rs393521	AXIN1	Cancer	16	21		55:45	0 vs. 20
rs758033	AXIN1	Encode	16	23		50:50	0 vs. 12
rs1046210	BACE2	Encode	21	42		50:50	7 vs. 7
rs12149	BACE2	Encode	21	30		55:45	8 vs. 5
rs388707	BACH1	Encode	21	34		50:50	1 vs. 10
rs1043782	BAG4	Cancer	8	32		55:45	31 vs. 0
rs210135	BAK1	Cancer	6	21		60:40	0 vs. 21
rs1048108	BARD1	Cancer	2	33		55:45	0 vs. 18
rs2070096	BARD1	Cancer	2	21		50:50	2 vs. 18
rs2229571	BARD1	Cancer	2	44		50:50	5 vs. 4
rs4645900	BAX	Cancer	19	6		NA	NA
rs704243	BAX	Cancer	19	15		NA	NA
rs128941	BC002942	Encode	22	34		55:45	0 vs. 11
rs1564483	BCL2	Cancer	18	34		50:50	0 vs. 11
rs4987852	BCL2	Cancer	18	5		55:45	0 vs. 5
rs3826007	BCL2A1	Cancer	15	28		50:50	22 vs. 0
rs4488761	BCL2L13	Encode	22	43		50:50	1 vs. 8
rs180817	BCR	Cancer	22	15		60:40	6 vs. 2
rs11090143	BIK	Encode	22	7	High variance	NA	NA
rs1474593	BIRC4	Encode	X	19	High variance	90:10	11 vs. 8
rs17330644	BIRC4	Encode	X	15	High variance	80:20	10 vs. 4
rs5956583	BIRC4	Cancer	X	21	High variance	90:10	13 vs. 8
rs5958343	BIRC4	Cancer	X	26	High variance	85:15	14 vs. 11
rs8371	BIRC4	Cancer	X	20	High variance	85:15	15 vs. 5
rs9856	BIRC4	Cancer	X	24	High variance	85:15	15 vs. 8
rs1042489	BIRC5	Cancer	17	52		50:50	10 vs. 0
rs2250788	BLK	Cancer	8	16		NA	NA
rs1063147	BLM	Cancer	15	35		50:50	2 vs. 18
rs17273563	BLM	Cancer	15	35		50:50	2 vs. 10
rs17274095	BLM	Cancer	15	35		55:45	0 vs. 21
rs235768	BMP2	Cancer	20	7	High variance	60:40	3 vs. 3
rs1044104	BMP6	Cancer	6	22		60:40	6 vs. 9
rs11528010	BMPR1A	Cancer	10	33		55:45	32 vs. 0
rs7078571	BMPR1A	Cancer	10	6		NA	NA
rs1048829	BMPR2	Cancer	2	39		50:50	4 vs. 4
rs1061157	BMPR2	Cancer	2	15		50:50	4 vs. 1

rs	Gene	Panel	chr	# Hets	Variance & Mean	meanAI	A vs. B
rs3731696	BMPR2	Cancer	2	15		50:50	0 vs. 2
rs16940	BRCA1	Cancer	17	23		50:50	0 vs. 10
rs1799966	BRCA1	Cancer	17	24		50:50	2 vs. 6
rs799917	BRCA1	Cancer	17	25		50:50	0 vs. 11
rs144848	BRCA2	Cancer	13	43		50:50	0 vs. 2
rs15869	BRCA2	Cancer	13	28		50:50	4 vs. 7
rs1801406	BRCA2	Cancer	13	32		50:50	13 vs. 11
rs543304	BRCA2	Cancer	13	31		50:50	5 vs. 0
rs1041439	BRWD1	Encode	21	10		NA	NA
rs13625	BRWD1	Encode	21	36		50:50	1 vs. 2
rs2056844	BRWD1	Encode	21	40		50:50	0 vs. 24
rs2297255	BRWD1	Encode	21	13		NA	NA
rs8259	BSG	Cancer	19	34		55:45	0 vs. 23
rs1057403	BTK	Cancer	X	4		85:15	3 vs. 1
rs700	BTK	Cancer	X	22	High variance	85:15	11 vs. 10
rs6971	BZRP	Encode	22	30		55:45	7 vs. 15
rs12886549	C14orf154	Encode	14	31		55:45	0 vs. 26
rs1045001	C16orf33	Encode	16	21		50:50	1 vs. 12
rs229519	C1QTNF6	Encode	22	35		55:45	1 vs. 18
rs7290488	C1QTNF6	Encode	22	21		NA	NA
rs4911494	C20orf44	Encode	20	37		50:50	2 vs. 4
rs2837029	C21orf13	Encode	21	15	Mean shifted	70:30	0 vs. 11
rs2835246	C21orf18	Encode	21	11		NA	NA
rs8738	C21orf18	Encode	21	11		NA	NA
rs13050556	C21orf25	Encode	21	43		50:50	5 vs. 11
rs3168	C21orf25	Encode	21	17		50:50	2 vs. 0
rs3746906	C21orf25	Encode	21	45		50:50	0 vs. 22
rs2838491	C21orf33	Encode	21	21		55:45	1 vs. 8
rs11702555	C21orf5	Encode	21	32		50:50	18 vs. 11
rs3827183	C21orf5	Encode	21	14		50:50	1 vs. 3
rs9749	C21orf51	Encode	21	44		50:50	9 vs. 1
rs7283236	C21orf69	Encode	21	37		55:45	17 vs. 4
rs2832236	C21orf7	Encode	21	40	High variance	65:35	11 vs. 15
rs9978281	C21orf7	Encode	21	12	High variance	NA	NA
rs1047978	C21orf91	Encode	21	33		55:45	0 vs. 28
rs2258119	C21orf91	Encode	21	25		60:40	25 vs. 0
rs9704	C22orf13	Encode	22	22		55:45	0 vs. 21
rs5758511	C22orf18	Encode	22	34		60:40	0 vs. 33
rs6002555	C22orf18	Encode	22	27		50:50	4 vs. 4
rs1049534	C22orf19	Encode	22	26		50:50	0 vs. 4
rs737976	C22orf19	Encode	22	44		50:50	0 vs. 27
rs6007594	C22orf8	Encode	22	28		70:30	0 vs. 22
rs1043731	C22orf9	Encode	22	26		50:50	0 vs. 14
rs2545166	C5orf18	Cancer	5	52		50:50	2 vs. 35
rs3317	C5orf18	Cancer	5	55		50:50	12 vs. 12
rs311677	C6orf150	Encode	6	5		NA	NA
rs610913	C6orf150	Encode	6	30		50:50	9 vs. 0
rs9352000	C6orf150	Encode	6	15		NA	NA
rs5760180	CABIN1	Encode	22	21		50:50	1 vs. 5
rs2271627	CAPG	Cancer	2	32		55:45	16 vs. 3
rs2975767	CAPN10	Cancer	2	11		50:50	4 vs. 2

rs	Gene	Panel	chr	# Hets	Variance & Mean	meanAI	A vs. B
rs3173936	CAPZA2	Encode	7	21		NA	NA
rs3807999	CAPZA2	Encode	7	20		NA	NA
rs3135500	CARD15	Cancer	16	40		50:50	1 vs. 3
rs13006529	CASP10	Cancer	2	33		60:40	0 vs. 25
rs3900115	CASP10	Cancer	2	38		50:50	1 vs. 10
rs1049216	CASP3	Cancer	4	31		50:50	0 vs. 19
rs1042891	CASP6	Cancer	4	41		60:40	0 vs. 41
rs12443102	CATSPER2	Encode	15	8		NA	NA
rs8042868	CATSPER2	Encode	15	8		NA	NA
rs6867	CAV1	Cancer	7	11		NA	NA
rs8713	CAV1	Cancer	7	15		55:45	10 vs. 0
rs1047417	CBL	Cancer	11	31		50:50	2 vs. 4
rs1042852	CBLB	Encode	3	38		55:45	0 vs. 36
rs2305035	CBLB	Cancer	3	40		50:50	8 vs. 0
rs2305035	CBLB	Encode	3	33		50:50	0 vs. 1
rs7649466	CBLB	Encode	3	30		50:50	9 vs. 3
rs1005696	CBR1	Encode	21	40		55:45	6 vs. 13
rs20572	CBR1	Encode	21	11		NA	NA
rs706209	CBS	Encode	21	42		55:45	2 vs. 27
rs2235140	CBX6	Encode	22	31		55:45	22 vs. 0
rs3817655	CCL5	Encode	17	16		55:45	4 vs. 9
rs769242	CCNA2	Cancer	4	16		NA	NA
rs1049612	CCND2	Cancer	12	41		50:50	2 vs. 17
rs3217926	CCND2	Cancer	12	41		50:50	0 vs. 16
rs3217933	CCND2	Cancer	12	36		50:50	0 vs. 18
rs9529	CCND3	Cancer	6	44		50:50	7 vs. 8
rs1406	CCNE1	Cancer	19	34		55:45	0 vs. 32
rs4150052	CCNG2	Cancer	4	15		NA	NA
rs8193	CD44	Cancer	11	43		50:50	24 vs. 7
rs9666607	CD44	Cancer	11	28		50:50	0 vs. 12
rs7357	CD59	Cancer	11	33		50:50	12 vs. 4
rs842	CD59	Cancer	11	33		50:50	2 vs. 13
rs1129055	CD86	Cancer	3	39		NA	NA
rs2681417	CD86	Cancer	3	5		NA	NA
rs1871446	CDC2	Cancer	10	29		50:50	6 vs. 1
rs8156	CDC25B	Cancer	20	25		NA	NA
rs2302341	CDC2L5	Cancer	7	7		NA	NA
rs3735135	CDC2L5	Cancer	7	7		NA	NA
rs1801552	CDH1	Encode	16	20		55:45	0 vs. 6
rs1041985	CDH2	Encode	18	38		70:30	3 vs. 20
rs1946482	CDK10	Cancer	16	24		55:45	0 vs. 15
rs2069398	CDK2	Cancer	12	11		NA	NA
rs42039	CDK6	Cancer	7	35		50:50	2 vs. 14
rs8083	CDK9	Cancer	9	33		50:50	1 vs. 20
rs7330	CDKN1B	Cancer	12	41		NA	NA
rs11515	CDKN2A	Cancer	9	17		55:45	12 vs. 2
rs3088440	CDKN2A	Cancer	9	31		60:40	1 vs. 17
rs12855	CDKN2C	Cancer	1	20		55:45	0 vs. 20
rs2231495	CECR1	Encode	22	22		50:50	8 vs. 1
rs1034859	CECR5	Encode	22	37		50:50	6 vs. 13
rs971768	CECR6	Encode	22	17		NA	NA

rs	Gene	Panel	chr	# Hets	Variance & Mean	meanAI	A vs. B
rs6007897	CELSR1	Encode	22	10	High variance	70:30	4 vs. 4
rs2274238	CEP250	Encode	20	7		50:50	0 vs. 2
rs3748433	CEP250	Encode	20	7		NA	NA
rs3818441	CEP250	Encode	20	18		NA	NA
rs2748348	CERK	Encode	22	42		50:50	2 vs. 10
rs8596	CHAF1B	Encode	21	14		NA	NA
rs2272457	CHD2	Cancer	15	38		50:50	2 vs. 6
rs4777755	CHD2	Cancer	15	15		50:50	3 vs. 2
rs506504	CHEK1	Cancer	11	5		NA	NA
rs3741490	CHFR	Cancer	12	36		60:40	1 vs. 30
rs1056825	CHI3L2	Cancer	1	38	High variance	75:25	28 vs. 2
rs1056831	CHI3L2	Cancer	1	39		90:10	37 vs. 2
rs8535	CHI3L2	Cancer	1	38	High variance	75:25	29 vs. 4
rs877292	CHKB	Encode	22	26		NA	NA
rs1043833	CHPF	Encode	2	39		50:50	1 vs. 11
rs1139564	CIITA	Encode	16	18		50:50	6 vs. 2
rs4774	CIITA	Encode	16	29		50:50	0 vs. 7
rs17862175	CLDN12	Encode	7	6		NA	NA
rs917660	CLDN12	Encode	7	15	Mean shifted	NA	NA
rs2834601	CLIC6	Encode	21	8	High variance	NA	NA
rs6517254	CLIC6	Encode	21	24		55:45	2 vs. 10
rs7224	CLK1	Cancer	2	6		NA	NA
rs1061237	COL1A1	Cancer	17	37		50:50	3 vs. 1
rs17334859	CPNE1	Cancer	20	12		60:40	2 vs. 9
rs1043242	CRKL	Cancer	22	32		50:50	0 vs. 1
rs1548410	CRKL	Cancer	22	41		50:50	20 vs. 8
rs2266951	CRKL	Encode	22	33		55:45	25 vs. 2
rs2285547	CRKL	Cancer	22	41		50:50	9 vs. 20
rs737894	CRKL	Encode	22	33		55:45	33 vs. 0
rs2229730	CSK	Cancer	15	4		60:40	0 vs. 4
rs805256	CSNK2B	Cancer	6	36		50:50	0 vs. 24
rs160279	CSPG2	Cancer	5	51		55:45	11 vs. 11
rs1045480	CTBP1	Cancer	4	24		NA	NA
rs6499137	CTCF	Cancer	16	11		NA	NA
rs1059110	CTNNA1	Cancer	5	37		50:50	13 vs. 2
rs2953	CTNNB1	Cancer	3	43		50:50	6 vs. 4
rs17571	CTSD	Encode	11	12		55:45	0 vs. 8
rs8839	CTSD	Cancer	11	24		50:50	2 vs. 8
rs13345	CTSH	Cancer	15	13		65:35	0 vs. 12
rs3129	CTSH	Cancer	15	26		55:45	1 vs. 17
rs1131510	CUL2	Cancer	10	25		50:50	7 vs. 1
rs1804430	CXCL12	Encode	10	6		NA	NA
rs266088	CXCL12	Encode	10	6		NA	NA
rs10336	CXCL9	Cancer	4	11	High variance	70:30	7 vs. 3
rs6571303	CXorf12	Encode	X	8		75:25	4 vs. 3
rs2066852	CYLD	Cancer	16	16		50:50	1 vs. 0
rs10916	CYP1B1	Cancer	2	13	High variance	60:40	6 vs. 3
rs162549	CYP1B1	Cancer	2	22	High variance	65:35	18 vs. 2
rs2855658	CYP1B1	Cancer	2	37	High variance	55:45	19 vs. 5
rs2070540	D21S2056E	Encode	21	7		NA	NA
rs2276246	D21S2056E	Encode	21	9		NA	NA

rs	Gene	Panel	chr	# Hets	Variance & Mean	meanAI	A vs. B
rs1051101	DAD1	Cancer	14	37		50:50	0 vs. 17
rs1803479	DAD1	Cancer	14	9		NA	NA
rs4981429	DAD1	Cancer	14	40		55:45	39 vs. 0
rs1058207	DAP3	Cancer	1	28		50:50	2 vs. 11
rs4933	DAP3	Cancer	1	17		60:40	0 vs. 17
rs3118863	DAPK1	Cancer	9	43		55:45	11 vs. 4
rs697221	DDIT3	Cancer	12	28		50:50	0 vs. 12
rs1049633	DDR1	Cancer	6	16		NA	NA
rs8408	DDR1	Cancer	6	23		55:45	8 vs. 6
rs5750609	DDX17	Cancer	22	27		50:50	9 vs. 4
rs763121	DDX17	Cancer	22	33		50:50	10 vs. 1
rs763121	DDX17	Encode	22	27		50:50	0 vs. 0
rs86796	DDX17	Cancer	22	31		50:50	24 vs. 0
rs1052639	DDX18	Encode	2	10		NA	NA
rs17756426	DDX43	Encode	6	6	High variance	NA	NA
rs311683	DDX43	Encode	6	15	High variance	90:10	10 vs. 5
rs311686	DDX43	Encode	6	17	High variance	85:15	5 vs. 12
rs487728	DDX6	Cancer	11	31		55:45	29 vs. 0
rs488219	DDX6	Cancer	11	31		NA	NA
rs1013062	DEK	Cancer	6	28		55:45	0 vs. 23
rs5998114	DEPDC5	Encode	22	16		NA	NA
rs5998170	DEPDC5	Encode	22	20		NA	NA
rs6623	DGCR2	Encode	22	25		50:50	9 vs. 1
rs1640299	DGCR8	Encode	22	35		50:50	6 vs. 2
rs7374	DHCR24	Cancer	1	48		50:50	1 vs. 12
rs8990	DHCR24	Cancer	1	15		50:50	2 vs. 3
rs2070435	DIP2A	Encode	21	37		50:50	0 vs. 26
rs2078203	DIP2A	Encode	21	33		50:50	1 vs. 2
rs2248636	DIP2A	Encode	21	7		NA	NA
rs2255397	DIP2A	Encode	21	24		50:50	2 vs. 6
rs7283507	DIP2A	Encode	21	19		55:45	5 vs. 6
rs12133766	DISC1	Encode	1	12		NA	NA
rs9090	DKFZP564O0823	Cancer	4	5		55:45	0 vs. 4
rs17863986	DKFZP686A10121	Encode	7	8		55:45	4 vs. 1
rs17863999	DKFZP686A10121	Encode	7	30		50:50	0 vs. 6
rs42663	DKFZP686A10121	Encode	7	29		50:50	8 vs. 0
rs12582	DKFZp762E1312	Encode	2	19		55:45	19 vs. 0
rs702680	DKFZP781I1119	Encode	5	39		55:45	9 vs. 7
rs702681	DKFZP781I1119	Encode	5	32		50:50	0 vs. 26
rs2066575	DLEU1	Cancer	13	27	High variance	55:45	2 vs. 16
rs1047775	DNAJD1	Cancer	13	29		55:45	0 vs. 14
rs14040	DNAL4	Encode	22	22		NA	NA
rs12877	DNASE1L1	Cancer	X	4		80:20	3 vs. 1
rs2070807	DNASE1L1	Encode	X	5	High variance	NA	NA
rs8111085	DNMT1	Cancer	19	16		NA	NA
rs10904889	DNMT2	Cancer	10	14		60:40	0 vs. 12
rs11254401	DNMT2	Cancer	10	15		55:45	0 vs. 11
rs1893963	DSC2	Cancer	18	14	High variance	NA	NA
rs14194	DSCR2	Encode	21	36		50:50	2 vs. 1
rs2245455	DSCR2	Encode	21	31		50:50	4 vs. 1
rs3165	DSCR3	Encode	21	27		55:45	25 vs. 0

rs	Gene	Panel	chr	# Hets	Variance & Mean	meanAI	A vs. B
rs3165	DSCR3	Cancer	21	30		50:50	1 vs. 11
rs2277784	DSCR6	Encode	21	5		NA	NA
rs4712138	DST	Cancer	6	44		50:50	5 vs. 3
rs4715631	DST	Cancer	6	42		50:50	6 vs. 4
rs8630	DUSP18	Encode	22	30		50:50	3 vs. 16
rs11919795	DVL3	Cancer	3	30		55:45	2 vs. 18
rs3749231	DVL3	Cancer	3	38		55:45	0 vs. 26
rs12483205	DYRK1A	Encode	21	29		50:50	11 vs. 0
rs1803439	DYRK1A	Encode	21	34		50:50	10 vs. 0
rs2835726	DYRK1A	Encode	21	40		50:50	8 vs. 0
rs5369	EDN1	Cancer	6	15		NA	NA
rs5370	EDN1	Cancer	6	35		50:50	3 vs. 11
rs5351	EDNRB	Cancer	13	22	High variance	70:30	3 vs. 13
rs1390082	EEF1A1	Encode	6	29		55:45	2 vs. 18
rs6694256	EFNA1	Cancer	1	8		NA	NA
rs4947963	EGFR	Encode	7	19		60:40	4 vs. 8
rs10897533	EHD1	Encode	11	9		55:45	0 vs. 8
rs1211284	EHD1	Encode	11	9		50:50	2 vs. 0
rs12573	EHD1	Encode	11	16		50:50	5 vs. 2
rs2157472	EIF3S6IP	Encode	22	32		50:50	1 vs. 8
rs9466	EIF3S6IP	Encode	22	37		60:40	37 vs. 0
rs4820972	EIF4ENIF1	Encode	22	8		NA	NA
rs5753627	EIF4ENIF1	Encode	22	13		NA	NA
rs3844075	ELL3	Encode	15	20		55:45	3 vs. 7
rs300239	ENC1	Cancer	5	27		65:35	1 vs. 23
rs442425	ENC1	Cancer	5	16		NA	NA
rs1046088	EP300	Encode	22	8		NA	NA
rs2267424	EP300	Encode	22	30		55:45	2 vs. 15
rs17567	EPS15	Cancer	1	18		50:50	1 vs. 8
rs7308	EPS15	Cancer	1	18		65:35	0 vs. 18
rs8664	EPS8	Cancer	12	9		50:50	2 vs. 2
rs11615	ERCC1	Cancer	19	35		50:50	3 vs. 17
rs1052559	ERCC2	Cancer	19	35		55:45	1 vs. 28
rs13181	ERCC2	Cancer	19	35		55:45	1 vs. 24
rs17655	ERCC5	Cancer	13	33		55:45	0 vs. 28
rs2227869	ERCC5	Cancer	13	11		NA	NA
rs2228528	ERCC6	Cancer	10	31		55:45	9 vs. 5
rs2229760	ERCC6	Cancer	10	27		50:50	1 vs. 5
rs3088093	ERN1	Cancer	17	19		NA	NA
rs4937333	ETS1	Cancer	11	35		50:50	3 vs. 14
rs8705	ETS1	Cancer	11	26		60:40	0 vs. 25
rs1051420	ETS2	Cancer	21	33		50:50	8 vs. 1
rs11254	ETS2	Cancer	21	42		50:50	10 vs. 4
rs1209953	ETS2	Encode	21	35		55:45	9 vs. 9
rs461155	ETS2	Encode	21	16		NA	NA
rs1058028	ETV6	Cancer	12	34		50:50	28 vs. 0
rs7505	EVI2A	Cancer	17	39		50:50	14 vs. 2
rs2042456	EXOC4	Encode	7	34		50:50	0 vs. 22
rs1801719	F2R	Cancer	5	31		60:40	26 vs. 0
rs1050705	F8	Encode	X	12	High variance	80:20	8 vs. 3
rs1800291	F8	Encode	X	6		85:15	2 vs. 4

rs	Gene	Panel	chr	# Hets	Variance & Mean	meanAI	A vs. B
rs7647987	FANCD2	Cancer	3	35		55:45	3 vs. 19
rs4447177	FANCF	Cancer	11	10		55:45	4 vs. 1
rs848291	FANCL	Cancer	2	41		50:50	7 vs. 24
rs1468063	FAS	Cancer	10	11		NA	NA
rs710174	FBXO7	Encode	22	32		50:50	2 vs. 7
rs8140067	FBXO7	Encode	22	21		50:50	0 vs. 1
rs11264793	FCRL3	Encode	1	24		70:30	6 vs. 8
rs6691569	FCRL3	Encode	1	12		75:25	9 vs. 3
rs4246215	FEN1	Cancer	11	39		65:35	0 vs. 39
rs1133392	FER	Cancer	5	19		NA	NA
rs2229085	FER	Cancer	5	36		50:50	1 vs. 8
rs919771	FER	Cancer	5	23		50:50	3 vs. 2
rs1476217	FGF2	Cancer	4	43	High variance	60:40	8 vs. 23
rs3747676	FGF2	Cancer	4	42	High variance	55:45	8 vs. 9
rs546782	FGF9	Cancer	13	10	High variance	NA	NA
rs1076890	FGFR4	Cancer	5	5		50:50	0 vs. 0
rs9018	FHL1	Cancer	X	4		85:15	2 vs. 2
rs1046330	FLJ10996	Encode	2	38		50:50	1 vs. 1
rs10490631	FLJ10996	Encode	2	6		NA	NA
rs17512204	FLJ10996	Encode	2	13		NA	NA
rs1048310	FLJ20232	Encode	22	32		50:50	0 vs. 11
rs4821903	FLJ20232	Encode	22	37		50:50	3 vs. 9
rs9615938	FLJ20699	Encode	22	21		55:45	1 vs. 15
rs1029365	FLJ21062	Encode	7	30		60:40	1 vs. 16
rs3761805	FLJ21062	Encode	7	25		NA	NA
rs1053001	FLJ21125	Encode	22	21		50:50	1 vs. 7
rs2277841	FLJ23322	Encode	22	27		55:45	0 vs. 19
rs3747163	FLJ23322	Encode	22	17		NA	NA
rs137794	FLJ23588	Encode	22	11		NA	NA
rs2235432	FLJ33814	Encode	22	7		NA	NA
rs5762795	FLJ33814	Encode	22	34		50:50	1 vs. 24
rs2240176	FLJ35801	Encode	22	18		95:05	4 vs. 12
rs1049739	FOSB	Cancer	19	30		55:45	1 vs. 15
rs9471607	FOXP4	Encode	6	11		55:45	11 vs. 0
rs792310	FPGT	Cancer	1	50		55:45	0 vs. 49
rs1064261	FRAP1	Cancer	1	29		50:50	1 vs. 1
rs495565	FRK	Cancer	6	38		50:50	9 vs. 5
rs580396	FRK	Cancer	6	38		55:45	0 vs. 16
rs1057227	FRS3	Encode	6	33		50:50	2 vs. 0
rs6810	FVT1	Cancer	18	33		50:50	17 vs. 1
rs1050757	G6PD	Encode	X	6	Mean shifted	90:10	6 vs. 0
rs3813455	GAB3	Encode	X	10	High variance	85:15	4 vs. 5
rs7283354	GART	Encode	21	36		50:50	5 vs. 7
rs8971	GART	Encode	21	35		50:50	14 vs. 0
rs9984077	GART	Encode	21	19		50:50	4 vs. 1
rs1047365	GAS7	Cancer	17	40		50:50	14 vs. 7
rs2240739	GAS7	Cancer	17	36		55:45	5 vs. 11
rs2270121	GAS7	Cancer	17	38		60:40	1 vs. 26
rs13911	GCAT	Encode	22	31		50:50	1 vs. 12
rs989099	GCC1	Encode	7	28		50:50	27 vs. 0
rs1325432	GFI1	Cancer	1	11		50:50	0 vs. 3

rs	Gene	Panel	chr	# Hets	Variance & Mean	meanAI	A vs. B
rs4970714	GFI1	Cancer	1	43		55:45	14 vs. 10
rs12473304	GFPT1	Encode	2	20		50:50	10 vs. 0
rs2667	GFPT1	Encode	2	38		50:50	1 vs. 5
rs2269547	GGA1	Encode	22	12		50:50	5 vs. 0
rs2228226	GLI1	Cancer	12	38		55:45	3 vs. 15
rs7121	GNAS	Cancer	20	38		50:50	1 vs. 6
rs9612234	GNAZ	Encode	22	16		65:35	2 vs. 8
rs3752174	GNG7	Cancer	19	29		50:50	4 vs. 3
rs712723	GRM8	Encode	7	35		55:45	21 vs. 1
rs1571858	GSTM3	Encode	1	17	High variance	75:25	8 vs. 4
rs4891	GSTP1	Cancer	11	36		50:50	3 vs. 0
rs947894	GSTP1	Cancer	11	36		50:50	5 vs. 14
rs140054	GTSE1	Encode	22	10		NA	NA
rs6008622	GTSE1	Encode	22	21		50:50	0 vs. 7
rs6008729	GTSE1	Encode	22	21		55:45	7 vs. 1
rs9615947	GTSE1	Encode	22	20		NA	NA
rs9530	GUSB	Cancer	7	38		50:50	3 vs. 9
rs13385	HBEGF	Cancer	5	14		NA	NA
rs7268	HBEGF	Cancer	5	40		55:45	8 vs. 11
rs2276881	HD	Encode	4	11		NA	NA
rs362272	HD	Encode	4	27		50:50	18 vs. 1
rs362303	HD	Encode	4	27		NA	NA
rs362331	HD	Encode	4	33		50:50	0 vs. 17
rs363125	HD	Encode	4	24		NA	NA
rs2408874	HDAC7A	Cancer	12	28		55:45	1 vs. 23
rs7418	HDAC7A	Cancer	12	30		50:50	9 vs. 5
rs2023938	HDAC9	Cancer	7	18		NA	NA
rs801524	HDAC9	Cancer	7	20		50:50	0 vs. 3
rs9842	HDGF	Cancer	1	6		NA	NA
rs11549465	HIF1A	Cancer	14	10		50:50	5 vs. 0
rs689797	HISPPD2A	Encode	15	36		55:45	9 vs. 9
rs11244	HLA	Cancer	6	28		55:45	0 vs. 24
rs16871435	HLA	Cancer	6	14		70:30	0 vs. 14
rs2070121	HLA	Cancer	6	18		50:50	1 vs. 9
rs7194	HLA	Cancer	6	42		50:50	6 vs. 0
rs7905	HLA	Cancer	6	8		50:50	1 vs. 2
rs8084	HLA	Cancer	6	42		50:50	19 vs. 4
rs8807	HLA	Cancer	6	15		60:40	14 vs. 0
rs9273960	HLA	Cancer	6	26		55:45	0 vs. 22
rs9277534	HLA	Cancer	6	38		50:50	21 vs. 3
rs9277535	HLA	Cancer	6	37		50:50	12 vs. 6
rs1053593	HMG2L1	Encode	22	35		50:50	0 vs. 24
rs299290	HMMR	Cancer	5	37		50:50	1 vs. 8
rs17759	HNRPAB	Cancer	5	22		50:50	11 vs. 2
rs3747129	HPS4	Encode	22	10		60:40	0 vs. 10
rs3747134	HPS4	Encode	22	15		55:45	0 vs. 15
rs3752589	HPS4	Encode	22	8		NA	NA
rs722997	HPS4	Encode	22	8		NA	NA
rs3747011	HRMT1L1	Encode	21	7		NA	NA
rs1061810	HSD17B12	Cancer	11	34		55:45	34 vs. 0
rs5749426	HSPC117	Encode	22	40		50:50	1 vs. 17

rs	Gene	Panel	chr	# Hets	Variance & Mean	meanAI	A vs. B
rs1633445	HTF9C	Encode	22	21		55:45	0 vs. 17
rs3093032	ICAM1	Cancer	19	24		50:50	4 vs. 1
rs3746963	ICOSLG	Encode	21	23		50:50	2 vs. 4
rs4819388	ICOSLG	Encode	21	25		55:45	18 vs. 0
rs2257167	IFNAR1	Encode	21	21		50:50	5 vs. 4
rs2834202	IFNAR1	Encode	21	30		50:50	0 vs. 16
rs2856968	IFNAR1	Encode	21	34		60:40	0 vs. 27
rs11088247	IFNAR2	Encode	21	31		55:45	0 vs. 29
rs4986956	IFNAR2	Encode	21	13		NA	NA
rs11914	IFNGR1	Cancer	6	19		NA	NA
rs1059293	IFNGR2	Cancer	21	44		55:45	34 vs. 1
rs6214	IGF1	Cancer	12	31	High variance	60:40	13 vs. 11
rs6219	IGF1	Cancer	12	6		NA	NA
rs998075	IGF2R	Cancer	6	49		50:50	0 vs. 18
rs6670	IGFBP3	Cancer	7	11		NA	NA
rs4445669	IGSF4	Cancer	11	40	High variance	70:30	5 vs. 23
rs3024496	IL10	Cancer	1	30		50:50	3 vs. 8
rs3024498	IL10	Cancer	1	19		55:45	10 vs. 2
rs3024498	IL10	Encode	1	18		50:50	0 vs. 3
rs2284552	IL10RB	Encode	21	31		NA	NA
rs568408	IL12A	Cancer	3	9		50:50	0 vs. 0
rs1368439	IL12B	Cancer	5	23		NA	NA
rs1368439	IL12B	Encode	5	10		60:40	2 vs. 16
rs1131445	IL16	Cancer	15	33		NA	NA
rs3726	IL16	Cancer	15	49		50:50	3 vs. 17
rs859	IL16	Cancer	15	28		50:50	0 vs. 12
rs1025689	IL17RB	Cancer	3	29		65:35	1 vs. 19
rs1043261	IL17RB	Cancer	3	11		NA	NA
rs949323	IL18BP	Cancer	11	6		NA	NA
rs1304037	IL1A	Cancer	2	26	High variance	75:25	8 vs. 12
rs17561	IL1A	Cancer	2	21	High variance	75:25	8 vs. 8
rs1071676	IL1B	Cancer	2	29	High variance	65:35	8 vs. 14
rs1043388	ILK	Cancer	11	28		55:45	0 vs. 20
rs1043390	ILK	Cancer	11	30		50:50	0 vs. 22
rs2292195	ILK	Cancer	11	27		50:50	2 vs. 6
rs1053474	IMPACT	Cancer	18	31		60:40	8 vs. 21
rs677688	IMPACT	Cancer	18	7		65:35	1 vs. 5
rs1061386	ING1	Cancer	13	35		55:45	0 vs. 33
rs2962	INSR	Cancer	19	5		NA	NA
rs1059701	IRAK1	Cancer	X	14	High variance	85:15	6 vs. 8
rs4251545	IRAK4	Cancer	12	6		NA	NA
rs2289046	IRS2	Cancer	13	22		55:45	6 vs. 6
rs2289047	IRS2	Cancer	13	30		55:45	2 vs. 13
rs1316757	ITGB1	Cancer	10	15		NA	NA
rs10593	ITGB1BP1	Cancer	2	23	High variance	65:35	10 vs. 10
rs4798	ITGB1BP1	Cancer	2	34		60:40	2 vs. 25
rs235326	ITGB2	Encode	21	17		NA	NA
rs684	ITGB2	Encode	21	33		50:50	1 vs. 13
rs1900941	ITPR2	Cancer	12	42		50:50	9 vs. 2
rs2291264	ITPR2	Cancer	12	40		55:45	4 vs. 27
rs2229634	ITPR3	Cancer	6	40		60:40	3 vs. 25

rs	Gene	Panel	chr	# Hets	Variance & Mean	meanAI	A vs. B
rs749338	ITPR3	Cancer	6	23		60:40	1 vs. 8
rs7828	JAG1	Cancer	20	21		50:50	8 vs. 6
rs2230724	JAK2	Cancer	9	40		55:45	2 vs. 23
rs7048717	JAK2	Cancer	9	40		55:45	1 vs. 24
rs3008	JAK3	Cancer	19	41		50:50	4 vs. 7
rs2829877	JAM2	Encode	21	17		60:40	4 vs. 7
rs4816260	JAM2	Encode	21	25		55:45	12 vs. 5
rs4980809	JARID1A	Cancer	12	24		50:50	5 vs. 4
rs3827358	JOSD1	Encode	22	11		50:50	0 vs. 1
rs4820345	JOSD1	Encode	22	25		55:45	0 vs. 18
rs10478	KATNAL1	Encode	13	29		50:50	6 vs. 2
rs2230033	KCNJ15	Encode	21	33		60:40	4 vs. 11
rs10798	KCNQ1	Cancer	11	31	High variance	75:25	15 vs. 13
rs1543621	KCNQ5	Encode	6	41		50:50	0 vs. 2
rs7282122	KIAA0179	Encode	21	42		50:50	1 vs. 21
rs3747113	KIAA0376	Encode	22	26		50:50	1 vs. 6
rs1665105	KIAA0828	Cancer	7	30		55:45	8 vs. 10
rs4731575	KIAA0828	Cancer	7	30		50:50	2 vs. 13
rs10047459	KIAA0999	Encode	11	10		NA	NA
rs12225230	KIAA0999	Encode	11	18		55:45	0 vs. 16
rs11242126	KIF3A	Encode	5	14		55:45	0 vs. 10
rs140519	KLHDC7B	Encode	22	30		70:30	1 vs. 28
rs8353	KLHL22	Encode	22	30		55:45	0 vs. 18
rs1137282	KRAS	Cancer	12	37		50:50	6 vs. 4
rs12587	KRAS	Cancer	12	46		50:50	21 vs. 2
rs13096	KRAS	Cancer	12	45		60:40	0 vs. 38
rs9266	KRAS	Cancer	12	46		50:50	20 vs. 5
rs7561	LAMB1	Cancer	7	21	High variance	70:30	10 vs. 3
rs16992034	LARGE	Encode	22	9		NA	NA
rs86487	LARGE	Encode	22	31		55:45	7 vs. 9
rs708459	LEAP-2	Encode	5	40		55:45	0 vs. 27
rs1050527	LENG4	Encode	19	10		NA	NA
rs10131	LIG4	Cancer	13	10	High variance	60:40	0 vs. 10
rs1805388	LIG4	Cancer	13	24		50:50	3 vs. 7
rs8101605	LILRB1	Encode	19	17		55:45	14 vs. 1
rs3745410	LILRB3	Encode	19	5	High variance	NA	NA
rs17207369	LILRP2	Encode	19	18		65:35	4 vs. 12
rs4141404	LIMK2	Encode	22	30		50:50	7 vs. 4
rs1051643	LMNB1	Cancer	5	35		50:50	4 vs. 6
rs3749830	LMNB1	Cancer	5	11		NA	NA
rs2298428	LOC150223	Encode	22	19		NA	NA
rs2269729	LOC168850	Encode	7	28		65:35	0 vs. 27
rs6518322	LOC284837	Encode	21	29		60:40	8 vs. 12
rs41173	LOC400924	Encode	22	31		55:45	8 vs. 10
rs1045523	LOC402055	Encode	22	15		50:50	1 vs. 0
rs4820682	LOC402055	Encode	22	15		55:45	1 vs. 11
rs5761561	LOC402055	Encode	22	22		NA	NA
rs10071051	LOC441108	Encode	5	39		55:45	4 vs. 29
rs2522050	LOC441108	Encode	5	26		50:50	0 vs. 15
rs2286442	LOC550631	Encode	22	12		NA	NA
rs9624806	LRP5L	Encode	22	15		55:45	5 vs. 3

rs	Gene	Panel	chr	# Hets	Variance & Mean	meanAI	A vs. B
rs9071	LRRC14	Cancer	8	34		50:50	2 vs. 20
rs1048326	LRRK1	Cancer	15	9		NA	NA
rs2089910	LSP1	Encode	11	33		50:50	0 vs. 3
rs5013907	LSP1	Encode	11	5		NA	NA
rs548195	LSP1	Encode	11	20		NA	NA
rs2254522	LSS	Encode	21	23		NA	NA
rs2839158	LSS	Encode	21	27		55:45	1 vs. 26
rs2968	LSS	Encode	21	36		50:50	0 vs. 17
rs914247	LSS	Encode	21	34		55:45	0 vs. 21
rs2239704	LTA	Cancer	6	39		55:45	0 vs. 19
rs2857713	LTA	Cancer	6	39		60:40	0 vs. 35
rs2857713	LTA	Encode	6	35		55:45	0 vs. 34
rs7289487	LZTR1	Encode	22	25		55:45	1 vs. 6
rs1059442	MALT1	Cancer	18	16		NA	NA
rs4792219	MAP2K4	Cancer	17	46		65:35	0 vs. 46
rs2072074	MAP2K6	Cancer	17	16		50:50	1 vs. 3
rs832582	MAP3K1	Cancer	5	19		55:45	0 vs. 11
rs832583	MAP3K1	Cancer	5	19		50:50	2 vs. 5
rs1042058	MAP3K8	Cancer	10	43		55:45	0 vs. 32
rs958	MAPK10	Cancer	4	28		60:40	1 vs. 14
rs2272857	MAPK12	Encode	22	28		50:50	7 vs. 9
rs3804451	MAPK14	Cancer	6	11		NA	NA
rs8510	MAPK14	Cancer	6	6		50:50	0 vs. 3
rs9605	MAPK9	Cancer	5	36		55:45	1 vs. 30
rs3786254	MBD2	Cancer	18	34		50:50	2 vs. 12
rs7614	MBD2	Cancer	18	42		50:50	16 vs. 6
rs7914	MCAM	Cancer	11	31	High variance	60:40	3 vs. 14
rs878471	MCL1	Cancer	1	32		50:50	11 vs. 1
rs11702450	MCM3AP	Encode	21	39		60:40	2 vs. 35
rs2839165	MCM3AP	Encode	21	22		50:50	0 vs. 6
rs2839181	MCM3AP	Encode	21	40		60:40	1 vs. 20
rs9975588	MCM3AP	Encode	21	37		50:50	1 vs. 5
rs4645824	MCM5	Encode	22	13		NA	NA
rs3087350	MCM6	Cancer	2	9		NA	NA
rs1055521	MDFIC	Encode	7	24		50:50	0 vs. 19
rs2709507	MDFIC	Encode	7	6		NA	NA
rs769412	MDM2	Cancer	12	6		NA	NA
rs4245739	MDM4	Cancer	1	34		50:50	1 vs. 9
rs4252745	MDM4	Cancer	1	42		50:50	1 vs. 1
rs2734647	MECP2	Cancer	X	12	High variance	85:15	6 vs. 6
rs2734647	MECP2	Encode	X	10		85:15	4 vs. 6
rs10863	MEST	Cancer	7	29	High variance	70:30	18 vs. 5
rs16965120	MFAP1	Encode	15	7		NA	NA
rs2228368	MFAP1	Encode	15	7		NA	NA
rs11216129	MGC13125	Encode	11	15		NA	NA
rs4615	MGC17330	Encode	22	9		NA	NA
rs4820961	MGC17330	Encode	22	21		55:45	2 vs. 13
rs2257505	MGC33648	Encode	5	29		60:40	0 vs. 20
rs33321	MGC33648	Encode	5	39		55:45	2 vs. 19
rs40497	MGC33648	Encode	5	37		55:45	5 vs. 11
rs16989427	MGC50372	Encode	22	7		NA	NA

rs	Gene	Panel	chr	# Hets	Variance & Mean	meanAI	A vs. B
rs1803965	MGMT	Cancer	10	5		70:30	3 vs. 1
rs4821944	MKL1	Encode	22	30		55:45	0 vs. 18
rs1050629	MLF2	Cancer	12	9		NA	NA
rs2302371	MLF2	Cancer	12	23		60:40	0 vs. 17
rs6906754	MLLT4	Cancer	6	9		NA	NA
rs10502001	MMP7	Cancer	11	19	High variance	55:45	2 vs. 3
rs14983	MMP7	Cancer	11	22	High variance	55:45	5 vs. 6
rs2072132	MORC2	Encode	22	32		55:45	17 vs. 0
rs737924	MORC2	Encode	22	31		50:50	2 vs. 7
rs15017	MOXD1	Encode	6	6	High variance	60:40	1 vs. 3
rs3734744	MOXD1	Encode	6	11		60:40	7 vs. 2
rs2155209	MRE11A	Cancer	11	32		55:45	0 vs. 15
rs12812	MRPL23	Encode	11	15		50:50	1 vs. 3
rs1135638	MRPL39	Encode	21	25		50:50	9 vs. 0
rs3178972	MRPL39	Encode	21	25		55:45	0 vs. 20
rs13052524	MRPS6	Encode	21	19		NA	NA
rs7115	MRPS6	Encode	21	39		50:50	19 vs. 0
rs1805355	MSH3	Cancer	5	5		50:50	0 vs. 1
rs184967	MSH3	Cancer	5	8		NA	NA
rs1800935	MSH6	Cancer	2	36		50:50	1 vs. 5
rs13815	MT	Encode	22	29		55:45	0 vs. 22
rs2066910	MT	Encode	22	25		60:40	0 vs. 20
rs1801131	MTHFR	Cancer	1	30		55:45	2 vs. 14
rs16988135	MTMR3	Encode	22	24		NA	NA
rs3788418	MTMR3	Encode	22	17		60:40	17 vs. 0
rs4509087	MTO1	Encode	6	17		50:50	0 vs. 4
rs1050008	MX1	Encode	21	11		NA	NA
rs2070229	MX1	Encode	21	33		50:50	26 vs. 2
rs461093	MX1	Encode	21	29		55:45	0 vs. 18
rs467960	MX1	Encode	21	35		50:50	4 vs. 3
rs14401	MXI1	Cancer	10	42		55:45	2 vs. 25
rs17658	MXI1	Cancer	10	38		50:50	4 vs. 12
rs2070583	MYC	Cancer	8	11		NA	NA
rs2481	MYH9	Encode	22	16		NA	NA
rs5756130	MYH9	Encode	22	9		NA	NA
rs735854	MYH9	Encode	22	29		50:50	2 vs. 4
rs1061302	NBS1	Cancer	8	38		50:50	1 vs. 20
rs1063045	NBS1	Cancer	8	38		50:50	3 vs. 27
rs2072712	NCF4	Encode	22	9		NA	NA
rs1131171	NCL	Cancer	2	20		50:50	1 vs. 5
rs2076546	NCOA3	Cancer	20	8		50:50	1 vs. 2
rs2230782	NCOA3	Cancer	20	19		50:50	3 vs. 3
rs9394782	NCR2	Encode	6	15		75:25	0 vs. 11
rs1801311	NDUFA6	Encode	22	37		50:50	10 vs. 2
rs4147641	NDUFA6	Encode	22	37		50:50	21 vs. 3
rs7245	NDUFA6	Encode	22	37		50:50	0 vs. 12
rs2839600	NDUFV3	Encode	21	17		60:40	0 vs. 16
rs4148972	NDUFV3	Encode	21	8		NA	NA
rs165602	NEFH	Encode	22	6		70:30	4 vs. 1
rs1801052	NF1	Cancer	17	41		50:50	3 vs. 2
rs1008515	NF2	Cancer	22	9		NA	NA

rs	Gene	Panel	chr	# Hets	Variance & Mean	meanAI	A vs. B
rs1008515	NF2	Encode	22	27		NA	NA
rs1057157	NFAM1	Encode	22	41		50:50	9 vs. 11
rs1609993	NFKB1	Cancer	4	5		NA	NA
rs11574851	NFKB2	Cancer	10	6		NA	NA
rs10782383	NFKBIA	Cancer	14	37		50:50	14 vs. 18
rs6121023	NFS1	Encode	20	14		50:50	4 vs. 0
rs132806	NHP2L1	Encode	22	33		50:50	0 vs. 5
rs3213190	NID	Cancer	1	17		NA	NA
rs469363	NIPSNAP1	Encode	22	21		55:45	2 vs. 6
rs7609	NIPSNAP1	Encode	22	26		55:45	1 vs. 18
rs699780	NOTCH2	Cancer	1	11		55:45	9 vs. 0
rs835575	NOTCH2	Cancer	1	8		60:40	0 vs. 7
rs835576	NOTCH2	Cancer	1	10		55:45	5 vs. 0
rs1800566	NQO1	Cancer	16	26		55:45	7 vs. 7
rs2288539	NR2F6	Cancer	19	18	Mean shifted	NA	NA
rs14804	NRAS	Cancer	1	17		50:50	0 vs. 0
rs1056947	NRIP1	Encode	21	34		55:45	12 vs. 8
rs17066360	NUFIP1	Cancer	13	5		NA	NA
rs2673084	NUP50	Encode	22	39		60:40	1 vs. 37
rs2459216	OAT	Cancer	10	6	High variance	NA	NA
rs10742622	OR51B2	Encode	11	9		NA	NA
rs7934354	OR52H1	Encode	11	9		NA	NA
rs7397032	OR56B1	Encode	11	17		NA	NA
rs11681	PALM2	Cancer	9	12		NA	NA
rs1983609	PARVB	Encode	22	22		50:50	0 vs. 12
rs4819157	PCBP3	Encode	21	6	High variance	NA	NA
rs1042531	PCK1	Encode	20	9		65:35	6 vs. 1
rs2073376	PCNT	Encode	21	35		55:45	2 vs. 16
rs2249057	PCNT	Encode	21	36		55:45	0 vs. 29
rs2839245	PCNT	Encode	21	19		NA	NA
rs6518291	PCNT	Encode	21	30		55:45	21 vs. 0
rs2066954	PDCD2	Cancer	6	45		50:50	10 vs. 3
rs8770	PDCD2	Cancer	6	49		55:45	45 vs. 0
rs702530	PDE4D	Encode	5	42		55:45	2 vs. 28
rs10163054	PDIA3	Encode	15	7		NA	NA
rs1053492	PDIA3	Encode	15	36		50:50	0 vs. 18
rs3087657	PDIA3	Encode	15	37		50:50	6 vs. 0
rs2812	PECAM1	Cancer	17	30		55:45	2 vs. 13
rs6809	PECAM1	Cancer	17	37		50:50	6 vs. 5
rs13073	PEG10	Cancer	7	35	High variance	95:05	21 vs. 14
rs3750105	PEG10	Cancer	7	8	High variance	NA	NA
rs7810469	PEG10	Cancer	7	12	High variance	95:05	5 vs. 7
rs464385	PEX26	Encode	22	38		50:50	2 vs. 9
rs1057034	PFKL	Encode	21	40		50:50	5 vs. 2
rs11771034	PFTK1	Encode	7	18		50:50	2 vs. 4
rs994511	PFTK1	Encode	7	39		50:50	8 vs. 10
rs1065201	PGEA1	Encode	22	25		50:50	0 vs. 3
rs5757213	PGEA1	Encode	22	25		50:50	3 vs. 3
rs1049620	PHB	Cancer	17	18		50:50	3 vs. 8
rs3729680	PIK3CA	Cancer	3	5		NA	NA
rs3730089	PIK3R1	Cancer	5	38		50:50	1 vs. 9

rs	Gene	Panel	chr	# Hets	Variance & Mean	meanAI	A vs. B
rs706713	PIK3R1	Cancer	5	29		50:50	7 vs. 4
rs1056847	PIK4CB	Encode	1	34		50:50	1 vs. 2
rs1044085	PISD	Encode	22	18		55:45	1 vs. 16
rs2728121	PKD2	Cancer	4	9		50:50	1 vs. 1
rs378528	PKNOX1	Encode	21	31		50:50	3 vs. 18
rs413621	PKNOX1	Encode	21	14		55:45	0 vs. 13
rs2076684	PLAGL1	Cancer	6	12		NA	NA
rs9373409	PLAGL1	Cancer	6	43		55:45	4 vs. 15
rs6061216	PLAGL2	Cancer	20	42		50:50	0 vs. 19
rs2302524	PLAUR	Cancer	19	15		NA	NA
rs2075760	PLSCR3	Cancer	17	19		65:35	3 vs. 9
rs3817405	PLXDC2	Cancer	10	12	High variance	75:25	2 vs. 8
rs5945431	PLXNA3	Encode	X	9	High variance	85:15	7 vs. 2
rs5987266	PLXNA3	Encode	X	10	High variance	85:15	7 vs. 2
rs9479	PML	Cancer	15	36		55:45	31 vs. 0
rs2066459	PMS1	Cancer	2	8		NA	NA
rs2297285	POFUT2	Encode	21	28		50:50	6 vs. 2
rs2838859	POFUT2	Encode	21	40		50:50	17 vs. 3
rs216590	POLR3K	Encode	16	32		50:50	10 vs. 6
rs1053046	PPARD	Cancer	6	6		NA	NA
rs3856806	PPARG	Encode	3	5	High variance	65:35	2 vs. 1
rs1042040	PPAT	Cancer	4	51		50:50	0 vs. 40
rs3733326	PPAT	Cancer	4	6		NA	NA
rs1103229	PPIL2	Encode	22	26		60:40	0 vs. 22
rs10413435	PPP2R1A	Cancer	19	9		NA	NA
rs2480452	PPP2R4	Encode	9	7		NA	NA
rs11264542	PRCC	Cancer	1	23		55:45	5 vs. 11
rs1203651	PRDM2	Cancer	1	15		55:45	0 vs. 12
rs3795753	PRDM2	Cancer	1	11		50:50	4 vs. 1
rs6958	PRKAR1A	Cancer	17	28		55:45	28 vs. 0
rs8905	PRKAR1A	Cancer	17	12		55:45	10 vs. 0
rs1051992	PRKCDBP	Cancer	11	37		60:40	4 vs. 16
rs2239822	PSCD4	Encode	22	33		55:45	10 vs. 11
rs7172	PSMB4	Encode	1	27		50:50	0 vs. 23
rs2295997	PTCH2	Cancer	1	22		NA	NA
rs17197	PTGER2	Encode	14	10		70:30	8 vs. 0
rs2206593	PTGS2	Cancer	1	10	High variance	60:40	1 vs. 5
rs5275	PTGS2	Cancer	1	41		60:40	4 vs. 17
rs7460	PTK2	Cancer	8	42		50:50	4 vs. 10
rs1030526	PTK2B	Cancer	8	40		50:50	10 vs. 17
rs16997057	PTPNS1	Cancer	20	26		65:35	0 vs. 26
rs11312	PTTG1IP	Encode	21	36		50:50	3 vs. 17
rs2020945	PWP2H	Encode	21	18		NA	NA
rs4767884	PXN	Cancer	12	26		70:30	2 vs. 23
rs12166968	RAC2	Encode	22	5		NA	NA
rs2239774	RAC2	Encode	22	17		NA	NA
rs8240	RAD23A	Cancer	19	9		NA	NA
rs12593359	RAD51	Cancer	15	44		60:40	5 vs. 33
rs1051672	RAD52	Cancer	12	25		50:50	7 vs. 6
rs7301931	RAD52	Cancer	12	45		50:50	6 vs. 14
rs7310449	RAD52	Cancer	12	47		55:45	0 vs. 36

rs	Gene	Panel	chr	# Hets	Variance & Mean	meanAI	A vs. B
rs1048771	RAD54L	Cancer	1	16		NA	NA
rs1051208	RAF1	Cancer	3	11		NA	NA
rs1065518	RALB	Cancer	2	25		50:50	1 vs. 2
rs14035	RAN	Cancer	12	23		55:45	1 vs. 11
rs1953	RANGAP1	Encode	22	19		50:50	1 vs. 3
rs6573	RAP1A	Cancer	1	9		NA	NA
rs12873919	RAP2A	Cancer	13	40		50:50	7 vs. 1
rs2389910	RAP2A	Cancer	13	38		50:50	0 vs. 0
rs2073498	RASSF1	Cancer	3	23		55:45	0 vs. 11
rs11240353	RBBP5	Cancer	1	16		55:45	0 vs. 13
rs7593	RBBP6	Cancer	16	32		50:50	3 vs. 1
rs10748	RBL2	Cancer	16	44		50:50	17 vs. 0
rs3929	RBL2	Cancer	16	44		50:50	0 vs. 32
rs2822445	RBM11	Encode	21	38		60:40	6 vs. 19
rs6060536	RBM12	Encode	20	14		50:50	0 vs. 11
rs1061474	RBM5	Cancer	3	51		50:50	3 vs. 14
rs522162	RDBP	Cancer	6	7		NA	NA
rs1061627	RECQL	Cancer	12	26		50:50	4 vs. 0
rs1047148	RFC4	Cancer	3	34		50:50	11 vs. 7
rs7552906	RFX5	Encode	1	30		55:45	0 vs. 25
rs3448	RHOA	Cancer	3	44		50:50	12 vs. 23
rs2245466	RHOH	Cancer	4	33		50:50	1 vs. 9
rs1022477	RIBC2	Encode	22	26		70:30	0 vs. 20
rs3212254	RIPK3	Cancer	14	10		NA	NA
rs10889205	RLF	Cancer	1	27		55:45	0 vs. 18
rs3812471	RNF139	Cancer	8	31		50:50	9 vs. 0
rs5749222	RNF185	Encode	22	24		50:50	2 vs. 9
rs8595	RNF185	Encode	22	20		60:40	0 vs. 20
rs12727	RPA1	Cancer	17	15		55:45	0 vs. 14
rs1131274	RYK	Cancer	3	23		65:35	0 vs. 23
rs1047325	S100A2	Cancer	1	10		50:50	0 vs. 0
rs7281104	SAMSN1	Encode	21	18		50:50	5 vs. 3
rs8142229	SAPS2	Encode	22	35		55:45	1 vs. 15
rs503068	SEC63	Encode	6	21		55:45	21 vs. 0
rs642954	SEC63	Encode	6	5		NA	NA
rs17072102	SERPINB10	Encode	18	23		55:45	3 vs. 11
rs724558	SERPINB10	Encode	18	23		70:30	0 vs. 23
rs8097425	SERPINB10	Encode	18	24		65:35	0 vs. 24
rs963075	SERPINB10	Encode	18	32	High variance	60:40	11 vs. 15
rs2288287	SERPINB2	Encode	18	6		NA	NA
rs6098	SERPINB2	Encode	18	12		60:40	5 vs. 3
rs6102	SERPINB2	Encode	18	18		50:50	3 vs. 1
rs6104	SERPINB2	Cancer	18	19	High variance	65:35	2 vs. 15
rs3169983	SERPINB8	Encode	18	6		NA	NA
rs268687	SERTAD1	Encode	19	37		50:50	3 vs. 9
rs523200	SF1	Encode	11	18		50:50	0 vs. 11
rs10376	SF3A1	Encode	22	32		50:50	0 vs. 7
rs2006771	SFI1	Encode	22	40		50:50	0 vs. 20
rs5753700	SFI1	Encode	22	6		NA	NA
rs1522	SH3BGR	Encode	21	5		NA	NA
rs8190	SIAH1	Cancer	16	12		50:50	5 vs. 5

rs	Gene	Panel	chr	# Hets	Variance & Mean	meanAI	A vs. B
rs2258772	SIN3B	Cancer	19	49		50:50	5 vs. 15
rs3184577	SIN3B	Cancer	19	10		50:50	1 vs. 6
rs3772172	SKIL	Cancer	3	32		50:50	2 vs. 6
rs1051266	SLC19A1	Encode	21	41		50:50	10 vs. 4
rs1051298	SLC19A1	Encode	21	42		50:50	0 vs. 28
rs419291	SLC22A4	Encode	5	30		55:45	7 vs. 8
rs671473	SLC22A5	Encode	5	25		55:45	0 vs. 10
rs11806	SLC25A17	Encode	22	35		55:45	20 vs. 4
rs11173459	SLC2A13	Encode	12	15		NA	NA
rs17178345	SLC37A1	Encode	21	19		55:45	0 vs. 16
rs2839550	SLC37A1	Encode	21	14		NA	NA
rs1042173	SLC6A4	Encode	17	44		50:50	25 vs. 1
rs7420	SLC9A3R1	Encode	17	44		50:50	10 vs. 4
rs2290725	SMARCA3	Cancer	3	31		50:50	5 vs. 14
rs3182285	SMARCA3	Cancer	3	31		50:50	6 vs. 11
rs7935	SMARCA4	Cancer	19	21		50:50	0 vs. 5
rs11090285	SMARCB1	Cancer	22	16		50:50	0 vs. 3
rs5030612	SMARCB1	Cancer	22	15		NA	NA
rs5030612	SMARCB1	Encode	22	14		50:50	1 vs. 2
rs6007010	SMC1L2	Encode	22	13		NA	NA
rs1061280	SMO	Cancer	7	23		55:45	5 vs. 7
rs1061285	SMO	Cancer	7	23		50:50	1 vs. 1
rs3824	SMO	Cancer	7	7		NA	NA
rs5997872	SMTN	Encode	22	17		50:50	1 vs. 4
rs705	SNRPN	Cancer	15	42	High variance	95:05	18 vs. 24
rs1308137	SNX27	Encode	1	37		50:50	2 vs. 3
rs1054204	SPARC	Cancer	5	36		50:50	4 vs. 11
rs1059279	SPARC	Cancer	5	15		55:45	1 vs. 8
rs1059829	SPARC	Cancer	5	34		50:50	7 vs. 8
rs1057233	SPI1	Cancer	11	41		50:50	12 vs. 6
rs1053004	STAT3	Cancer	17	30		50:50	1 vs. 14
rs1053023	STAT3	Cancer	17	32		50:50	0 vs. 7
rs3744483	STAT3	Cancer	17	32		50:50	0 vs. 14
rs3198502	STAT5A	Cancer	17	32		50:50	2 vs. 1
rs2822640	STCH	Encode	21	27		50:50	7 vs. 5
rs13309883	STEAP2	Encode	7	36		55:45	6 vs. 11
rs194520	STEAP2	Encode	7	17		55:45	2 vs. 9
rs194524	STEAP2	Encode	7	21		55:45	7 vs. 3
rs414781	SUHW2	Encode	22	16		NA	NA
rs2329902	SUMO3	Encode	21	25		NA	NA
rs235292	SUMO3	Encode	21	9		NA	NA
rs1049164	SYK	Cancer	9	31		55:45	9 vs. 8
rs2290887	SYK	Cancer	9	14		NA	NA
rs2290890	SYK	Cancer	9	14		NA	NA
rs13554	SYNJ1	Encode	21	30		50:50	0 vs. 8
rs2254562	SYNJ1	Encode	21	17		55:45	0 vs. 14
rs844985	SYNJ1	Encode	21	11		NA	NA
rs4823086	TBC1D10A	Encode	22	12		55:45	1 vs. 3
rs15411	TBC1D22A	Encode	22	31		55:45	0 vs. 31
rs2070116	TCF20	Encode	22	33		50:50	0 vs. 7
rs9611746	TCF20	Encode	22	35		50:50	11 vs. 0

rs	Gene	Panel	chr	# Hets	Variance & Mean	meanAI	A vs. B
rs6567211	TCF4	Cancer	18	38		50:50	0 vs. 21
rs8766	TCF4	Cancer	18	38		50:50	0 vs. 19
rs10418	TCN2	Encode	22	26		55:45	12 vs. 10
rs1801198	TCN2	Encode	22	33		55:45	6 vs. 6
rs9621049	TCN2	Encode	22	6		NA	NA
rs6517105	TCP10L	Encode	21	11		NA	NA
rs4135113	TDG	Cancer	12	16		NA	NA
rs3189859	TDGF1	Cancer	3	7		NA	NA
rs938886	TEP1	Cancer	14	27		55:45	0 vs. 24
rs4428611	TES	Encode	7	22		NA	NA
rs4710	TES	Encode	7	37		55:45	0 vs. 37
rs4710	TES	Cancer	7	43		50:50	0 vs. 26
rs9429072	TESK2	Cancer	1	37		50:50	4 vs. 12
rs2073157	TFEB	Encode	6	36		50:50	5 vs. 5
rs2273068	TFEB	Encode	6	18		50:50	1 vs. 0
rs225334	TFF2	Encode	21	17		65:35	8 vs. 4
rs7297	TFG	Cancer	3	48		50:50	22 vs. 3
rs2032574	TFIP11	Encode	22	15		50:50	7 vs. 1
rs17788379	TFRC	Cancer	3	9		NA	NA
rs406271	TFRC	Cancer	3	33		60:40	29 vs. 0
rs868	TGFBR1	Cancer	9	33		50:50	4 vs. 5
rs902	TGFBR3	Cancer	1	31		50:50	6 vs. 7
rs2292305	THBS1	Cancer	15	13	Mean shifted	NA	NA
rs17062695	TJP2	Cancer	9	20		55:45	0 vs. 14
rs3812536	TJP2	Cancer	9	38		50:50	0 vs. 21
rs1065769	TK1	Cancer	17	29		50:50	2 vs. 3
rs10771314	TM7SF3	Cancer	12	24		NA	NA
rs4856	TM7SF3	Cancer	12	24		NA	NA
rs2838475	TMEM1	Encode	21	13		50:50	0 vs. 7
rs1532	TMEM50B	Encode	21	21		55:45	0 vs. 15
rs2834216	TMEM50B	Encode	21	21		50:50	6 vs. 1
rs3743887	TMEM8	Encode	16	5		NA	NA
rs2839500	TMPRSS3	Encode	21	13	High variance	NA	NA
rs4820268	TMPRSS6	Encode	22	38		60:40	25 vs. 1
rs3093665	TNF	Cancer	6	7		NA	NA
rs3093704	TNFAIP1	Cancer	17	10		NA	NA
rs1047275	TNFRSF10B	Cancer	8	44		55:45	0 vs. 41
rs11135695	TNFRSF10B	Cancer	8	37		60:40	0 vs. 32
rs7957	TNFRSF10D	Cancer	8	24		50:50	0 vs. 0
rs7290134	TNFRSF13C	Encode	22	27		50:50	1 vs. 9
rs1061622	TNFRSF1B	Cancer	1	26		50:50	2 vs. 6
rs1061624	TNFRSF1B	Cancer	1	45		55:45	3 vs. 27
rs1061628	TNFRSF1B	Cancer	1	35		50:50	5 vs. 14
rs1131532	TNFSF10	Cancer	3	30		50:50	0 vs. 10
rs1131535	TNFSF10	Cancer	3	40		50:50	2 vs. 18
rs1131542	TNFSF10	Cancer	3	30		50:50	9 vs. 6
rs12485008	TNRC6B	Encode	22	5		NA	NA
rs6001734	TNRC6B	Encode	22	32		50:50	2 vs. 11
rs4626	TOB1	Cancer	17	28		50:50	5 vs. 11
rs202641	TOB2	Encode	22	29		NA	NA
rs5750668	TOMM22	Encode	22	25		50:50	2 vs. 0

rs	Gene	Panel	chr	# Hets	Variance & Mean	meanAI	A vs. B
rs13695	TOP2A	Cancer	17	29		50:50	0 vs. 16
rs1058298	TP53BP1	Encode	15	41		50:50	4 vs. 14
rs2602141	TP53BP1	Encode	15	40		50:50	1 vs. 11
rs689647	TP53BP1	Encode	15	8		NA	NA
rs690367	TP53BP1	Encode	15	40		55:45	0 vs. 38
rs2181484	TP73	Encode	1	15		NA	NA
rs3753565	TPR	Cancer	1	23		50:50	1 vs. 10
rs1131877	TRAF3	Cancer	14	27		50:50	4 vs. 5
rs10769167	TRIM5	Encode	11	15		50:50	2 vs. 1
rs3740995	TRIM5	Encode	11	18		50:50	5 vs. 1
rs3740996	TRIM5	Encode	11	11		50:50	2 vs. 3
rs7120209	TRIM6	Encode	11	13		60:40	7 vs. 4
rs7927012	TRIM6	Encode	11	30		65:35	10 vs. 12
rs6706	TRIP6	Cancer	7	12		55:45	11 vs. 0
rs13585	TRMU	Encode	22	21		50:50	3 vs. 2
rs1051771	TSC2	Cancer	16	13		50:50	0 vs. 10
rs635608	TSEN34	Encode	19	15		50:50	4 vs. 1
rs2839531	TSGA2	Encode	21	12		NA	NA
rs2839536	TSGA2	Encode	21	19		60:40	0 vs. 11
rs1053808	TTC3	Encode	21	43		50:50	7 vs. 1
rs1053966	TTC3	Encode	21	42		50:50	3 vs. 4
rs2835574	TTC3	Encode	21	38		50:50	3 vs. 0
rs2835665	TTC3	Encode	21	26		50:50	8 vs. 0
rs9974286	TTC3	Encode	21	38		50:50	8 vs. 0
rs2076155	TTLL1	Encode	22	6		NA	NA
rs5759125	TTLL1	Encode	22	12		NA	NA
rs12159975	TUBA8	Encode	22	9		NA	NA
rs1053395	TUBB4	Cancer	19	32		60:40	2 vs. 21
rs3099129	TUBB4	Cancer	19	11		NA	NA
rs11703226	TUBGCP6	Encode	22	28		NA	NA
rs1044732	TXNRD2	Encode	22	12		55:45	0 vs. 12
rs5992495	TXNRD2	Encode	22	19		55:45	0 vs. 12
rs2790	TYMS	Cancer	18	12		55:45	10 vs. 0
rs699517	TYMS	Cancer	18	27		50:50	0 vs. 16
rs6554	UBA52	Cancer	19	50		50:50	5 vs. 3
rs220159	UMODL1	Encode	21	23		55:45	2 vs. 9
rs7280633	UMODL1	Encode	21	19		60:40	0 vs. 14
rs1043524	UNC84B	Encode	22	26		50:50	0 vs. 18
rs2072797	UNC84B	Encode	22	19		55:45	0 vs. 18
rs1314	UPB1	Encode	22	21		50:50	14 vs. 1
rs1034123	USP16	Encode	21	9		NA	NA
rs2274802	USP16	Encode	21	37		50:50	0 vs. 1
rs3180408	USP18	Encode	22	33		55:45	3 vs. 8
rs2185798	USP49	Encode	6	41		50:50	10 vs. 13
rs1010	VAMP8	Cancer	2	44		50:50	8 vs. 2
rs1058588	VAMP8	Cancer	2	44		50:50	0 vs. 33
rs3731828	VAMP8	Cancer	2	38		50:50	0 vs. 17
rs602990	VAV2	Cancer	9	37		55:45	21 vs. 3
rs11887	VBP1	Cancer	X	12	High variance	80:20	6 vs. 5
rs2010963	VEGF	Cancer	6	31		50:50	9 vs. 0
rs25648	VEGF	Cancer	6	7		50:50	0 vs. 1

rs	Gene	Panel	chr	# Hets	Variance & Mean	meanAI	A vs. B
rs3025039	VEGF	Cancer	6	14		50:50	0 vs. 10
rs7294	VKORC1	Encode	16	36		50:50	0 vs. 1
rs2248490	WDR4	Encode	21	46		55:45	20 vs. 4
rs3746939	WDR4	Encode	21	17		55:45	1 vs. 8
rs6586250	WDR4	Encode	21	25		50:50	1 vs. 6
rs9977828	WDR4	Encode	21	12		NA	NA
rs1049403	WEE1	Cancer	11	30		NA	NA
rs2024233	WNT2	Encode	7	24	High variance	65:35	7 vs. 9
rs2024233	WNT2	Cancer	7	15	High variance	65:35	2 vs. 4
rs1060180	WRB	Encode	21	20		50:50	12 vs. 3
rs1346044	WRN	Cancer	8	35		50:50	1 vs. 6
rs1800392	WRN	Cancer	8	33		50:50	0 vs. 15
rs2228000	XPC	Cancer	3	25		NA	NA
rs2229090	XPC	Cancer	3	35		50:50	0 vs. 11
rs2470352	XPC	Cancer	3	33		50:50	2 vs. 1
rs1799782	XRCC1	Cancer	19	9		60:40	0 vs. 9
rs3547	XRCC1	Cancer	19	43		50:50	12 vs. 6
rs3218536	XRCC2	Cancer	7	8		55:45	0 vs. 4
rs1056503	XRCC4	Cancer	5	14		NA	NA
rs2035990	XRCC4	Cancer	5	11		NA	NA
rs1051677	XRCC5	Cancer	2	12		NA	NA
rs1051685	XRCC5	Cancer	2	15		NA	NA
rs207906	XRCC5	Cancer	2	17		55:45	0 vs. 17
rs1060922	YES1	Cancer	18	34		55:45	24 vs. 0
rs1061035	YES1	Cancer	18	16		NA	NA
rs2278699	ZAP70	Cancer	2	3		75:25	1 vs. 0
rs910796	ZBED4	Encode	22	19		NA	NA
rs4822021	ZC3H7B	Encode	22	31		55:45	19 vs. 0
rs5751084	ZC3H7B	Encode	22	30		55:45	0 vs. 23
rs2070132	ZNF146	Cancer	19	29		50:50	4 vs. 7
rs4806293	ZNF146	Cancer	19	27		55:45	0 vs. 18
rs11041108	ZNF215	Cancer	11	32		55:45	9 vs. 7
rs2239730	ZNF215	Cancer	11	41		60:40	17 vs. 6
rs2239731	ZNF215	Cancer	11	44		60:40	6 vs. 17
rs11088101	ZNF294	Encode	21	20		50:50	2 vs. 3
rs2245431	ZNF294	Encode	21	22		NA	NA
rs2254796	ZNF294	Encode	21	39		50:50	1 vs. 3
rs11702690	ZNF295	Encode	21	10		NA	NA
rs2298265	ZNF687	Encode	1	21		55:45	13 vs. 0
rs9612413	ZNF70	Encode	22	8		NA	NA
rs4020	ZNF74	Encode	22	32		50:50	14 vs. 1
rs2241666	ZWINT	Cancer	10	44		50:50	5 vs. 1
